# Mysterious inhibitory cell regulator investigated and found likely to be secretogranin II related

**DOI:** 10.7717/peerj.3833

**Published:** 2017-10-13

**Authors:** John E. Hart, Iain J. Clarke, Gail P. Risbridger, Ben Ferneyhough, Mónica Vega-Hernández

**Affiliations:** 1 Endocrine Pharmaceuticals, Tadley, Hampshire, UK; 2 Department of Physiology, Neuroscience Program, Monash Biomedical Discovery Institute, Monash University, Clayton, VIC, Australia; 3 Department of Anatomy and Developmental Biology, Biomedical Discovery Institute, Monash University, Clayton, VIC, Australia; 4 Systems Biology Laboratory UK, Abingdon, Oxfordshire, UK; 5 Department of Zoology, Lawrence Laboratory, University of Cambridge, Cambridge, Cambridgeshire, UK

**Keywords:** Hormone, Tissue reduction, Hypothalamus, Granin, Fruit fly, Q9W2X8, Macrogranin I

## Abstract

In the context of a hunt for a postulated hormone that is tissue-mass inhibiting and reproductively associated, there is described probable relatedness to a granin protein. A 7–8 kDa polypeptide candidate (gels/MS) appeared in a bioassay-guided fractionation campaign involving sheep plasma. An *N*-terminal sequence of 14 amino acids was obtained for the polypeptide by Edman degradation. Bioinformatics and molecular biology failed to illuminate any ovine or non-ovine protein which might relate to this sequence. The *N*-terminal sequence was synthesized as the 14mer EPL001 peptide and surprisingly found to be inhibitory in an assay in vivo of compensatory renal growth in the rat and modulatory of nematode fecundity, in line with the inhibitory hormone hypothesis. Antibodies were raised to EPL001 and their deployment upheld the hypothesis that the EPL001 amino acid sequence is meaningful and relevant, notwithstanding bioinformatic obscurity. Immunohistochemistry (IHC) in sheep, rodents and humans yielded staining of seeming endocrine relevance (e.g. hypothalamus, gonads and neuroendocrine cells in diverse tissues), with apparent upregulation in certain human tumours (e.g. pheochromocytoma). Discrete IHC staining in *Drosophila melanogaster* embryo brain was seen in glia and in neuroendocrine cells, with staining likely in the corpus cardiacum. The search for the endogenous antigen involved immunoprecipitation (IP) followed by liquid chromatography and mass spectrometry (LC–MS). Feedstocks were PC12 conditioned medium and aqueous extract of rat hypothalamus—both of which had anti-proliferative and pro-apoptotic effects in an assay in vitro involving rat bone marrow cells, which inhibition was subject to prior immunodepletion with an anti-EPL001 antibody—together with fruit fly embryo material. It is concluded that the mammalian antigen is likely secretogranin II (SgII) related. The originally seen 7–8 kDa polypeptide is suggested to be a new proteoform of secretogranin II of ∼70 residues, SgII-70, with the anti-EPL001 antibody seeing a discontinuous epitope. The fly antigen is probably Q9W2X8 (UniProt), an uncharacterised protein newly disclosed as a granin and provisionally dubbed macrogranin I (MgI). SgII and Q9W2X8 merit further investigation in the context of tissue-mass inhibition.

## Introduction

A tissue-mass inhibitory hormone has been postulated, having gonadal and hypothalamic aspects (Hart, 2014), potentially filling a gap in scientific knowledge as to how the size of internal organs is constrained. In the rat there is pituitary hypertrophy after castration. This has been interpreted as due to the loss of a gonadal inhibitor, a concept that in the female rat has been extended to describe a reproductively related general anti-organotrophic hormonal effect. In an attempt to isolate the hormonal influence a prototype purification featured a bioassay-guided fractionation campaign based on ultrafiltration, gel filtration and anion exchange chromatography (Hart, 2003). The starting material for the fractionation was ovarian vein plasma from adult sheep (*Ovis aries*) and the bioassay involved adult female rats (*Rattus norvegicus*) treated for four days and subject to a terminal organ weight analysis (Hart, 1990). The index of activity was organ-mass reduction, as affecting the heart, kidneys, uterus, etc. (Hart, 2003). Later the bulk feedstock was jugular vein plasma from ovary-intact ewes, with focus on a 3–30 kDa fraction (Hart, 2013). Trypsin partially reduced activity in an assay in vitro of bone marrow stem cell proliferation, supporting the proteinaceous character of the inhibitory component; heat likewise. Ammonium sulphate precipitated the activity. This plasma fraction yielded a polypeptide candidate with a molecular weight of ∼7.5 kDa in MALDI-TOF mass spectrometry (Hart, 2013), representing ∼70 amino acids (aa). This polypeptide was dubbed Circa-70. An electroeluted gel band of Circa-70 proved inhibitory in the in vitro bioassay. An *N*-terminal sequence of 14 aa was obtained from automated step-wise Edman degradation (Applied Biosystems/PROCISE, Foster City, CA, US), upon which bioinformatics was unable to shed any immediate light. The sequence was synthesized to provide a 14mer peptide, which was given the proprietary designation EPL001. This proved to have dose-dependent tissue-reducing properties in an in vivo model system involving compensatory renal growth in the unilaterally nephrectomised rat (Haylor et al., 2009). Conversely, an antibody to EPL001 exaggerated compensatory renal growth, suggesting that an endogenous inhibitor had been neutralized. In periodic follow-up searches BLAST continued to yield no convincing hits. The 14 aa sequence also proved resistant to multiple attempts at molecular biological elucidation, based on probing DNA and RNA libraries. Molecular modelling coupled with quantitative structure activity relationship (QSAR) analysis, as used previously (Davies et al., 2015), pointed to EPL001’s *C*-terminal FNNI as a potentially key binding site, representing a lipophilic/hydrophobic cleft. An anti-EPL001 antibody targeted at the *C*-terminal end of the peptide has accordingly been deployed here in an attempt to purify molecular species that bound to the antibody in material from the rat and sheep and, for a cross-phylum comparison, the fruit fly *D. melanogaster*.

To test the inhibitory hormone hypothesis requires purified endogenous candidate material or a synthetic version thereof. This could be used to reverse post-castration pituitary hypertrophy in the rat for example. The present exercise in contrast is an antecedent study. It asks, is the purified polypeptide with the EPL001 amino acid sequence even a credible candidate and, if so, what does it relate to endogenously? The hypothesis is that the EPL001 sequence is meaningful and relevant, the null hypothesis that it isn’t. The strategy here is to use anti-EPL001 antibodies to address potential meaningfulness and relevance with the aid of IHC to localise whatever is being seen by the antibodies; an in vitro assay to stand in for the in vivo assays for bioactivity assessments, using prior immunodepletion for factor elimination; western blotting to find the molecular weights of endogenous antigens; and immunoprecipitation (IP)/liquid chromatography and mass spectrometry (LC–MS) for identification of the endogenous antigen or antigens, with bioinformatics in support. This report proposes that the endogenous mammalian antigen of the anti-EPL001 antibodies is likely secretogranin II (SgII) related and that the fly antigen is probably an uncharacterised unnamed protein identified in the UniProt database (http://www.uniprot.org/) as Q9W2X8, which can itself be recognized anew as a granin.

## Materials and Methods

### Peptide

The 14mer peptide EPL001, having the aa sequence MKPLTGKVKEFNNI (Hart, 2013), and sections thereof, were synthesized by a commercial supplier (Peptide Protein Research, Fareham, UK).

### Antibodies

For the rabbit polyclonal antiserum a cysteine EPL001 peptide was synthesized conjugated at its *N*-terminus with the carrier protein KLH (Hart, 2013). Rabbits at a supplier (Eurogentec, Liège, Belgium) were injected subcutaneously with the antigen (400 μg protein) in PBS mixed with an equal volume of complete Freund’s adjuvant followed by subsequent injections at monthly intervals in Freund’s incomplete adjuvant. An ELISA dilution curve for the rabbit polyclonal antiserum (ER88) has been provided elsewhere (Haylor et al., 2009). A polyclonal antiserum (G530) was raised in goat (Monash University, Victoria, Australia) in a similar manner (Hart, 2013) and an antibody ELISA dilution curve obtained using the same standard methodology (Haylor et al., 2009). Titre was also established via IHC, using rat and ovine median eminence tissue (basal hypothalamus), and the ability to block antibody with EPL001 was determined on histological sections at 0.5 mg/ml. Partial epitope mapping involved dot blot analysis. Peptides were dissolved in PBS with and without 0.1% SDS to a concentration of 100 μM, with 5 μl dotted onto PVDF membrane pre-equilibrated in transfer buffer (25 mM Tris, 192 mM glycine, 20% v/v methanol) and incubated at room temperature for 1 h. The blot was then processed as below (see westerns blots). The identity of endogenous antigens were investigated via ovine IHC co-localisation studies using antibodies to known hypothalamic factors.

### Immunohistochemistry

Multiple tissues were investigated in IHC using a human array (SuperBioChips Laboratories, Seoul, Korea) having both normal (AA8) and tumour (BB4) sections. Epitopes were revealed by microwaving in 0.01 M citrate buffer (pH 6) and immunohistochemical staining undertaken using the Dako Autostainer Universal Staining System (Dako A/S, Glostrup, Denmark). Briefly, sections were treated with peroxidase blocking reagent (Envision System; Dako, Glostrup, Denmark) (15 min) followed by CAS block (Zymed, Invitrogen, Paisley, UK) for non-specific binding (30 min). Sections were incubated with a rabbit polyclonal antiserum to EPL001 at a dilution of 1/200 (1 h, room temperature). The secondary antibody, an anti-rabbit immunoglobulin conjugated to a peroxidase labelled polymer (Envision System; Dako, Glostrup, Denmark), was incubated (15 min, room temperature) followed by the addition of 3,3′-diaminobenzidine tetrahydrochloride (DAB; Dako, Glostrup, Denmark) (5 min). Reactions were stopped in water and sections counterstained with Meyer’s haematoxylin. Preabsorption studies were undertaken using an incubating concentration of EPL001 at 0.5 mg/ml. Staining for chromogranin A (CgA), a protein associated with chromaffin granules, was used on serial sections as a marker of neuroendocrine cells (Taupenot, Harper & O’Connor, 2003). Hypothalami and other central and peripheral tissues were harvested post-mortem from Corriedale ewes (*Ovis aries*) of typically five to six years of age, which had been maintained under natural conditions at the Monash University Sheep Facility, Werribee, Victoria, Australia. Husbandry and termination were carried out according to the guidelines established by the Australian Prevention of Cruelty to Animals Act 1986 and these procedures and all others in relation to the Australian research in this report were in accordance with the guidelines of and approved by (Ref: MARP/2012/012) the Monash University Animal and Human Ethics Committees. Tissues were fixed using 4% paraformaldehyde in saline, freshly prepared. Sections for IHC were obtained and processed in the standard way, using frozen sections cut on a cryostat, citrate buffer antigen retrieval, DAB visualisation and the anti-EPL001 rabbit and goat polyclonal antisera. For the rat hypothalamus (B1 in the mammalian IHC images) the rat brain was perfused with 4% paraformaldehyde and sectioned coronally at 40 μm. Sections were blocked with normal rabbit serum. The primary goat antiserum was diluted at 1/1,000. Incubation was at 4 °C for 48 h. Biotinylated rabbit anti-goat secondary (1/500 for 1 h) was used along with streptavidin-HRP (1/500 for 1 h). Colour was developed using DAB (15 min; Roche, Basel, Switzerland). Fruit flies (*D. melanogaster*) were used from a colony maintained at the Department of Zoology, University of Cambridge, UK. Wild type fly embryos were collected from apple agar plates and dechorionised with 50% bleach. They were then fixed with 4% paraformaldehyde for 15 min and the vitelline membrane removed using a heptane/methanol solution. Embryos were hydrated and permeabilised with PBS-Triton-X 0.3% and incubated overnight with the primary anti-EPL001 goat antiserum, used at a dilution of 1/100. For preabsorption control studies antiserum at 5 mg/ml was incubated with a 10-fold excess of EPL001 peptide and incubated at room temperature for 1 h and then washed and incubated with a fluorescent secondary antibody (donkey-anti-goat-FITC). Images were obtained with a SP5 Leica confocal microscope. Antigen retrieval was not used. For comparative purposes, antibodies were used to the following markers: Repo (glia), Elav (neurons), Mef2 (mesoderm, heart), Prospero (lateral glia), Pointed P1 (stomatogastric nervous system) and Pericardin (heart).

### In vitro assay

Bone marrow cells, consisting of various cell types including mesenchymal stem cells and fibroblasts, were cultured from adult male rats (*Rattus norvegicus*, SD strain). These cells were passaged from a growing primary stock in a T75 tissue culture flask (Sarstedt) into a 48 well tissue culture plate (IWAKI), with a cell density of 1.5 × 10^5^/ml. The cell culture media, henceforth referred to as ‘supplemented α-MEMS,’ comprised α-MEMS media supplemented with 10% foetal bovine serum and 1% penicillin/streptomycin (all Life Technologies, Carlsbad, CA, USA). On the day of material addition to the cells, the supplemented α-MEMS was gently removed and fresh supplemented α-MEMS added, with 10% test material by volume, untreated controls receiving just fresh supplemented α-MEMS. All samples were filter sterilised using a Millex-GV 0.22 μM syringe filter unit (Merck, Kenilworth, NJ, USA). The cells were imaged for 24 h using an incubated Zeiss Axiovert 135M microscope with a Prior Proscan II motorised stage, at 37 °C with a 5% CO_2_ in air feed. Each well of the 48-well tissue culture plate was then imaged for 24 h every 5 min using Metamorph software (MDS Analytical Technologies, Wokingham, UK), with each well having four fields of view. The viability and cell numbers were reviewed by analysing the images captured over the 24 h period and cell counts were taken at selected time points using the Metamorph software. The experiments, including cell counting, were ‘blind,’ the experimenter only being apprised of sample identity after data analysis and preliminary reporting. Test materials were rat PC12 conditioned medium and aqueous extract of rat hypothalamus, with and without anion exchange chromatography and with and without immunodepletion involving the anti-EPL001 rabbit antiserum. To prepare PC-12 conditioned media, 5 × 10^5^ PC12 cells were seeded onto T25 flasks and grown for three days (unless otherwise stipulated) to a density of 3 × 10^6^, in RPMI 1640, 2 mM Glutamine, 2% Foetal Bovine Serum, and the media harvested. PC12 conditioned media was subjected to anion exchange chromatography as follows: a total of 50 ml of conditioned media was diluted five-fold with 20 mM phosphate buffer and applied to a 5 ml Hitrap Q HP column (GE Healthcare 17-1154-01), pre-equilibrated in the same buffer, and eluted in 20 column volumes with a 0–1 M NaCl gradient using an AKTA FPLC (GE Healthcare, Little Chalfont, UK). Relevant biological activity had been seen in early studies involving sheep material (serum, follicular fluid) in <30 kDa fractions eluting at 0.2 M NaCl (tissue shrinkage in vivo (Hart, 2003)) and 0.8 M NaCl (reduced tumour cell proliferation in vitro (Hart, 2013, Example 7a therein)), directing attention at these anionic exchange fractions in particular. The hypothalamic extract was prepared using hypothalami from 50 adult male rats (*Rattus norvegicus*, CD strain), with tissue preparation at source (Charles River, Margate, UK), from animals maintained in compliance with all relevant national and international regulations in high quality barrier facilities. The hypothalami were homogenised from frozen and suspended in 10 ml of 50 mM Tris pH 8.0 with Complete Protease Inhibitor Cocktail (Sigma Aldrich, Dorset, UK). This was centrifuged at 20,000*g* for 1 h, the supernatant retained, and the sedimentation step repeated. This extract, neat and after anion exchange chromatography (as above) was submitted for western blot analysis. For immunodepletion studies an additional 50 ml of PC12-conditioned media was produced and incubated overnight with 200 μl anti-EPL001 antiserum at 4 °C. Antibody was then removed using a protein G column (Pierce, Fischer Scientific, Loughborough, UK). In every case immunodepletions were carried out on 0.7 ml of sample with the addition of a preabsorption step whereby 50 μl of equilibrated protein G beads were added for 1 h with gentle agitation to remove endogenous immunoglobulin. EPL001 peptide was provided for preabsorption at 100 μM. The work was conducted under UK Home Office Project Licence PPL 30/2280 and Personal Licence 30/353 and while specific ethical approval was not required the research was performed in accordance with the guidelines of the Ethical Review Board of the University of Reading, UK, and within its purview.

### Western blots

Protein was transferred to Immobilon-P PVDF membrane (IPVH00010; Millipore, Watford, UK) from Lamelli-SDS gels and blocked with 3% milk powder, 1% BSA, 0.2% Tween, 100 mM NaCl, 50 mM Tris–HCl, pH 7.4. Goat primary antiserum was applied in blocking buffer at 1/400 dilution (or as otherwise stated) for 1 h at room temperature or overnight at 4 °C with gentle agitation. After washing, secondary antibody (Donkey anti-goat HRP, 2 mg/ml, ab6885; Abcam, Cambridge, UK) at 1/5,000 dilution (or as otherwise stated) was applied for 1 h. After washing in blocking buffer and finally in 50 mM Tris–HCl pH 7.4 they were developed using ECL substrate (Pierce, 32106) according to the manufacturer’s instructions. For dot blot analysis, peptides were dissolved in PBS to 100 μM, with 5 μl applied to the equilibrated membrane and treated as just described, with antiserum subjected to protein G purification. Serum (5 L) from the systemic blood of lambs (Monash University, Victoria, Australia) was dialysed against three changes of 50 mM Tris pH8.0 (25 L each), applied to a 150 Q Sepharose anionic exchange column (17051001; GE Healthcare, Little Chalfont, UK) and step-eluted with 400 mM NaCl, 50 mM Tris pH 8.0. The eluate was dialysed against 3 × 1 L 50 mM Tris pH 8.0 and applied to a 5 ml HiTrap FF Q Sepharose column (17515301; GE Healthcare, Little Chalfont, UK) from which protein was eluted in a 50 ml gradient from 0 to 1 M NaCl in 50 mM Tris pH 8.0. From every fifth 1 ml fraction 30 μl was analysed by 15% SDS PAGE under non-reducing conditions. Western blots were prepared with 1/5,000 goat polyclonal antiserum and 1/2,000 secondary (Donkey anti-goat HRP), 3 min exposure. PC12 conditioned medium, rat hypothalami and fruit fly embryos were obtained and prepared for western blotting as described elsewhere in this section. Note that before the adoption of the IP protocol, to be described next, a prior antigen capture procedure involved an immunoaffinity column, with rat hypothalamic material as feedstock. The methodology is reported here because it led to the identification of western bands at ∼7 kDa, sensitive to preabsorption. A Pierce Protein G IgG Plus Orientation Kit (44990; Thermo Scientific, Waltham, MA, USA) was used to make an antibody affinity column. Briefly, 2 ml of immobilised protein G 50% slurry was bound to saturation (approximately 10 mg) with antibody from goat antiserum raised against the peptide EPL001. Bound antibody was covalently linked to the matrix according to the manufacturer’s instructions. Hypothalami and pituitary glands were dissected out of a mixed population of 268 rats and rapidly frozen in liquid nitrogen. They were then ground to a paste using an homogeniser, resuspended in phosphate-buffered saline (PBS) solution containing a protease inhibitor cocktail and subjected to 3 × 20 s pulses of sonication. Insoluble material was removed by sedimentation at 23,200 g for 2 h. The supernatant was applied to the affinity column by gravity feed after which the column was washed in PBS and bound protein eluted in 1 ml batches with 0.1 M glycine pH 2.8. Fractions were analysed by 15% SDS PAGE and 10–20% tricine gradient gels, using non-reducing conditions, and western blotting using the same protein G-purified goat antibody at a dilution of 1/3,000 for both primary and secondary antibodies. Candidate bands were either excised for MS (Applied Biosystems 4700 MALDI mass spectrometer) or blotted onto membrane for *N*-terminal Edman degradation sequence analysis (Applied Biosytems Procise cLC, at University of Bristol, Bristol, UK).

## Immunoprecipitation

For IP anti-EPL001 goat polyclonal antibody was purified from serum using the Pierce Protein G IgG Plus Orientation Kit (Thermo Scientific, 44990) as directed. This was then further purified to remove amines using Zeba Spin Desalting columns (Thermo Scientific, 89889). Covalent coupling to resin and IP were carried out using a Pierce Direct IP Kit (Thermo Scientific, 26148) according to the manufacturer’s instructions except where stipulated. Briefly, for each IP 10 μg of purified antibody was mixed with 20 μl of equilibrated resin slurry and cross-linked with sodium cyanoborohydride at room temperature for 2 h. The reaction was quenched and the resin washed and equilibrated in IP Lysis/Wash solution (0.025 M Tris, 0.15 M NaCl, 0.001 M EDTA, 1% NP40, 5% glycerol; pH 7.4). The goat polyclonal antibody was also affinity purified using the peptide EPL001 covalently bound to Affi-Gel 10 (1536099; Bio-Rad, Watford, UK) according to the manufacturer’s instructions. Briefly, peptide (25 mg) was dissolved in 1 ml of 0.1 M HEPES buffer pH 8.0 and bound to 1 ml of Affi-Gel 10 beads (Bio-Rad). About 10 mg (2 ml) of purified and desalted polyclonal antibody was then applied and, after washing specifically bound antibody, was eluted in 0.1 M Glycine-HCl buffer pH 3.0 and immediately neutralised by the addition of Tris pH 8.0 to a final concentration of 50 mM. PC12 conditioned medium was collected as described above (see ‘In vitro assay’) and stored frozen for later use in IP, without formaldehyde fixation or antigen retrieval. Other samples were prepared for IP as follows. Three unfixed hypothalami from adult male rats (Sprague–Dawley, University of Bristol, Bristol, UK) were homogenised from frozen using a micro-pestle and mortar, and suspended in 1ml of IP lysis buffer with cOmplete Protease Inhibitor (Sigma Aldrich) and subjected to three 10 s bursts of sonication using a microprobe. This was then sedimented for 30 min at 20,000 g and 4 °C and the cleared lysate retained. Five hypothalami fixed in 4% formaldehyde from immature rats (three weeks old, CD strain, Charles River, Margate, UK; the speculation being that young animals are under hypothalamic reproductive braking, lifted at puberty (Hart, 2014)) and 20 formaldehyde-fixed embryos of *Drosophila* (see above, IHC) were first homogenised from frozen and then subjected to extraction and antigen retrieval using a method based on that described by Shi et al. (2006) using RIPA buffer or citrate buffer (Shi et al., 1993). RIPA was chosen for the fly preparation, as a comparison between RIPA and citrate using rat hypothalamic material showed that RIPA provided the cleaner preparation; see Identification. Homogenised tissue was re-suspended in either 1× RIPA buffer with additional SDS (25 mM Tris–HCl pH 7.6, 150 mM NaCl, 1% NP-40, 1% sodium deoxycholate, 2% SDS) or 10 mM sodium citrate, 0.05% Tween, pH 6.0, to a final volume of 1 ml and incubated for 20 min at 100 °C and then 60 °C for 2 h. Insoluble material was sedimented at 20,000 g for 30 min and the supernatant diluted four-fold with IP Lysis/Wash solution and retained for IP. For controls, identical IPs were carried out where Ig-bound resin was pre-incubated with 0.5 ml of 100 μM EPL001 in IP Lysis/Wash buffer for 1 h at room temperature with the excess removed and the resin washed (as above) before addition of the sample. For each IP 600 μl of sample was added to the prepared slurry in spin columns and incubated overnight at 4 °C with gentle agitation. Washes and elutions were carried out according to manufacturer’s instructions except for antigen retrieval samples which were washed in standard RIPA buffer (3 × 500 μl: 25 mM Tris–HCl pH 7.6, 150 mM NaCl, 1% NP-40, 1% sodium deoxycholate, 0.1% SDS). Samples were eluted in a total of 100 μl of 1 × non-reducing sample buffer (60 mM Tris–HCl, 1% SDS, 10% glycerol, tracking dye, pH 6.8). Fractions were analysed by western blotting, as above.

### Liquid chromatography and mass spectrometry

Samples were acrylamide gel-purified and the gel slice subjected to in-gel tryptic digestion using a DigestPro automated digestion unit (Intavis, Nottingham, UK). The resulting peptides were fractionated using an Ultimate 3000 nanoHPLC system in line with an LTQ-Orbitrap Velos mass spectrometer (Thermo Scientific) as described previously (Goggs et al., 2013; Palumbo et al., 2015). In brief, peptides in 1% (v/v) formic acid were injected onto an Acclaim PepMap C18 nano-trap column (Thermo Scientific). After washing with 0.5% (v/v) acetonitrile and 0.1% (v/v) formic acid, peptides were resolved on a 250 mm × 75 μm Acclaim PepMap C18 reverse phase analytical column (Thermo Scientific) over a 150 min organic gradient, using seven gradient segments (1–6% solvent B over 1 min, 6–15% B over 58 min, 15–32% B over 58 min, 32–40% B over 5 min, 40–90% B over 1 min, held at 90% B for 6 min and then reduced to 1% B over 1 min) with a flow rate of 300 nl min^−1^. Solvent A was 0.1% formic acid and solvent B was aqueous 80% acetonitrile in 0.1% formic acid. Peptides were ionized by nano-electrospray ionization at 2.1 kV using a stainless steel emitter with an internal diameter of 30 μm (Thermo Scientific) and a capillary temperature of 250 °C. Tandem mass spectra were acquired using an LTQ-Orbitrap Velos mass spectrometer controlled by Xcalibur 2.1 software (Thermo Scientific) and operated in data-dependent acquisition mode. The Orbitrap was set to analyse the survey scans at 60,000 resolution (at *m*/*z* 400) in the mass range *m*/*z* 300–2000 and the top 20 multiply charged ions in each duty cycle selected for MS/MS in the LTQ linear ion trap. Charge state filtering, where unassigned precursor ions were not selected for fragmentation, and dynamic exclusion (repeat count, 1; repeat duration, 30 s; exclusion list size, 500) were used. Fragmentation conditions in the LTQ were as follows: normalized collision energy, 40%; activation q, 0.25; activation time 10 min; and minimum ion selection intensity, 500 counts. The raw data files were processed using Proteome Discoverer software v1.4 (Thermo Scientific) and searched against appropriate UniProt species databases (http://www.uniprot.org) using the SEQUEST algorithm. Peptide precursor mass tolerance was set at 10 ppm and MS/MS tolerance was set at 0.8 Da. Search criteria included carbamidomethylation of cysteine (+57.0214) as a fixed modification and oxidation of methionine (+15.9949) as a variable modification. Searches were performed with full tryptic digestion and a maximum of one missed cleavage was allowed. The reverse database search option was enabled and all peptide data were filtered to satisfy false discovery rates (FDR) of 1% or 5%. For further analysis custom Perl scripts were used, run with ActiveState Perl v5.20.3 installed under Microsoft Windows 10 (64 bit).

## Results

### Epitope

**Figure 1:**
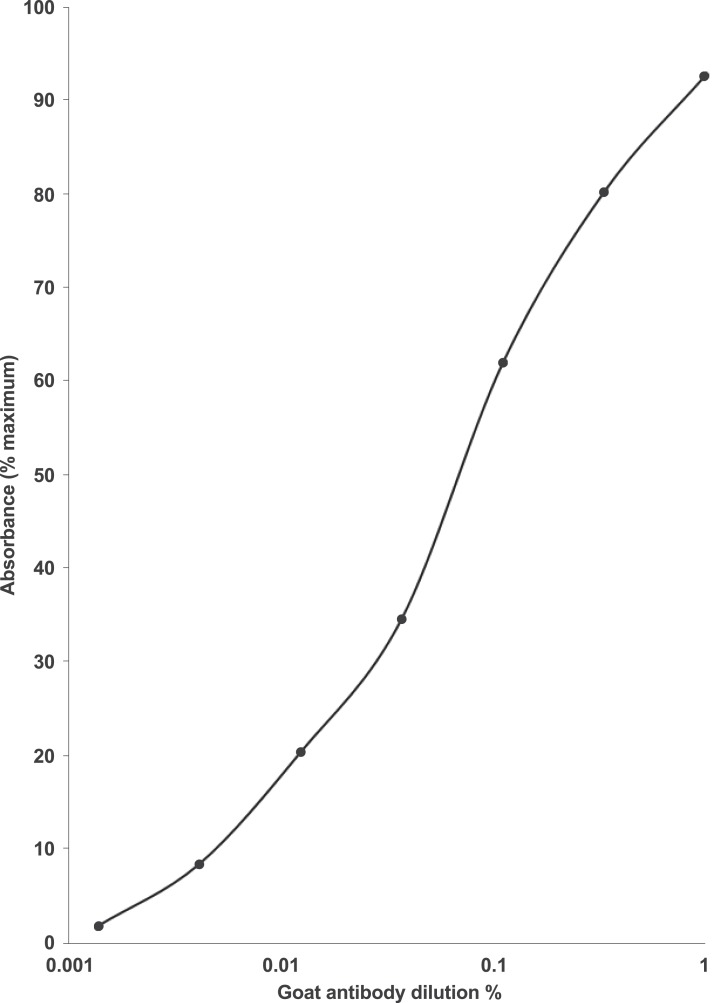
Dilution curve for the anti-EPL001 goat polyclonal antiserum G530. ELISA 96-well microtitre plates were coated with 10 ng/well of EPL001 and left overnight in the presence of the primary antibody at +4 °C, before incubation with a donkey anti-goat secondary antibody conjugated with horseradish peroxidase. Absorbance was measured at 450 nm. Relative absorbance (%) was expressed against the whole number absorbance value immediately above the maximum reading obtained with the antibody. The averages of duplicate values are presented.

**Figure 2:**
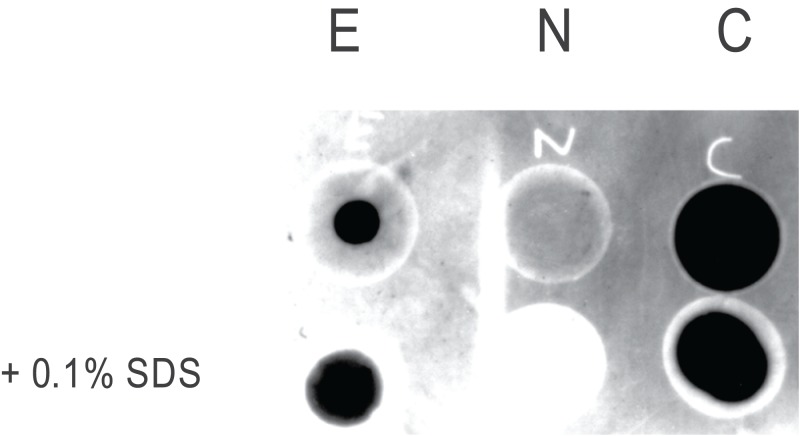
Dot blot epitope mapping of the goat anti-EPL001 antibody. The antibody was subjected to protein G purification. E = EPL001. N = *N*-terminus of EPL001 (i.e. MKPLTG). C = *C*-terminus of EPL001 (i.e. KVKEFNNI). Lower dots were +0.1% SDS.

An ELISA dilution curve for the rabbit anti-EPL001 antiserum has been provided previously (Haylor et al., 2009). For the antiserum (hereafter ‘antibody’) of the goat an ELISA dilution curve is shown in Fig. 1. Preabsorption of the goat antibody with EPL001 blocked staining in IHC and western blotting, as described below. (Preabsorption of the rabbit antibody has been determined in IHC and in the bone marrow cell assay, also as described below.) Dot blot analysis of three peptides—full length EPL001 (MKPLTGKVKEFNNI) and two component peptides, the *N*-terminal MKPLTG and the *C*-terminal KVKEFNNI—showed that the epitope or epitopes in EPL001 recognised by the goat polyclonal antibody resides or reside within the *C*-terminal section of the peptide, the addition of SDS (+0.1%) having no apparent effect (Fig. 2). A *C*-terminal locus for antibody binding is not unexpected as the peptide had been Cys-conjugated at the *N*-terminus to a carrier protein to facilitate antibody raising. Selective preabsorption has been achieved in western blotting with *C*-terminal EPL001 peptides but not *N*-terminal peptides (see below). A unitary *C*-terminal epitope has been confirmed elsewhere independently as probably KEFNNI (see ‘Discussion’). Sections of ovine hypothalamus were stained in IHC with antibodies to tyrosine hydroxylase (for dopaminergic elements), vGLUT2 (for glutamatergic elements), GnRH, CRF, AVP, GHRH and somatostatin, with no co-localisation observed with staining seen with the anti-EPL001 goat antibody.

### Localisation

**Figure 3:**
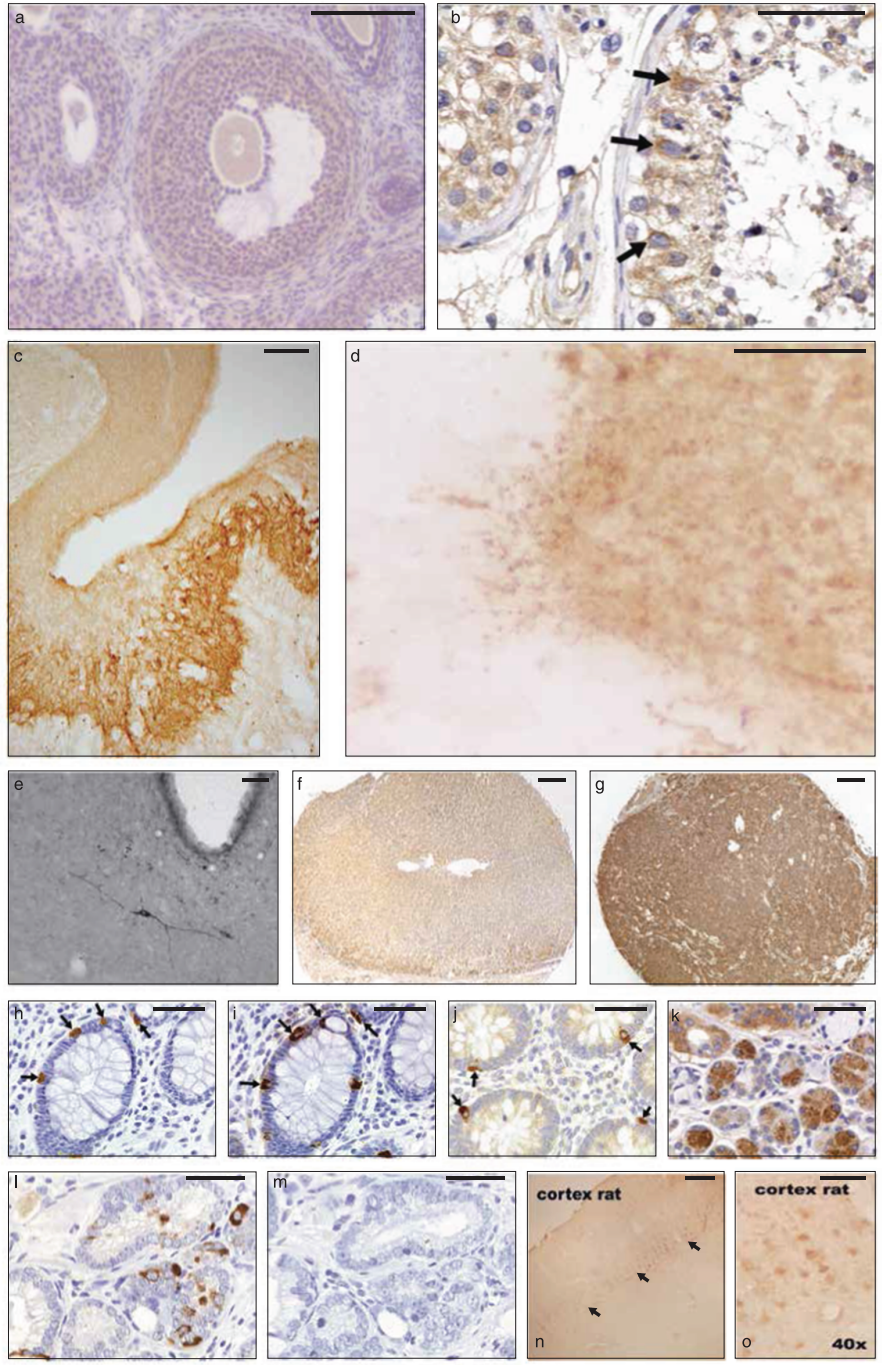
Mammalian IHC. Rabbit anti-EPL001 antiserum ER88 used unless otherwise stated, with dilution in brackets. (A) Ovary (rat) showing moderate staining (brown) (1/1,000). (B) Testis (human), epithelial germ cell staining arrowed (1/200). (C) Median eminence (sheep), staining of palisade (neuroendocrine) region (rabbit antiserum ER87, 1/1,000). (D) Median eminence (sheep, higher power), showing axonal beading (1/1,000). (E) Hypothalamus (rat), showing immunopositive neuron in the retrochiasmatic nucleus, with third ventricle appearing as white bay (goat antiserum, 1/1,000). (F) Adrenal (human, normal, medulla not shown), staining judged as negative (1/200). (G) Adrenal tumour (human pheochromocytoma), staining judged as strongly positive (1/200). (H) Colon (human) showing enteroendocrine cells (arrowed) (1/200). (I) Colon (human), serial section of H showing staining for CgA (1/200). (J) Appendix (human), showing neuroendocrine cells (arrowed) (1/200). (K) Salivary gland (human, ×40), ducts showing heavy staining (brown) (1/200). (L) Prostate (human) showing basal luminal neuroendocrine cells (1/200). (M) Prostate (human), serial section of L subject to preabsorption with EPL001 peptide at 0.5 mg/ml (1/200). (N) Cerebral cortex (rat), showing immunostaining in layer 5, arrowed (1/1,000). (O) Cerebral cortex (rat, higher power), showing layer 5 pyramidal cells (1/1,000). Scale bars ∼50 μm, except those for C, E, G and N, which are ∼200 μm.

**Figure 4:**
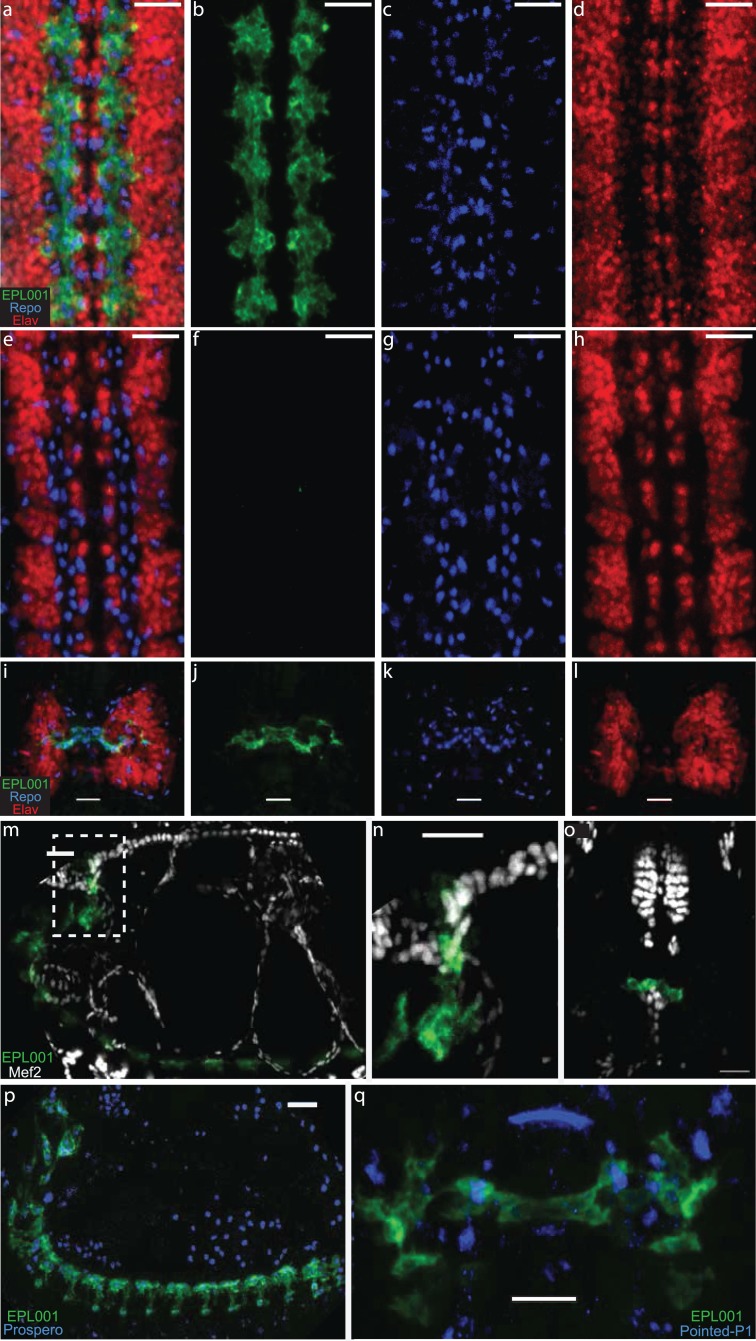
Fruit Fly IHC. Goat anti-EPL001 antiserum G530 used throughout, at a dilution of 1/100, staining in green; Repo marker in blue and Elav marker in red. Scale bars 20 μm throughout. (A) Fruit fly embryo ventral nerve cord, viewed from ventral side, merged image of anti-EPL001 antibody, Repo and Elav. (B) Anti-EPL001 antibody channel only. (C) Repo channel only. (D) Elav channel only. (E) Preabsorption control with EPL001 peptide added, merged image (as A). (F) Anti-EPL001 antibody channel only. (G) Repo channel only. (H) Elav channel only. (I) Brain, viewed from dorsal side of embryo, merged image of three stains (as A). (J) Anti-EPL001 antibody channel only. (K) Repo channel only. (L) Elav channel only. (M) Lateral view of embryo, with anti-EPL001 antibody in green and Mef2, which stains heart and mesoderm, in white. (N) Zoom of inset dotted area of M. (O) Ventral view of embryo, staining as M, with heart seen abutting the anti-EPL001 antibody staining of brain and pharynx. (P) Lateral view of embryo, staining with anti-EPL001 antibody and Prospero, a lateral glia marker, in blue. (Q) Brain region, dorsal view, showing partial co-localisation of anti-EPL001 antibody with Pointed P1, a stomatogastric marker, in blue.

Immunohistochemistry was used to determine the tissue distribution of the endogenous antigen or antigens of the anti-EPL001 antibodies. Images of stained tissue sections are provided as Figs. 3 and 4, with additional images also available (see Supplementary IHC Images). No staining was seen with pre-immune serum. Preabsorption of antibodies with the EPL001 peptide blocked staining in human prostate (Figs. 3L and 3M), human kidney (Haylor et al., 2009, and Supplementary IHC Images) and fruit fly embryo (Fig. 4F), for example. Staining for CgA was used as a marker on serial sections to define neuroendocrine cells, with focal staining in line with expectations (Human Protein Atlas, http://www.proteinatlas.org/, gene *CHGA*). Neuroendocrine staining in a human tissue array was seen in pancreas, stomach (body and antrum), duodenum, small bowel, appendix (Fig. 3J), colon (H and I), rectum and prostate (L). Staining was also judged strongly positive in salivary gland ducts (K) and in testis (B, Leydig cells and spermatogonia). Staining judged to be weakly positive was seen in liver, gall bladder, tonsil, kidney (localised to tubular cells in the cortex and medulla, including the collecting duct (Haylor et al., 2009)) and endometrium (proliferative and secretory). Human tissues judged as negative were: skin, subcutaneous fat, breast, spleen, lymph node, skeletal muscle, nasal mucus, lung, bronchus, heart, oesophagus, stomach (smooth muscle), urinary bladder, seminal vesicle, myometrium, uterine cervix, salpinx, ovarian stroma, placenta (mature and mid-trimester villae and cord), thyroid, thymus and cerebellum.

In separate series immunochemically stained cells were seen in sheep ovary (corpus luteum), rat ovary (moderate staining in theca, granulosa, follicle cells and follicular fluid; Fig. 3A), mouse placenta and confirmed in human prostate (focal basal luminal cells), the last-mentioned including benign prostatic hyperplasia (BPH) and prostatic carcinoma. Immunostaining was clearly apparent in the sheep hypothalamus (C and D), with individual neurons staining in the lateral and ventromedial hypothalamus and preoptic area, for example, and heavy staining visible in the palisade (neuroendocrine) region of the median eminence (C), with axonal beading (D). Staining in the median eminence was unaffected by whether the ewe was ovariectomised or intact. Individual neurons stained in the thalamus, the diagonal band of Broca and the cerebral cortex (pyramidal cells, layer 5; N and O). Staining was not seen in the pituitary. In a further series involving the hypothalamus of the rat, staining was visible in the arcuate nucleus, with individual neurons also staining in the retrochiasmatic nucleus (E). In a human tumour tissue array an adrenal pheochromocytoma section stained much more heavily than did normal adrenal in the non-tumour tissue array (G vs F). Hence the use of rat PC12 pheochromocytoma cells in the present study, as an inhibitor ‘factory.’ Adrenal cortical carcinoma was also strongly positive (for prostatic carcinoma, see above). A pattern of strongly positive staining in tumours versus negative staining in the corresponding normal tissue was upheld for the following tissues: skin (melanoma and squamous cell), breast (residual infiltrating duct carcinoma, invasive papillary carcinoma and invasive lobular carcinoma), lymph node (metastasis from the stomach, but lymphoma was negative), oesophagus (squamous cell carcinoma) and thyroid (minimally invasive follicular carcinoma, but papillary carcinoma and thymoma were negative). Examples where strong staining in normal tissue were matched by strong staining in tumour sections were salivary gland (submandibular salivary duct carcinoma), colon (adenocarcinoma), rectum (carcinoma) and testis (seminoma). Also strongly positive was soft tissue (liposarcoma, but soft tissue neurofibromatosis was negative), kidney (low-grade renal cell carcinoma, but transitional renal cell carcinoma and full renal cell carcinoma were only weakly positive) and liver (cholangiocarcinoma, but hepatocellular carcinoma was negative). Weakly positive staining tumours were bone (osteosarcoma and chondrosarcoma, but giant cell tumour negative), larynx (squamous cell carcinoma), lung (adenocarcinoma, squamous cell carcinoma and small cell carcinoma), gall bladder (carcinoma), pancreas (adenocarcinoma), stomach (moderately differentiated carcinoma and signet ring carcinoma; negative were poorly differentiated stomach carcinoma, malignant stomach lymphoma and borderline stromal tumour), small intestine (malignant stromal tumour), appendix (pseudomyxoma peritonia), urinary bladder (poorly differentiated carcinoma and high-grade transitional cell carcinoma) and uterus (endometrial carcinoma and squamous cell carcinoma of the cervix, but negative were leiomyosarcoma and leiomyoma). Negative staining tumours not already mentioned were nasal cavity (inverted papilloma), parotid gland (pleomorphic adenoma), ovary (endodermal sinus tumour and fibrothecoma) and brain (meningioma and glioblastoma multiforme).

As regards the fruit fly embryo, the anti-EPL001 goat antibody stained glial cells in the embryonic ventral cord during late embryogenesis stage 16 (Figs. 4A and 4B). Staining was also seen in stage 15, but no earlier in embryogenesis. Preabsorption with the EPL001 peptide abolished staining (E, F). There was partial co-location of anti-EPL001 antibody staining with that of Repo, a nuclear marker expressed in all glia cells, indicating that only a subset of glial cells were stained by the anti-EPL001 antibody (B, C). There was no co-location with Elav, a nuclear marker expressed in neural cells (D). To further identify the subset of glial cells expressing EPL001, a stain was used for the transcription factor Prospero. It was found that its expression coincides with EPL001 staining (M). Repo staining and anti-EPL001 antibody staining were also present in the embryonic brain in what, by location and morphology, appeared to be the stomatogastric nervous system. To investigate this possibility a stain was deployed to Pointed P1, the marker of a gene influencing stomatogastric glial differentiation. Pointed P1 was expressed within the domain of expression of the anti-EPL001 antibody (P), confirming that the antigen recognised by the anti-EP001 antibody is expressed within the stomatogastric nervous system (De Velasco et al., 2004). To corroborate further, stains were used to Mef-2 (M) and Pericardin, mesodermal and heart markers, respectively. These revealed that EP001 was likely staining the corpus cardiacum, a paired neurohumoral structure related to the stomatogastric nervous system (Hartenstein, Tepass & Gruszynski-Defeo, 1994).

Staining in the fruit fly embryo was largely cytoplasmic. It was not clear if it is also in the cell membrane. If there was staining in the latter, it was not at an augmented level compared with the cytoplasm, as there was not the brighter line at the edge of the cell shown by many membrane antigens (Hartenstein, 1997). Staining in the cytoplasm was uneven, with some regions more heavily staining than others, perhaps suggesting an association with an organelle, the ER being the tentatively favoured candidate.

### Bioactivity

**Figure 5:**
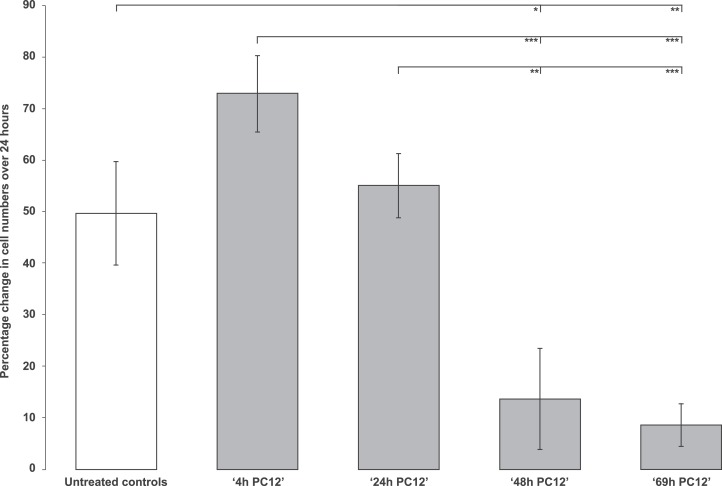
Rat bone marrow cells in culture exposed to PC12-conditioned medium collected at different time points. Data are mean ± SEM; four replicates per group. Student’s *t*-test: * = *P* < 0.05, ** = *P* < 0.005, *** = *P* < 0.001. N.B. Untreated control vs ‘4h PC12’ ns.

**Figure 6:**
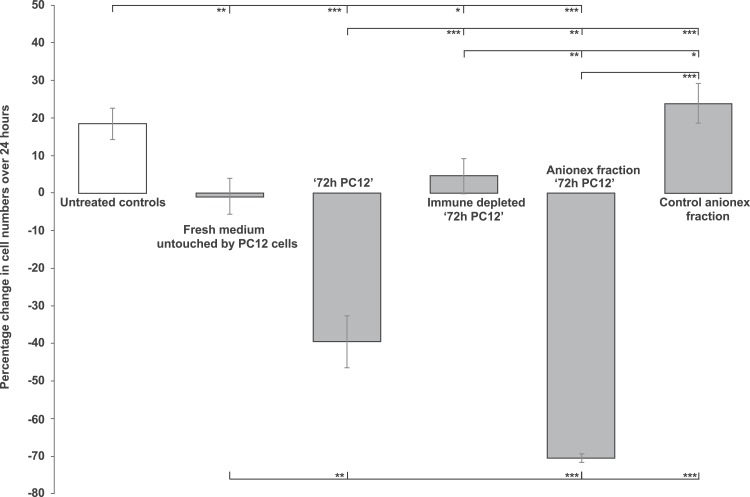
Rat bone marrow cells in culture exposed to PC12-conditioned medium in different forms. The medium was provided neat, immunodepleted (using an anti-EPL001 antibody) and subjected to anion exchange chromatography. Data are mean ± SEM; four replicates per group. Students *t*-test: * = *P* < 0.05, ** = *P* < 0.005, *** = *P* < 0.001.

This involved demonstrating cell-inhibitory activity in biological materials, potentially attributable to the endogenous antigen or antigens of the anti-EPL001 antibodies. Test material selection was on the basis of positive IHC for pheochromocytoma and hypothalamus. Rat pheochromocytoma PC12-cell conditioned culture medium was anti-proliferative in the rat bone marrow cell assay. The effect was enhanced with duration of cell culture, up to 69 h (Fig. 5). Medium at 72 h was more than merely anti-proliferative, with an actual reduction in bone marrow cell numbers in absolute terms (Fig. 6). A round-edged appearance of cells signified ongoing apoptosis, an interpretation supported by other studies in vitro (see ‘Discussion’). The absolute reduction was not seen in 72 h medium subjected to prior immunodepletion using an anti-EPL001 rabbit antibody. An anion exchange fraction of 72 h medium eluting at 0.8 M NaCl showed a greater absolute reduction in bone marrow cells than did whole 72 h medium. PC12 medium untouched by PC12 cells did not support proliferation, though there was no absolute reduction in cell numbers (note that the PC12 medium was different from the medium used for growing bone marrow cells). Anion exchange chromatography removed whatever constraint was present in unconditioned PC12 medium (Fig. 6, ‘Control anionex fraction’).

**Figure 7:**
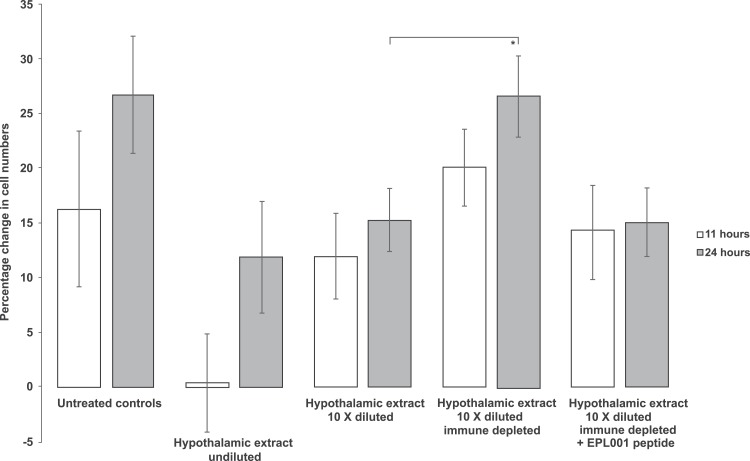
Rat bone marrow cells in culture exposed to rat hypothalamic extract in different forms. The extract was provided neat, diluted and immunodepleted (using an anti-EPL001 antibody, without and with the presence of EPL001 peptide). Data are mean ± SEM; four replicates per group. Students *t*-test: *P = 0.050 (other groups narrowly ns: e.g. untreated controls 24 h vs hypothalamic extract undiluted 24 h, *P* = 0.09; Hypothalamic extract 10× diluted + PP vs the same + EPL001, *P* = 0.06).

Control cells in a subsequent assay increased after 24 h by 49.64% (SEM, ±9.98). Though the proliferation rate varied across this study series, the appearance and behaviour of the untreated control cells was deemed within normal limits, indicating an apparently robust experimental system. This time the ‘72 h PC12’ anion exchange fraction did not cause an absolute decline in cell numbers, merely a blunting in proliferation, to +20.24% (±6.27; *P* = 0.026). Cells exposed to the 0.8 M NaCl anionex fraction subject to prior immune depletion with an anti-EPL001 rabbit antibody returned a value of +37.01% (±7.31; ns), consistent with the removal of an inhibitory influence. A further in vitro assay returned these findings: control numbers after 24 h, +26.21% (±2.53); cells exposed to a sub-30 kDa ultrafiltrate of ‘72 h PC12’ conditioned medium, +6.83% (±1.46, *P* = 0001); cells exposed to the same filtrate subject to immune depletion with an anti-EPL001 rabbit antibody, +18.36% (±4.25, *P* = 0.022 versus test, ns vs controls). This is consistent with an antibody-relevant inhibitory PC12 molecular influence of <30 kDa. Addition of undiluted rat hypothalamic extract had an anti-proliferative effect on bone marrow cell growth, with some round-edged apoptotic cells observed. This inhibitory effect was attenuated when the extract was diluted 10-fold (Fig. 7). Prior immunodepletion with an anti-EPL001 rabbit antibody fully rescued cell growth, except when EPL001 peptide was added as well during the immunodepletion process, achieving preabsorption.

Immune depletion has been demonstrated with the anti-EPL001 goat antibody as well as with the anti-EPL001 rabbit antibody. Studies involved neat serum from sheep and ultrafiltered fractions thereof, prior to a change of feedstock. In a representative study untreated control cells increased in number over 24 h by +25.21% (±2.53). Cells exposed to a sub-10 kDa fraction of sheep ultrafiltered serum returned a growth figure of +13.40 (±6.95), consistent with the presence of an inhibitor. That fraction subject to immune depletion with the anti-EPL001 goat antibody grew by 29.39 (±2.56), consistent with the removal of said inhibitor (*P* < 0.05).

### Molecular weight

**Figure 8:**
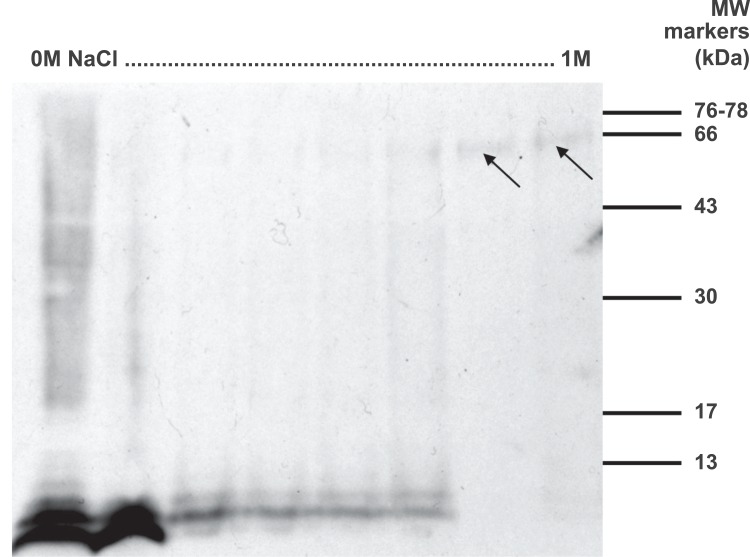
Western blot of anionic exchange chromatography fractions of rat hypothalamus extract. Salt gradient indicated, 0–1 M NaCl. About 10 μl from every fifth fraction (from 50) was run on a 15% SDS PAGE gel in non-reducing conditions and analysed by western blot using the anti-EPL001 goat polyclonal antibody at a dilution of 1/400. Arrows denote broad faint bands of 60–65 kDa in high salt fractions.

What was the likely molecular weight or weights of the endogenous antigen or antigens of the anti-EPL001 antibodies? Sheep serum in bulk subjected to anion exchange chromatography and western blotting produced antigen bands at ∼7+ kDa as follows: three bands that were circumflex shaped (^) in adjacent low salt fractions (∼0.2 M) and a single faint flat band in a high salt fraction (0.8 M). This finding united prior separate observations of active forms coming off in conditions of low salt (Hart, 2003) and high salt (Hart, 2013, Example 7a therein). Nothing more could be learned of these sub-10 kDa bands, however. The use of 2D gels qualified by westerns and analysed by MS also proved fruitless regardless of feedstock, with non-specific hits predominating (e.g. tropomyosin, dynein heavy chain, etc. in rat hypothalamic extract). PC12 conditioned media, as used in the in vitro assay, was analysed on 1D gels. Westerns on the 0.8 M NaCl anion exchange fraction using the anti-EPL001 goat antibody repeatedly yielded a band at ∼7 kDa, but its faintness and proneness during bulking up to depletion under freeze/thawing foiled chemical analysis. A western blot of anion exchange chromatography fractions of rat hypothalamic material showed ∼7+ kDa bands across low and medium salt fractions, which were circumflex shaped as in the sheep and additionally displayed laddering (Fig. 8). Broad faint bands of 60–65 kDa were visible in high salt fractions.

**Figure 9:**
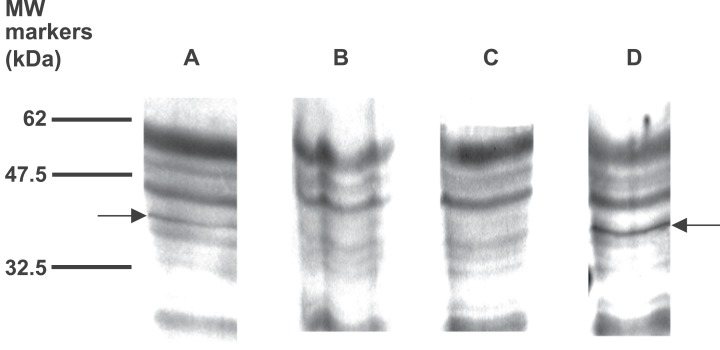
Western blot of aqueous extract of rat hypothalamus, with preabsorption. Samples were analysed by 12% SDS PAGE under non-reducing conditions and then by western blot using the goat polyclonal antibody at a dilution of 1/400 and secondary antibody at 1/5,000. Arrows show a band at 35–45 kDa. (A) Not preabsorbed. (B) Preabsorbed with EPL001. (C) Preabsorbed with KVKEFNNI. (D) Preabsorbed with MKPLTG.

In an earlier phase of the project the use of less pure material had proved helpful in confirming the EPL001 sequence from a discrete band on a crowded gel (Hart, 2013, Fig. 1 therein, densitometric analysis). That preparation had involved sheep ultrafiltered plasma without anion exchange chromatography. Following that approach a western was tried on neat aqueous extract of rat hypothalamic material. Multiple bands were visualised with plenty of background yet only one band, at 35–45 kDa, was eliminated by preabsorption with EPL001 (Fig. 9). The same elimination was achieved by preabsorption with the EPL001 *C*-terminal KVKEFNNI, but not with the *N*-terminal MKPLTG.

**Figure 10:**
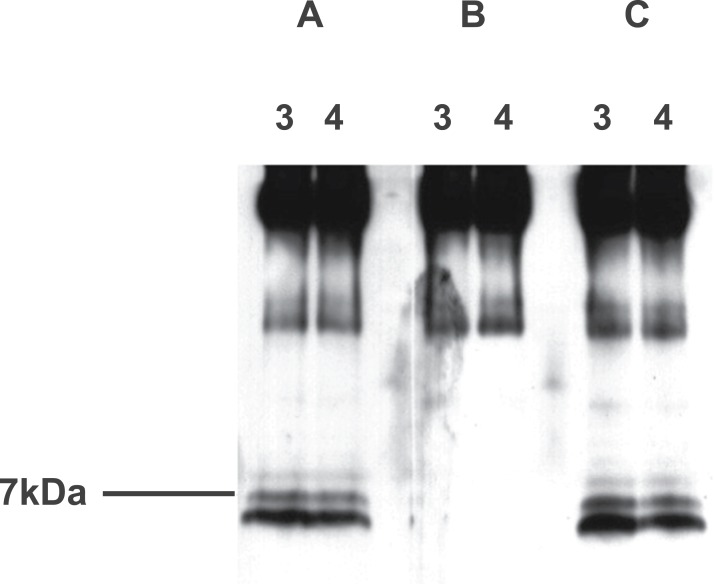
Western blot analysis of aqueous extract of rat hypothalamus purified using an immunoaffinity column, with preabsorption. Samples were analysed using a 10–20% tricine gradient gel under non-reducing conditions and then by western blot using the goat polyclonal antibody and secondary antibody at 1/3,000. Fractions 3 and 4 analysed. (A) Preabsorbed with MKPLTG (2 μl antibody + 25 μl peptide at 100 μM, 1 h. RT). (B) Preabsorbed with KVKEFNNI (as for A). (C) Not preabsorbed.

Prior to the commencement of immunopurification, then, sheep and rat material had both produced western bands at ∼7+ kDa. Additionally, rat hypothalamus had produced others at 35–45 and 60–65 kDa. Immunopurification initially involved the anti-EPL001 goat antibody on affinity columns using rat hypothalamus extract as feedstock. Laddering was seen at ∼7 kDa, in the form of a main band and two minor bands (Fig. 10). These items could be preabsorbed by a peptide based on the *C*-terminus of EPL001 but not by one based on its *N*-terminus. Higher MW bands were not sensitive to preabsortion and were assumed to be Ig. The upper bands could be seen on a gel stained with Coomassie Blue, but the ∼7 kDa bands could not. A rerun of this column purification allowed Edman sequencing of the main ∼7 kDa band. An overlying sequence was kappa light chain. Underlying sequence data yielded weak database hits to fibronectin type III, latrophilin 1, myocilin and a neurexophilin related protein, FAM55D. Only the last-mentioned had any resemblance to the *C*-terminus of EPL001. This led to its being deemed a candidate for the inhibitory hormonal factor (Hart, 2013), as discussed below (see ‘Bioinformatics’).

**Figure 11:**
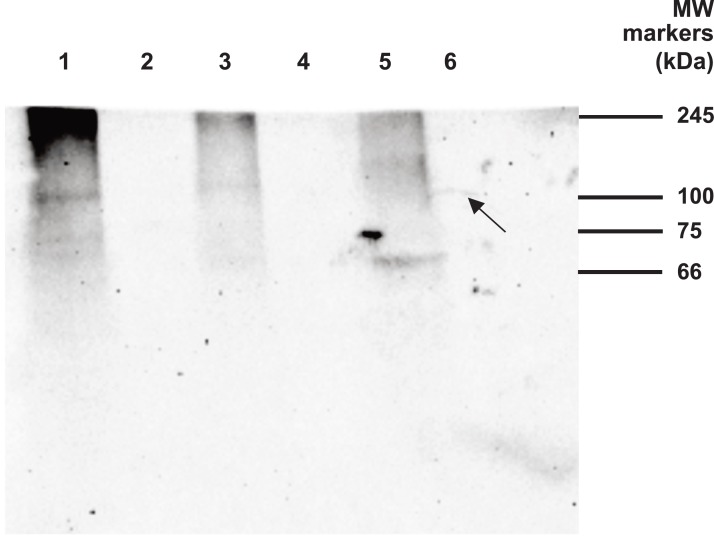
Western blot analysis of *Drosophila* embryo material. Samples were analysed by 12% SDS PAGE under non reducing conditions and then by western blot using the goat polyclonal antibody at a dilution of 1/400 and secondary antibody at 1/5,000. IP involved the same primary antibody. Lanes 1–6. Lane 1: Fruit fly material (unfixed) extracted with 1% NP40. Lane 2: IP of Lane 1 material. Lane 3: Fruit fly material (unfixed) extracted in RIPA buffer. Lane 4: IP of Lane 3 material. Lane 5: Fruit fly material (fixed) extracted in RIPA buffer. Lane 6: IP of Lane 5 material. Arrow denotes the only IP band, which is discernible in fixed material as faint and arched.

During the IP campaign westerns of rat hypothalamic extract, unfixed and fixed with formaldehyde (hereafter ‘formalin’), showed no bands and the same was true for (exclusively unfixed) PC12 material. *Drosophila* embryo material subjected to fixation, RIPA antigen retrieval and IP yielded in western blotting a very faint arched band at ∼100 kDa (Fig. 11, Lane 6). In contrast, fruit fly material that was unfixed yielded no discernible bands after IP, either in the absence of antigen retrieval (Lane 2) or with RIPA antigen retrieval (Lane 4).

### Identification

The goat anti-EPL001 antibody was used in an antigen capture IP protocol to identify the antibody’s endogenous antigen or antigens. Low stringency was applied in the IP/LC–MS campaign—single peptide IDs, 95% confidence, slender epitopes—to provide endogenous antigen candidates for qualification by other means. Mass spectrometric analysis with a FDR filtered at 5% (i.e. 95% confidence level) of fruit fly embryos using *unfixed* tissue (i.e. frozen and not formalin fixed and not subjected to antigen retrieval) yielded a control total of 700 and test total of 1,129 hits, of which latter 526 were test specific, i.e. had a zero control score (unfixed control specifics were 104). Fly embryo tissue subject to formalin fixation (and RIPA antigen retrieval) gave 634 control hits (including 588 control specifics) but just 71 test hits, of which latter 30 were test specific. Epitope mapping (see above, ‘Epitope’) had indicated that the second half of the EPL001 peptide was the likely location of the epitope or epitopes seen by the goat polyclonal antibody used in the IP protocol. On this basis, among the test-specific proteins, a partial epitope was seen in 11 of the 30 (Table 1).

**Table 1:** Fruit fly embryo *fixed* tissue (RIPA antigen retrieval) test-specific MS candidate proteins with partial epitopes.

Fly candidate no.	Accession no.	Name, description, gene	Test score (area)*	Triplet or better epitope match to KVKEFNNI	MW and match to western (∼100 kDa)	Match to fly IHC** protein/RNA expression cellular/timing and tissue intensity localisation	
1	Q9VDH8	Ribosomal protein S30, Isoform A; *CG15697*	1.38E8	KVK	14.6 ✗	✗	✗
2	Q9W2R3	Skittles, PIP Kinase (PIP5K); *CG9985*	3.39E7	NNI	87.7 ✓	✗	✗
3	M9NFR5	Ypsilon schachtel, isoform B; *CG5654*	1.44E7	FNN	37.0 ✗	✗	✗
4	C5WLT4	Metabotropic glutamate receptor; *CG11144*	2.58E6	KEF	59.3 ✗	(✓)	✗
5	P26019	DNA polymerase alpha catalytic subunit; *CG6349*	1.67E6	KEF, NNI	169.8 ✗	✗	✗
6	Q9W2X8	Uncharacterised protein; coiled coil; *CG43902*	8.35E5	KxxxxNNI	136.7 ✓	✓	(✓)
7	Q9NDJ2-2	Isoform B of Helicase domino; *CG9696*	7.73E5	VKE	274.4 ✗	✗	✗
8	M9PGZ6	Zormin, isoform H; ECM, secreted; *CG33484*	7.23E5	VKE	330.8 ✗	✗	✗
9	Q0KHS7	Lipid storage droplet-2; surface-binding protein; *CG9057*	6.22E5	KVK	36.3 ✗	✗	✗
10	O61492-2	Isoform B of flotillin-2; cell adhesion, GPI anchored; *CG32593*	3.65E5	VKE	46.6 ✗	✗	(✓)
11	Q9VGM1	Multidrug resistance protein orthologue; *CG14709*	0.00***	KVK	147.8 ✗	✗	✗

**Notes:**

UniProt database (http://www.uniprot.org/) used for search: *Drosophila melanogaster*.

* E8 = ×10^8^, etc.

** Databases consulted include: (FlyBase, http://www.flybase.org/, EMBL-EBI QuickGo, http://www.ebi.ac.uk/QuickGO/GProtein?ac=Q9W2X8, Genevisible, http://genevisible.com/search).

*** Detected by machine without area assignment.

MW, molecular weight; kDa. Candidate 3 identified on the basis of seven peptides; all others, single peptides. FDR 5%. Filtering at 1% FDR removes all candidates except 1, 2, 3 and 9. MS peptides unexhibited, except that for candidate 6, which was a 23mer comprising residues 51–73, DLQQQRHQQPSEIEDDDGARPDK, coverage 1.89%, score 2.42.

Candidate 6, Q9W2X8, an unnamed uncharacterised predicted protein (http://www.uniprot.org), was identified at 5% FDR on the basis of a single peptide, albeit a 23mer (Table 1 legend), with no other protein possibilities cited by the software. Q9W2X8 can be deduced to be the fly IHC antigen on multiple grounds: (i) its presence in test material and its absence from control material; (ii) its possession of the EPL001 *C*-terminal partial epitope **K**xxxx**NNI** (residues 186–194 of 1,220 aa); (iii) a timing and intensity pattern of late-stage embryonic exonic expression (FlyBase, http://www.flybase.org/) that matches the IHC staining, which latter is only present in embryonic stages 15 and 16; (iv) a description of Q9W2X8 as ‘cellular component’ and ‘cytoplasm’ (EMBL-EBI QuickGo, http://www.ebi.ac.uk/QuickGO/GProtein?ac=Q9W2X8) (see ‘Bioinformatics’), which coheres with the observed intracellular histology, and ‘embryo’ (Genevisible, http://genevisible.com/search); (v) an order-of-magnitude abundance after purification that aligns better with the IHC staining intensity and western band intensity (Fig. 11) than would be the case if its abundance were that of a higher-order-of-magnitude comparator with the **NNI** motif such as Fly Candidate 2 (FlyExpress, http://www.flyexpress.net/); and (vi) a rough concordance on MW of 136.7 kDa with that expected from the western of ∼100 kDa, given a wide margin of error on the MW markers for larger proteins, as a gel with a high % acrylamide had been used suitable for smaller proteins. Of the 11 *fixed* candidates none was seen in *unfixed* fly test material. One however was seen in *unfixed* fly control material: Fly Candidate 3 (M9NFR5, Ypsilon schachtel, isoform B). As a heuristic the fly observations direct the eye in the mammalian analysis towards fixed material.

*Unfixed* material from the mammalian campaign at 5% FDR yielded control hits in huge numbers: PC12, 1,095 (including 191 control specifics); hypothalamus, 1,634 (379 control specifics). The overlap (‘unfixed control overlappers’) between these two groups was 558 proteins. Detailed analysis was not attempted on what is a vastly miscellaneous control assemblage. *Unfixed* material from the mammalian campaign at 5% FDR gave these test results: PC12, 1,516 (test specific, 603); hypothalamus, 1,681 (test specific, 424). Of the two groups of test specifics, 34 proteins were present in both (‘unfixed test overlappers’), with 11 having triplet partial epitopes. The (unpromising) character of these 11 is as follows: enzymes, 4; nuclear proteins, 3; cytoskeletal proteins, 2; ribosomal protein, 1; membrane protein, 1. *Fixed* hypothalamic material at 5% FDR gave more restrained control results than unfixed tissue: citrate antigen retrieval, 438 (114 control specifics); RIPA antigen retrieval, 226 (50 control specifics). The cleaner preparation obtainable with RIPA provided the rationale for using this method of antigen retrieval in the fruit fly IP. There were 163 proteins in both groups (‘unfixed control overlappers’), including keratin, tubulin, actin, enzymes, etc. *Fixed* results for test hypothalamic material at 5% FDR were as follows: citrate antigen retrieval, 454 hits (114 test specifics); RIPA antigen retrieval, 406 hits (213 test specifics). The overlap of the two lists of test specifics (‘fixed test overlappers’) is 11. None of these is present among *unfixed* hypothalamus test specifics. Of the 11, 7 have a partial epitope (Table 2, rat candidates 1–7). The overlap of the fixed test overlappers possessing triplet partial epitopes with proteins having the same among the unfixed test overlappers (i.e. PC12 and hypothalamus together) is zero.

**Table 2:** Rat hypothalamus *fixed* tissue test-specific MS candidate proteins (selected from 49) with partial epitopes.

Rat candidate no.	Accession no.	Name, description, gene	Citrate test score (area*)	RIPA test score (area*)	Triplet or better epitope match to KVKEFNNI	MW and match to westerns (∼7+, 35–45 or 60–65 kDa)	Identity (aa %) with fly candidate 6, Q9W2X8
1	F1M5Z5	Anoctamin; multi-pass membrane protein; ion channels; clotting; *Ano6*	4.25E7	5.87E6	GxxxxxNNI, PLxxxxxxxNNI	92.2 ✗	0.3
2	D3Z822	Protein Atr; kinase; *Atr*	7.45E6	3.40E6	KEF, EFN	301.0 ✗	1.3
3	D3ZZJ4	Protein Ttc21a; cilium protein; *Ttc21a*	4.10E6	1.03E6	VKExN, LxxxVKE, KxxNNI	148.6 ✗	13.8
4	Q8CGL8	Secretogranin II variant (fragment); neuroendocrine secreted protein; *Scg2*	3.09E6	2.52E6	VxxxNNI	37.1✓	5.2
5	P63170	Dynein light chain 1; motor protein; *Dynll1* (also in hypothalamus *unfixed* control and PC12 *unfixed* test and control)	1.98E6	6.73E6	KEF	10.4 ?	1.5
6	P85972	Vinculin; membrane-cytoskeletal protein, actin binding; *Vcl* (Also in PC12 *unfixed* control)	8.42E5	1.32E6	VKE (×2)	116.5 ✗	7.9
7	D3ZBB8	Cytoplasmic dynein 2 heavy chain 1; motor protein; *Dync2h1*	0**	5.95E7	VKE (×2), NNI	491.0 ✗	5.2
8	D4AAG8	Protein Smchd1; ATP binding, nuclear; *Smchd1*	8.97E6	–	KVK, VKE, VxxFNNI	225.9 ✗	5.1
9	F1M1M4	Protein Dst (fragment); dystonin, adhesion junction protein; deleted from UniProt database	6.31E6	–	KVKE, NNI	300.0 ✗	9.2
10	F1M7V4	Protein piccolo (fragment); synaptic vesicles; *Pclo*	–	1.31E8	KVK, MxxxxxxxKEF	426.1 ✗	6.8
11	F1M2I5	Opioid-binding protein/cell adhesion molecule; Opcml	–	5.76E6	PxxxKVKxxxN	22.5 ✗	4.8
12	Q4V8E7	Protein Tex21; ‘Testis expressed gene 21,’ coiled coil; *Tex21*	–	3.36E6	KVKxxxN, VKE (×2)	60.3 ✓	10.9

**Notes:**

UniProt database (http://www.uniprot.org/) used for search: *Rattus norvegicus*.

* E7 = ×10^7^, etc.

** Detected by machine without area assignment.

MW, molecular weight, kDa. Amino acid identity (%) established via EMBOSS Needle (http://www.ebi.ac.uk/Tools/psa/emboss_needle/). The first seven candidates were all the test specifics found in both citrate and RIPA antigen retrieved samples. The rest are a selection from a further 42 test specifics found in one sample only having above-triplet partial epitopes. Candidates were identified on the basis of single peptides, except candidate 5 (two peptides in both samples) and candidate 7 (1 in citrate, 2 in RIPA). FDR 5%. Filtering at 1% FDR removes over half of the above candidates leaving 5, 6, 8, 9 and 11. MS peptides unexhibited, except that for candidate 4, a 7mer in both samples comprising residues 11–17, IILEALR (coverage 2.17%), scores 1.71/1.98 (citrate/RIPA).

The non-overlapping fixed hits (citrate, 114 − 11 = 103; RIPA, 213 − 11 = 202) were subject to a sift in the interests of data manageability. Excluded were enzymes, housekeeping proteins, ribosomal proteins, exclusively nuclear proteins, structural cytoskeleton proteins and items barren of interest after inquiry. The remainder were searched for triplet partial epitopes, with the result that 26 citrate-only and 16 RIPA-only candidates were added to the seven fixed overlappers to give 49 hypothalamic candidates in all. Among these the top 3 citrate-only hits were Uncharacterized protein (fragment) (M0R5G3, VKE), Protein Jakmip2 (D3ZQE7, VKE) and Protein Larp1 (fragment) (F1M062, VKE and NNI); while the top 3 RIPA-specific hits were Protein piccolo (fragment) (F1M7V4, KVK and KEF), Contactin-associated protein 1 (P97846, EFN and FNN) and selenium-binding protein (F1LRJ9, KVK). Five of the non-overlappers with ‘better than triplet’ epitopes are included in Table 2 (rat candidates 8–12). None of these appear anywhere in the *unfixed* hypothalamic data, control or test.

No western blot MW readout was obtained from rat hypothalamus IP material. In the broader western blot campaign on rat hypothalamus three MWs emerged: ∼7+, 35–45 and 60–65 kDa. The molecular weights of the 49 mammalian candidates range from ∼10 to ∼500 kDa. The only one within range of the low MW figure is the uninteresting dynein light chain, at 10.4 kDa (Table 2, candidate 5). Six of the 49ers fall within the 35–45 kDa MW range or are close to it: (i) signal sequence receptor, alpha (Q4V7D1, epitope match: NNI, MW 32.2 kDa); (ii) annexin (fragment) (O70371, KEF, 33.9); (iii) protein S1pr3 (F1M9D3, KVKE, 42.3); (iv) SgII variant (fragment) (Q8CGL8, NNI, 37.1; Table 2, candidate 4); (v) armadillo repeat-containing X-linked protein 3 (Q5XID7, KEF, 42.5); and (vi) radixin isoform (T1SRT4, VKE, 45.3). The only one of these in both citrate and RIPA retrieved samples is the SgII variant. Three of the 49ers fall within the 60–65 kDa MW range or are close to it: (i) leucine-rich repeat transmembrane neuronal protein 2 (fragment) (M0RC63, VKE, 58.6); (ii) protein Tex21 (Q4V8E7, KVKxxxN, VKE ×2, 60.3, Table 2, candidate 12); and (iii) prosaposin (Q6P7A4, VKE, 61.0). None of these appeared in both citrate and RIPA retrieved samples. An EMBOSS Needle (http://www.ebi.ac.uk/Tools/psa/emboss_needle/) comparison of the lead fly candidate Q9W2X8 and 49 rat candidates showed that 40 had aa identities in single figure percentages, including the SgII variant, at 5.2% (Table 2, candidate 4). The remaining nine were scattered up to a maximum of just below 15%, which is insufficient to support a claim of relatedness. The IP campaign, fly as well as rat, provided few proteins with more than a triplet partial epitope match to the EPL001 sequence. Of the 12 aa triplets in the 14mer EPL001, the first, MKP, is present in 3% of *D. melanogaster* and *R. norvegicus* proteins in Swissprot. The next 7 (KPL, PLT, LTG, TGK, GKV, KVK and VKE) are present in 10–15% of proteins in both species, while the final four (KEF, EFN, FNN and NNI) are present in 5–9% of fly proteins and 3–7% of rat proteins. These proportions were broadly upheld in the data, with Pearson’s correlation coefficient *r* ≥ 0.9 for fly and rat test-specific triplet incidence versus species-specific Swissprot databases. This indicates that IP was not skewing the population of proteins generically in the direction of EPL001 triplets. A fivefold hit (5/14, 36%), **P**xxx**KVK**xxx**N**, is provided by Rat Candidate 11, ‘Opioid binding protein/cell adhesion molecule,’ F1M2I5 (Table 2). This was present in RIPA retrieved hypothalamic material only and at 22 kDa is a non-match to any of the three western MWs. The human version is Q14982, gene *OPCML*, with exclusively CNS expression. ‘The encoded protein is localized in the plasma membrane and may have an accessory role in opioid receptor function’ (Human Protein Atlas). More promising is rat candidate 4 (Table 2), ‘SgII variant (Fragment),’ Q8CGL8 (hereafter ‘SgIIvar’). This is a member of the granin acidic soluble protein family found in intracellular secretory granules in endocrine, neuroendocrine and neural tissues. It is the sole secreted entity among the seven fixed test overlappers, having the partial epitope **V**xxx**NNI** (residues 131–137 of 322 aa). Note that NNI is also seen in the partial epitope of the fly lead candidate Q9W2X8. SgIIvar was identified in both citrate and RIPA samples at 5% FDR on the basis of the same single 7mer peptide (Table 2, notes). At ∼37 kDa rSgIIvar corresponds to the medium western blot hypothalamic MW, in the context of which EPL001 preabsorption was demonstrated. The MS program identified SgIIvar as the Master Protein for both citrate and RIPA samples, but in each MS protein group and sharing the identification at a lower percent coverage, because they are larger proteins, are G3V7X2 (‘Secretogranin 2, isoform,’ 579 aa) and P10362 (‘Secretogranin-2,’ 619 aa). Both of these SgIIs have the **V**xxx**NNI** partial epitope and are products of the same gene as SgIIvar. Rat candidate 4 should therefore properly be described not as SgIIvar but as ‘an rSgII proteoform.’ P10362 is outside the high westerns MW range of 60–65, at 71 kDa (∼68 after its signal peptide is cleaved off), but G3V7X2 is not: 66.6 kDa (∼63 without its signal peptide). So there is the interesting situation where two of the three westerns MWs can be accommodated by SgII proteoforms. P10362 is not picked as a Master Protein anywhere in the full dataset but G3V7X2 is, in three of the four categories of MS data relating to *unfixed* mammalian material: (i) PC12 unfixed control (five peptides, including IILEALR, the identification peptide of SgIIvar, coverage 8.24%, score 8.71); (ii) hypothalamic unfixed control (six peptides, coverage 11.79%, score 25.85); and (iii) hypothalamic unfixed test (eight peptides, coverage 15.19%, score 25.94). Because of the peptides identified none of these three could be SgIIvar but all could be P10362, which is deprived of priority on the basis of its being a bigger protein, yielding a lower percentage coverage from the identified peptides, while earning equal scores. There is only one gene for SgII in the rat, which is designated *Scg2*. This has three exons and is found on chromosome 9 (RGD: Rat Genome Database, http://rgd.mcw.edu/, UCSC: UCSC Genome Browser, https://genome.ucsc.edu/index.html). At 619 aa P10362 is the full-length SgII encoded by this gene. G3V7X2 is a shorter splice variant, at 579 aa, having a 40-residue deletion after position 317 compared with P10362 (in Uniprot, http://www.uniprot.org) and there is also an A instead of T at position 2 in G3V7X2). If G3V7X2 is P10362 with a central pleat, SgIIvar (Q8CGL8), a further splice variant (NCBI: NCBI Genbank, www.ncbi.nlm.nih.gov/genbank/) is G3V7X2 with both ends snipped off: 104 of G3V7X2’s *N*-terminal residues are absent from SgIIvar and 153 *C*-terminally, leaving the central 322 aa (there are also two variant residues: at P10362/G3V7X2 position 160 SgIIvar has a K for an E, while at G3V7X2 position 330, which is P10362 position 370, there is a T for an M). The aa identity between P10362 and G3V7X2 is 93.4%; between G3V7X2 and SgIIvar 55.3%; and between P10362 and SgIIvar 51.5% (EMBOSS Needle, http://www.ebi.ac.uk/Tools/psa/emboss_needle/). SgII at full length in the rat is P10362, a prohormone. It is the progenitor of three small peptides by endoproteolytic processing at dibasic aa cleavage sites (comprising Ks and Rs or one of each): (a) secretoneurin (SN, 33 aa, residues 184–216 of the proprotein, which includes a 30 aa signal sequence); (b) EM66 (66 aa, residues 219–284; so named for an *N*-terminal glutamic acid and a *C*-terminal methionine); and (c) manserin (40 aa, residues 529–568). The SN and EM66 sequences are to be found within G3V7X2 and Q8CGL8 as well, with EM66 possessing the partial epitope **V**xxx**NNI**. It lacks however the SgIIvar MS identification peptide, which in both P10362 and G3V7X2 is at residues 115–121. G3V7X2 is not recorded in UniProt (http://www.uniprot.org) as being processed into peptides and neither is SgIIvar.

Protein identification on the basis of a single MS peptide, as here (at 95% confidence), even when doubly achieved via different antigen retrieval systems, must be verified by complementary means (Taylor & Goodlett, 2005). Potential correspondence to the top two of the three hypothalamic western blot MWs has already been noted, with successful EPL001 preabsorption in regard to the middle and lower values. In terms of other identification criteria for the sought-for inhibitory factor, the candidate must co-localise in IHC with CgA in neuroendocrine cells in disparate tissues and, inter alia, it must appear as axonal beading in the median eminence of the sheep hypothalamus, as well as in pyramidal neurons in the rat cerebral cortex. It must also be present as an anti-proliferative antigen in the PC12 secretome and thus be potentially subject to immunoneutralisation therein. The only protein likely to fulfil these criteria among the 49 partial-epitope-bearing formalin-fixed antigen-retrieved rat hypothalamus candidates is the rSgII proteoform.

### Bioinformatics

This was an attempt to determine in silico if the EPL001 amino acid sequence is meaningful in terms of any known protein and, if so, if that protein is likely to be relevant in the context of a tissue-mass inhibiting hormonal influence. The EPL001 aa sequence MKPLTGKVKEFNNI was obtained from ovine blood plasma as a result of Edman *N*-terminal sequencing. Anti-EPL001 antibodies have been used in expression library screens, as well as in the antigen capture studies described here, and oligonucleotide mixtures representing the EPL001 amino acid sequence have been used in PCR-based gene discovery studies (Hart, 2013), but without reproducible outcomes. BLAST searches on the 14mer sequence have revealed no *N*-terminal matches in the top 100 hits of the full protein public database, the metazoan top 100 or the mammalian top 100. Referring then to a potential match to the internal part of a protein, at the top of the database of the sheep (*Ovis aries*) are toll-like receptor 2 hits: **PLTGKVK** (7/14, 50%; e.g. CAJ65834). Next are cullin-associated proteins. A mid-molecule resemblance is provided by a human protein related to neurexophilin, NXPE family member 1 (Q8N323): **M**SS**P**A**LT**AGAS**GKV**MD**FNN**G. Formerly known as Fam55A, this entity is secreted and has relevant activity, but only the six residues that match *C*-terminally are in register (6/14, 43%). Note, though, that a splice variant of neurexophilin does indeed commence at the methionine residue, making this sequence *N*-terminal. A related protein in the rat is NXPE family member 4, formerly FAM55D (Q5XI89). This emerged as a candidate (Hart, 2013) from a column immunopurification exercise (see above, Molecular weight). The overlap between this protein and the Edman data (after subtracting kappa light chain sequence) was VxxxNNG, while the full alignment with EPL001 is **M**SS**P**A**L**KAGAS**GKV**TD**FNN**G. An ovine equivalent (X_012010718.1) is an even weaker hit: IFS**P**A**L**KAGAS**GKV**TD**FNN**G (in register, 6/14, 43%, as for the rat). The top BLAST hit in the full database is Bsm1 (AAL86024.1), the commercially available restriction enzyme from an exotic thermophilic bacterium, *Geobacillus stearothermophilus*: E**K**S**L**S**GKVKEFNNI** (11/14, 79%). The next hit is to a hypothetical protein (WP_029981464.1) in marine cyanobacteria, *Prochlorococcus* sp.: **GKVKEFNNI** (9/14, 64%). Below this are many dubious partial gap-hits to proteins of *Staphylococcus* sp. and other bacteria. The implausibility of these notwithstanding, the possibility remains that these hits may betoken a bacterial provenance for the EPL001 sequence, although this would not be consonant with the mammalian neuroendocrine IHC staining achieved with the anti-EPL001 antibody. Particular confidence is reposed in four residues in the EPL001 sequence, which on one occasion were all that could be obtained from Edman sequencing: - -P- - - -V- -FN- -. Hits lacking the prominent P in particular, such as the bacterial ones, can be discounted. The indecisiveness of the bioinformatics analysis provided the justification for the IP/LC–MS campaign described herein.

There are 11 MS candidates for the fruit fly antigen (Table 1) and 49 for the rat antigen (Table 2, selected list of 12). All of these 60 were test specific for formalin-fixed tissue and have partial epitopes. Aligning the 11 against the 49 using BLASTp, with compositional matrix adjustment (Altschul et al., 2005) and permissive parameters, yielded 24 alignments between fly candidates 1, 2, 3, 4, 6 (lead candidate), 8 and 9 and a total of seven rat candidates, excluding no. 4 (lead candidate). These alignments are weak, however. In four cases there is a partial epitope triplet in either the fly or rat protein, but without an answering match in the other sequence. The lead candidate for the fruit fly IHC antigen is fly candidate 6, an unnamed entity with accession number Q9W2X8. UniProt (http://www.uniprot.org) gives no function for this protein and a minimal annotation score, for what is a predicted protein, yet the possible importance of Q9W2X8 is indicated by the record in FlyBase (http://www.flybase.org/) which states ‘The phenotypic class of alleles includes: “partially lethal—majority die.”’ Otherwise FlyBase reports that there is no information supporting a molecular function or biological process for the relevant gene. A verdict for Q9W2X8 of ‘None predicted’ is available for protein family membership, domains and repeats, biological process, molecular function and cellular component (EMBL-EBI InterPro, http://www.ebi.ac.uk/interpro/protein/Q9W2X8). Elsewhere in the databases Q9W2X8 is described as a cytoplasmic cellular component whose function is ‘regulation of apoptotic process’ and ‘negative regulation of mRNA splicing, via spliceosome’ (EMBL-EBI QuickGo, http://www.ebi.ac.uk/QuickGO/GProtein?ac=Q9W2X8). Q9W2X8 is classified (PANTHER, http://www.pantherdb.org/) in ‘PANTHER Family: SWI/SNF-related matrix-associated actin-dependent regulator of chromatin subfamily-related (PTHR10799)’ and ‘PANTHER Subfamily: Apoptotic chromatin condensation inducer in the nucleus (PTHR10799:SF637).’ *Drosophila* orthologues in this database include Q9VWZ8 (activity undefined), P09956 (‘Regulatory protein zeste’), Q9GQN5 (‘Transcriptional regulator ATRX homolog’) and A8DYV5 (‘Ncoa6, isoform F’), though none of these emerge in a BLAST search as close relatives. The most nearly equivalent genes in the rat and human in the PANTHER database are E9PST5 and Q9UKV3, respectively, both products of *Acin1* genes, where ‘Acin’ unpacks as ‘Apoptotic chromatin condensation inducer in the nucleus.’ These two proteins have a 16.8% and a 17.7% aa identity with Q9W2X8, respectively (EMBOSS Needle, http://www.ebi.ac.uk/Tools/psa/emboss_needle/). In UniProt (http://www.uniprot.org) the activity ascribed to E9PST5 includes ‘negative regulation of mRNA splicing, via spliceosome’ and ‘positive regulation of apoptotic process.’ UniProt describes Q9UKV3 as an auxiliary component in a protein machine responsible for mRNA splicing. Neither E9PST5 or Q9UKV3 come up in BLAST searches on Q9W2X8 against the mammalian database, which yields no hits. Perhaps a fair deduction from the foregoing is that Q9W2X8 is ‘inhibitory.’

Comparing the lead fly candidate Q9W2X8 with rSgII (P10362) indicates 9.8% aa identity (5.2% with the half-sized rSgIIvar) (EMBOSS Needle, http://www.ebi.ac.uk/Tools/psa/emboss_needle/). In the rSgII proteoforms, as in Q9W2X8 and the other fly candidates, the partial epitope is not near the *N*-terminus and this is true for the other 48 rat candidates as well. This is inconsistent with the purportedly *N*-terminal EPL001 sequence, although that was ovine derived. BLAST searches of the 12 rat proteins of Table 2 shows that the partial epitope in 10 is the same in the sheep equivalent. One exception is Rat Candidate 10, piccolo (F1M7V4), where the partial epitopes **KVK** and **M**xxxxxxx**KEF** become three in the sheep homologue W5Q2P0, with the addition of **K**xxxx**KVKE**. The other exception is rSgII itself, where a match of **V**xxx**NNI** becomes **NNI** in W5QEU8, the sheep homologue (sSgII). No homologue to the 322 aa rSgIIvar (Q8CGL8) splice variant is recognised in the sheep proteome. The secondary structure prediction for the 619-residue rSgII is 42% α-helix and 57% coil, with the slightly shorter sSgII (W5QEU8, 613 aa) on 45/54 (RaptorX, http://raptorx.uchicago.edu/). Granin coiled coil structures may play a role in granule core condensation (Bartolomucci et al., 2011). Q9W2X8, itself a coiled coil protein, is predicted to be similar at 38% α-helix and 59% coil (RaptorX, http://raptorx.uchicago.edu/). Secretogranin II proteins have no cysteines, while Q9W2X8 has one. Neither therefore can have the disulphide bonds usually associated with extracellular items, yet the former are certainly the progenitors of secreted bioactive peptides. In this regard rSgII has 9 dibasic sites (R & K) for proteolytic cleavage (Taupenot, Harper & O’Connor, 2003), sSgII 8. Q9W2X8, at twice the number of aa residues overall, has 21 dibasic sites. The SgIIs and Q9W2X8 are acidic. For Q9W2X8 the ratio of negatively charged residues (D & E) to positively charged ones (R & K) is 1.2, with the former comprising 16% of total aa, giving the molecule a calculated pI of 5.9 (Expasy ProtParam, http://web.expasy.org/protparam/). For both sSgII and rSgII the figures are >1.5, 19.4% and 4.7. E is particularly prevalent, representing 12.7% of total residues in sSgII for example, which is two standard deviations from the 20-aa expected mean of 5%. In the fly protein E represents 8.5% of total residues. For sSgII the net charge distribution, i.e. (K + R) – (D + E), is as follows, taking 60 residues at a time (with a final group sub-60): −2, −5, −2, −9, −11, +2, −10, +1, −6, −2, +1. The picture is of overall negativity with negatives clustering centrally (notably bands 4, 5 and 7). The trend line rises left to right from negativity towards positivity. For Q9W2X8 the corresponding figures are: +8, −19, −15, +6, −7, −1, −15, +8, −7, −5, +7, +3, −1, +6, −7, −2, +2, −4, +4, +4, −1. Again negativity prevails, but with positive and negative banding. There is especially strong negative clustering in Bands 2, 3 and 7, with the trend line rising once more from left to right from negativity to positivity.

The *N*-terminal 30 aa of rSgII and Q9W2X8 disclose no homology: 16.7% aa identity, three gaps (EMBOSS Needle, http://www.ebi.ac.uk/Tools/psa/emboss_needle/). The *N*-terminus of rSgII is recognised as a signal peptide that also rates highly as a transmembrane helix (TMH) (CBS, http://www.cbs.dtu.dk/services/SignalP/, http://www.cbs.dtu.dk/services/TMHMM/). Q9W2X8’s *N*-terminus also rates highly as a TMH but not as a signal sequence (score *D* = 4, cutoff 5), although it has two adjacent candidate cleavage sites. The non-identification might be a SignalP program deficiency, however, as recognised *Drosophila* signal peptides also did not register the requisite score in the authors’ hands. Near the signal peptide in rSgII is a sorting domain that directs the protein into the regulated secretory pathway (Courel et al., 2008). This sufficient independent α-helix domain, absent from the shorter rSgIIvar, comprises 17 aa and represents rSgII residues 55–77, commencing MIRA. EMBOSS Needle (http://www.ebi.ac.uk/Tools/psa/emboss_needle/) equates this with Q9W2X8’s residues 42–58, with 35.3% aa identity over the stipulated length, 58.8% similarity and no gaps. In particular the two regions conclude with **L**x**QQ**x**H**x. This motif is at the start of the peptide which allowed Q9W2X8 to be identified by MS, a 23mer representing residues 51–73 (Table 1 legend). The MS identification peptide for the rat SgII proteoform was IILEALR. In rSgII this represents residues 115–121. EMBOSS Needle identifies this in Q9W2X8 with a 7mer in a similar position in the sequence: _155_**IILE**SQ**R**_161_ (identity 71.4%, similarity 85.7%, no gaps). The rSgII and Q9W2X8 sequences at NNI each have a single other residue corresponding to EPL001, as has been noted, but besides NNI the two sequences have only a stray K in common with each other (shaded) that is out of register with the Ks in EPL001, counting back from NNI in that synthetic peptide:

rSgII     _220_RVDEEQKLYTDDEDD**V**YKT**NNI**AYEDVVGGEDWSPMEEKI_259_

Q9W2X8  _172_DAKKYQNSNNKKRS**K**EQKK**NNI**SHHNYKLKNNKENNHHRLA_212_

Apart from the shared NN doublet of NNI, the 40-residue runs above both feature a series of doublets unique to themselves. rSgII (like sSgII) has 14 negatively charged residues (Es and Ds in regularly spaced groups) and four positives (Ks and an R as singletons). In contrast Q9W2X8 has three negative singletons and 12 positives (plus 4 Hs). In rSgII (also sSgII) the negatives (n) are flanked by hydrophobic residues (of which there are five singletons in Q9W2X8):

rSgII  _220_xVnnnxxLYxnnnnnVYxxxxIAYnnVVGGnnWxPMnnxI_259_

For both 40mer sequences an α-helix is predicted to precede the NNI motif (PSIPRED, http://bioinf.cs.ucl.ac.uk/psipred/). Sorting domain relevance can be suspected. There is no consensus sequence for secretory granule sorting signals but the defining features are reckoned to be presence of residues that are charged (either positive or negative) spatially segregated from a hydrophobic patch in α-helices (Dikeakos et al., 2007). *C*-terminal of the NNI landmark is the second main recognised sorting domain in SgII, which in most species is 15aa (Courel et al., 2008). This is predicted to start with a 7-residue α-helix and has a cross-species homology gap after residue 12. In the rat the second sorting domain is _364_(PE)DLIEMLKAGEKPNGL_380_. The prior two residues, PE, are found in all species. PN in the rat is PV in the sheep and cow. The final L in the rat is an S in other species, e.g. human. Switching V for rat N and S for rat L and adding the preceding PE to provide a composite mammalian sequence finds this match in Q9W2X8: _1038_**PE**T**L**-**EM**-**KAG**K-**PVGS**_1063_ (identity 46.2%, similarity 53.8%, three gaps of four, one and four aa). The rat (and mouse) motif KAGE is matched in the fly by KAGK. This tetramer is also present in the fly homologue of the first mammalian sorting and nowhere else in the fly protein (KAG does not appear in the first sorting domain of any mammal, where the corresponding motif is IEN/K). The match for the two fly candidate sorting domains is in fact this: **A**x**KAGK**xxxx**Q**. The KAGK motif in the first fly sorting domain exactly precedes the fly 23mer MS ID peptide. *C*-terminal of the mammalian second sorting domain is a 10mer conserved granin motif (Gerdes et al., 1989) which in rSgII is _497_DNLNDKDQEL_506_. EMBOSS needle finds the following sequence in Q9W2X8: _360_**DNL**-**D**N**D**EI**L**_368_ (identity 60%, similarity 70%, one gap). This match, unlike the others discussed here, is out of alignment with that in rSgII, being *N*-terminal of the second sorting domain.

## Discussion

This paper is not an attempt to close the loop on the inhibitory hormone hypothesis (Hart, 2014) by identifying an endogenous factor and then deploying it to show the requisite tissue-mass reduction. That stage of the investigation has not yet been reached. Instead this is an interim report addressing a problematic candidacy. It asks, Is the ovine-associated polypeptide with the EPL001 amino acid sequence that emerged from the mammalian factor hunt potentially meaningful and relevant, in spite of bioinformatic obscurity? The answer is Yes.

A polypeptide candidate of 7–8 kDa and dubbed Circa-70 on account of its likely residue number turns up repeatedly in the course of a hormone purification campaign, the scale-up feedstock for which is a 3–30 kDa ultrafiltrate of sheep jugular vein plasma. The sequence information that can be obtained from Circa-70 is an Edman-derived 14mer *N*-terminus relating to nothing obvious in the databases and resistant to molecular biological elucidation. The range of possibilities include the consideration that the polypeptide candidate is a miscellaneous mishmash of irrelevance through to its being a postulated hormone responsible as the body’s brake for down-regulating internal organ masses and restricting reproduction before puberty (Hart, 2014). A synthetic form of the 14mer *N*-terminus, the EPL001 peptide, even shows effects in vivo both tissue-reducing (Haylor et al., 2009) and reproductive (Davies & Hart, 2008). In experiments EPL001 inhibits the proliferation in vitro of MCF7 breast cancer cells stimulated by IGF1 and causes a dose-dependent reduction in LH secretion by ovine pituitary cells in vitro and likewise reduces prolactin production (Hart, 2013). It also proves possible to modulate reproduction and lifespan in nematodes using EPL001, with fluorescently labelled EPL001 accumulating preferentially in ovarian tissue (Davies & Hart, 2008). Antisera are raised against EPL001. They are used to generate IHC images in mammals of seeming endocrine relevance: focal neuroendocrine cell staining in disparate tissues, axonal beading in the ovine median eminence (part of the basal hypothalamus), pyramidal neuron staining in the rat cerebral cortex, germinal epithelial staining in the human testes and increased staining in the human adrenal tumour pheochromocytoma. The IHC results swing the research focus from the (moderate staining) ovaries as a site of factor production to the hypothalamus and a pheochromocytoma (PC12) cell line. There are theoretical reasons for interrogating the hypothalamus in particular, in the context of body-wide hormonal controls on internal organ masses via a hypothalamic ‘organostat’ (Hart, 2014). Arresting images from mammalian IHC—including neurons in the arcuate nucleus and other parts of the hypothalamus (see Supplementary IHC Images)—seemingly validate the use of an anti-EPL001 antibody in ligand purification and uphold the non-artefactuality of immunoneutralisation data in vivo and in vitro.

In regard to activity in vitro an anti-proliferative effect is described here on rat bone marrow cells in culture exposed separately to (a) cell culture medium conditioned by prior exposure to rat PC12 adrenal tumour (pheochromocytoma) cells, (b) an aqueous extract of rat hypothalamus and (c) sheep serum in the form of a sub-10 kDa ultrafiltrate fraction. The anti-proliferative effect was reduced in all these cases by prior Protein G bead immunodepletion using an antibody raised against the synthetic 14mer peptide EPL001, suggesting that it is due to one or more endogenous protein ligands (referred to in the singular in the present report for parsimony) sharing an epitope or epitopes (ditto) with EPL001. Immunoneutralisation has been observed (d) in vivo when the administration of an anti-EPL001 antibody caused an overshoot in the compensatory renal growth that follows unilateral nephrectomy in the rat (Haylor et al., 2009), an exaggerative effect consistent with the reduction of an endogenous inhibitor. Furthermore (e) a 3–30 kDa fraction of sheep plasma subjected to prior immunoaffinity purification caused more cell proliferation than a fraction not so treated in a breast tumour assay in vitro involving MDA-MB231 cells (Hart, 2013). This and (c) above suggest that the aforementioned ligand circulates in the blood. These antibody-related counter-inhibitory effects were achieved using a rabbit polyclonal anti-EPL001 antibody (a, b, e), a goat antibody (c) and a mouse monoclonal antibody (d), in three different laboratories using rat and sheep source material. The immunodepletion in (b) was not seen when the anti-EPL001 antibody was preabsorbed with EPL001 (Fig. 7).

Beyond an anti-proliferative effect there was apparent apoptosis. This is in line with the effect in vitro of sheep ultrafiltered plasma on PC3 and MCF7 cancer cells, with reduction in cell viability assessed by Alamar Blue and apoptosis confirmed by Annexin V and flow cytometry (Hart, 2013). In another study series using a bone marrow stem cell assay selected anion exchange fractions of sheep serum produced a significant increase in apoptotic cells, as judged by Annexin V and TUNEL assessments, without an increase in necrotic cells, suggesting a physiological effect rather than toxicity. The anti-proliferation and pro-apoptosis effects reported here involved rat bone marrow cells, a mixed population of cells chosen to model the mixed population of cells in whole organs and tissues. Is this in vitro assay valid as a proxy for the in vivo rat organometric assay? Using the latter an anti-organotrophic effect was tracked down to a sub-sub-fraction, the final stage of fractionation being anion exchange chromatography (Hart, 2003). For the adrenal PC12 cell exudate an anti-proliferative/pro-apoptotic influence was likewise evident in HPLC anion exchange material. This in vitro effect was neutralised by an anti-EPL001 antibody, as was an inhibitory effect in the in vivo kidney study (Haylor et al., 2009). These reflections indicate that the in vitro and in vivo changes are likely to be due to the same agent.

Conclusions depend on an antibody assessment. In IP an anti-EPL001 antibody generated a plethora of hits in a technique known for having this outcome. In IHC specificity ruled, with preabsorption effective. Preabsorption was also effective in preventing prior immunodepletion in vitro, supporting the interpretation that what was being neutralised was a single antigen. So the Ockhamist view adopted here is that the antigens in the IP, IHC and immunodepletion are one and the same or members of a single antigen family.

The IHC images speak of antibody specificity yet in a bead IP protocol the antibody was apparently anything but specific. Overwhelming amounts of data are however a feature of MS proteomics campaigns, with typically hundreds of proteins identified (Murphy & Gainer, 2016, pp. 155–169). An excursion into the fruit fly proved instructive, to gain insight into a simpler system, producing further images of seeming neuroendocrine relevance while indicating that the use of frozen unfixed tissue as starting material was to be eschewed in favour of formalin-fixed tissue, with antigen retrieval. In other words, cross-linking with formalin arrested degradation and proteolysis better than did freezing. There was no commonality between the rat ‘*unfixed* test overlappers’ (i.e. test-specific proteins in frozen PC12 and hypothalamic material considered together) and the rat ‘*fixed* test overlappers’ (i.e. test-specific proteins in formalin-fixed hypothalamic material subjected to antigen retrieval by either citrate or RIPA). These were different populations of proteins. Note in the context of the later discussion that granins do not figure in lists of proteins that bind nonspecifically in bead purifications (Trinkle-Mulcahy et al., 2008). A factor in MS analysis of precipitated analyte is occlusion, i.e. molecules being trapped nonspecifically by a macromolecular bolus. MS proteome analysis is usually less complex when an immunoaffinity column is used and this was found to be the case in a forerunner exercise. The approach was not especially favoured, though, as it had been previously demonstrated that anti-proliferative activity in vitro spreads itself across too many column fractions (Hart, 2013, Fig. 4 therein). Nonetheless a candidate did emerge from rat hypothalamic extract, a protein related to neurexophilin, NXPE family member 4 (Q5XI89, formerly FAM55D). Weakly correlating to the Edman data to which it was a ‘hit’ and weakly aligning with EPL001, this item could hardly be an entity purified by anion exchange chromatograpy, as required here, having a predicted pI of 9.2 (CBS, http://www.cbs.dtu.dk/services/SignalP/, http://www.cbs.dtu.dk/services/TMHMM/). In the IP campaign over-stringent washing was eschewed to avoid losing specific interaction partners (Trinkle-Mulcahy et al., 2008), although superfluity made analysis challenging. The MS analysis was likewise forgiving, accepting single peptide IDs, 95% confidence and slender epitopes, on the basis that it is more perilous to shun evidence than embrace it in an all-or-nothing hunt for an elusive factor. While high heterogeneity was evident in the hypothalamus formalin-fixed samples there was a dearth of secreted entities in the form of neuropeptides, hormones, growth factors and cytokines (Murphy & Gainer, 2016, pp. 25–56), including an absence of ligands associated with the canonical transcription factor families—WNT, TGF-β, GPCR, Notch, EGF, MAPK and Hedgehog—and proteins associated in any way with the anti-proliferative Hippo pathway. Myriad MS hits there may have been but one stands out: a proteoform of SgII, the acidic neuroendocrine secretory vesicle helper protein and prohormone. Since the SgII proteoform was the only secreted fixed test-specific item seen in rat hypothalamus using both antigen retrieval systems it is to that extent the only candidate. A granin identity is strongly supported by the IHC, more specifically as relating to SgII, as will be described. A granin identity is also strongly supported by the ultra-acidity of the form of the factor dislodged by a high salt concentration in anion exchange chromatography. But there is an insubstantial apparent homology between the synthetic antigen EPL001 and the proposed SgII-related endogenous antigen (the same point applies as between EPL001 and the *Drosophila* candidate antigen Q9W2X8). A route into this issue is offered by a molecular weight analysis.

Master rSgII is P10362. This protein has the SgII-related MS ID peptide so in principle could have been the SgII proteoform detected via IP. It also boasts the partial epitope triplet NNI, a conserved SgII motif that does not appear in CgA or any other granin. Yet SgII is found in the anterior pituitary (Kirchmair et al., 1993) and the mammalian antigen is not. Among other differences, SgII has not been visualised in the pancreas (Karlsson, 2001) whereas the endogenous antigen has. So the antibody is not seeing master SgII as such. We would therefore not expect to see a band of relevant size, ∼68 kDa (without signal peptide), in western blotting and we do not. Albeit a negative, this is the first of a multi-way MW concordance. Three bands were seen in fact in the western campaign on rat hypothalamic material, at 60–65, 35–45 and 7–8 kDa (preabsorption of the anti-EPL001 antibody was demonstrated for the mid and lower MW forms, using the epitope-containing *C*-terminus of EPL001; preabsorption was not attempted for the high MW form). The high MW correlates with the mass of the rSgII splice variant G3V7X2, which is ∼63 kDa (signal peptide deleted). The medium MW is a match for that of rSgIIvar, Q8CGL8, at ∼37 kDa. The lowest molecule weight item of 7–8 kDa has been described here as Circa-70. This can now be proposed as a SgII proteoform, SgII-70. This low MW band, besides appearing in an aqueous extract of rat hypothalamus, was the only one seen in western blotting of PC12 conditioned media (i.e. in the PC12 secretome) and sheep plasma. Why might there be three MWs at issue in the rat and only one in the sheep? The rat *Scg2* gene on chromosome 9 has three exons, giving rise to splice variants (RGD: Rat Genome Database, http://rgd.mcw.edu/; UCSC: UCSC Genome Browser, https://genome.ucsc.edu/index.html). In contrast the sheep *Scg2* gene on chromosome 2 comprises a single exon, which mono-exonic status is also true for the cow (Helle, 2004); hence no splice variants. The MW fit thus supports SgII relatedness, but there is more. P10362 comprises 619 aa. The rat splice variant G3V7X2 has 579 aa. It lacks a 40 aa mid-section after P10362’s arginine-317, *C*-terminal of NNI. Also lacking the mid-section 40 is the shorter splice variant rSgIIvar, Q8CGL8, at 322 aa. It can be suggested that this excision brings together the elements of a discontinuous epitope. This in turn invites the prediction that SgII-70 has the same discontinuous epitope. It is being proposed here, then, that the anti-EPL001 antibody sees three SgII-related 40-minus antigens in the rat and one in the sheep. What was bound by the antibody in rat hypothalamus IP was one of the splice variants, G3V7X2 or Q8CGL8. But the IHC staining seen in the sheep median eminence cannot be accounted for by these entities because they do not exist in the sheep, with its mono-exonic gene. The staining in the sheep hypothalamus must therefore be due to the postulated sSgII-70, which presumably arises as a result of proteolytic processing.

The epitope of the anti-EPL001 antibody within EPL001 was determined to be within the *C*-terminal KVKEFNNI. Preincubation of the antibody with EPL001 and separately KEFNNI eliminates IHC staining of neurons in rat and mouse brain (personal communication, David Howlett, King’s College London). This indicates that _9_KEFNNI_14_ is probably the (single) epitope in EPL001—although it could be shorter—and presumptively identifies the endogenous epitope. According to a BLAST search there is only one ovine protein with the motif KEFNNI: APOBEC1 complementation factor (XP_004020057.1), which is part of an mRNA editing enzyme complex, shuttling between nucleus and cytoplasm without being secreted. This did not appear in the IP/LC–MS and cannot be what the antibody is seeing (EFNNI is seen in sundry uninteresting ovine proteins, including dynein heavy chain and MHC proteins). On this analysis the anti-EPL001 antibody is probably seeing a discontinuous epitope, if it is seeing anything specifically in the sheep proteome. Rat SgII and fruit fly Q9W2X8 possess NNI as part of the minimal partial epitopes **V**xxx**NNI** and **K**xxxx**NNI**, respectively, both 4/14 (<30%). Sheep SgII provides only a bare NNI, however, indicating that the V in the rat SgII partial epitope is probably irrelevant. There is an antibody to CgA which is claimed to recognize just the three aa residues GAK (Corti et al., 1996). It is not suggested here, though, that NNI is the epitope in its entirety, as antibody binding sites are regarded as involving 5–10 amino acids (Lloyd, 2004, p. 1). In the TrEMBL database, accessed via UniProt (http://www.uniprot.org) using Perl scripts, about 4% of ovine proteins have NNI. The corresponding figure for the fruit fly is 7%. The possession of NNI is therefore not definitive and does not distinguish either protein on sequence grounds alone from the other test specifics bearing this motif in Tables 1 and 2 or from test-specific proteins with motifs such as EFN, KVK and so on. In the MW analysis above a discontinuous epitope was suggested. Given that the missing KEF motif does not appear in rSgII or sSgII (or in the fly protein) the endogenous epitope can be represented as a hypothetical 3D mixture of contiguous and non-contiguous aa thus: K·E·F·NNI. This has heuristic value as will now be described.

**Figure 12:**
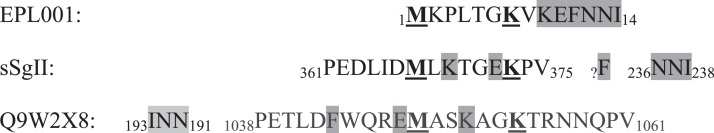
Sequence alignment of MxxxxxK doubletons. Doubleton in synthetic antigen EPL001, with matches in endogenous candidate antigens—SgII-related in mammals, Q9W2X8 in the fruit fly—with residues shaded having proposed relevance to the epitope of the anti-EPL001 antibody.

There is a feature of the SgII primary sequence that differentiates ‘SgII relatedness’ from the candidacy claims of all other mammalian proteins analysed: a shuffled version elsewhere in the protein of most of the absent EPL001 residues. The relevant motif in sSgII is this: _367_MLKTGEKPV_375_. This string is 9 of the 11 residues from EPL001 that remain if NNI is subtracted from the total of 14, the unrepresented items being an F and a K. The corresponding 9mer motif in rSgII is represented by residues 369–378. These are situated just *C*-terminal of the 40 rSgII residues (317–358) absent from the rat SgII splice variants, with _239_**NNI**_241_ *N*-terminal of it. The 9mer motifs in sheep and rat SgII are part of the second sorting domain (Courel et al., 2008). There is an alignment of **M**xxxxx**K** between EPL001, the sSgII 9mer and the homologue of SgII’s second sorting domain within fly Q9W2X8 (Fig. 12). A three-way match between a synthetic antigen (EPL001) and a pair of proposed endogenous antigens from different phyla is unlikely to be a coincidence. Q9W2X8 and sSgII also share **PV**. These are two particularly important residues as they were detectable by Edman in sheep plasma even when most of the ovine factor’s other residues were invisible (see ‘Bioinformatics’). The sSgII 9mer, _367_MLKTGEKPV_375_, proves worthy of further analysis. The shaded residues match those from the front half of EPL001, in the form of three doublets, separated by singleton matches to residues in the second half of EPL001. The first doublet has the initial M of EPL001; the second and third are exact matches for doublets within EPL001, albeit in different positions. That the pattern seems meaningful further argues against occurrence by chance, as does a probability estimate.

The number of different 9mer permutations that can be provided by the EPL001 11mer is 3,507,840 or ∼3.5 × 10^6^. (This number includes the 1 × 9! cases where all the residues are different, i.e. where there is 1K, as well as the 8 × 9!/2! cases where there are 2Ks and the 28 × 9!/3! cases where there are 3Ks. Totalling, this equals 9.67 × 9!.) There are some 27,000 entries in the TrEMBL database of proteins for *Ovis aries*, with an average length of 481 aa. To simplify the calculation consider a hypothetical ovine protein of 500 aa, comprising 25 each of the 20 aa in random order. The number of 9mers that can be provided by the hypothetical protein is 5.12 × 10^11^. The 3.5 × 10^6^ permutations can now be divided by 5.12 × 10^11^ to yield the probability of there being in the hypothetical protein a 9mer from the EPL001 11mer as 6.85 × 10^−6^ or approximately 1 in 146,000.

Assuming that the shuffle-sequence 9mer is relevant, a discontinuous epitope within sSgII can be deduced from the second sorting domain and the NNI landmark as _369_K·_372_E·_?_F·_236_N_237_N_238_I (Fig. 12). A speculative epitope by analogy within fly Q9W2X8 would be _1051_K·_1047_E·_1043_F·_191_N_192_N_193_I. The NNI motif in SgII can be suspected as itself having sorting domain relevance (see ‘Bioinformatics’), while the 9mer certainly has, being part of the SgII second sorting domain. So ‘sorting domain chemistry’ might be a relevant concept here, as might ‘extensive proteolysis and potentially other post-translational modifications’ and perhaps ‘Edman misread,’ to explain the provenance of the EPL001 sequence. Further research is required.

What of the SgII-derived peptides SN, EM66 and manserin? Lacking the MS ID peptide, none of these could have been what was seen in the rat hypothalamus IP. SN and manserin are too small, at 33 and 40 residues apiece, to account for SgII-70 as seen in western blotting. At 66 aa EM66 is slightly below the predicted length but it alone of the three has the NNI motif—though not the 9mer shuffled sequence. EM66 is found in the chromaffin cells of human adrenals and in pheochromocytomas (Yon et al., 2003) and is a diagnostic marker for the latter in plasma (Guillemot et al., 2006). EM66 immunostaining is seen in discrete parts of the hypothalamus, with the median eminence displaying a dense plexus of nerve fibres (Boutahricht et al., 2007) in a manner highly reminiscent of the present study. But EM66 is found in the pituitary (Yon et al., 2003), unlike the antigen in the current work. It is involved in the feeding behaviour in rodents (Boutahricht et al., 2007), another discordancy. The conclusion is that EM66 and SgII-70 are not the same entity.

For the mammalian antigen to be a granin, localisation to intracellular secretory granules would be expected. Intracellular speckling is visible in focal neuroendocrine cells staining in the human colon (Fig. 3H), which cells matched in position those staining in a serial section for CgA as a neuroendocrine marker (I). The generality of tumour staining seen with granins (Taupenot, Harper & O’Connor, 2003) is highly reminiscent of the tumour staining reported here (and exhibited in Supplementary IHC Images). There were examples where negative staining in the normal tissue contrasted with positive staining in tumours at the same location: skin melanoma, breast carcinoma, oesophageal carcinoma. It appears paradoxical that a hypothesized inhibitor be up-regulated in tumours, but not if that factor were a granin, as loss of tumour suppressor or other negative intracellular regulators can potentiate a granin secretory phenotype (Expasy ProtParam, http://web.expasy.org/protparam/).

Further supporting the SgII relatedness of the present work, heat only partially reduced activity in an in vitro assay of bone marrow stem cell proliferation. Granins display as a feature thermostability (Taupenot, Harper & O’Connor, 2003). The low salt/high salt 7–8 kDa forms determined in anionic exchange chromatography fractions by western blotting could be accounted for by differential charge distribution of the kind seen in SgII and particularly associated with amphipathic helices and sorting domains (Courel et al., 2008). A receptor identification study using EPL001 involved mouse brain (Davies et al., 2015). No conventional receptors were identified. Instead EPL001 was found to bind to neuronal growth-associated protein 43, GAP-43. This would represent a non-traditional receptor, such as have been hypothesized for granins (Expasy ProtParam, http://web.expasy.org/protparam/).

That there was only one band on a gel in the fly purification and that it was credibly at the limit of resolution was encouraging, as was its rough concordance with the MW of Q9W2X8. Perplexity supervened however until the mammalian antigen was appreciated as SgII related. This prompted the realisation that Q9W2X8 has granin-like characteristics of its own, in spite of a general lack of sequence homology and an attribution in the databases to the spliceosome. BLAST searches reveal that homologues of mammalian Cgs and Sgs are absent from *D. melanogaster*. IHC staining is seen in fruit flies however using anti-CgA antibodies (Montero-Hadjadje et al., 2008), but the antigen has not been defined. The case for Q9W2X8 being itself a granin is as follows: IHC visualisation to glial and neurendocrine cells, with punctate cytoplasmic localisation, possibly in the ER; coiled coil structure and absence of disulphides, both granin features, along with a disproportionate intensity of clustered negatively charged residues (D and E), yielding an acidic pI; cross-phylum landmark sequence correspondencies, including the presence of homologues of SgII’s two main granule sorting domains (with the putative fly domains both having AxKAGKxxxxQ, the KAG of which is a match for the second sorting domain of rSgII); and 21 dibasic (R and K) proteolytic cleavage sites, a granin-like intensity, suggesting that Q9W2X8 might have peptide progeny. Expression of *CG43902*, the gene that encodes Q9W2X8, is in waves, with a peak in late embryological stages and another in pupae (FlyBase, http://www.flybase.org/). This implies a role for the protein in larvagenesis and metamorphosis, with inhibitory influence (see ‘Results’, ‘Bioinformatics’). ‘Embryo’ is, though, in last position in a list of the top 10 tissues by gene expression levels for Q9W2X8 in *Drosophila* (Genevisible, http://genevisible.com/search). Highest expression levels have been recorded in adult and larval brains. Below the generalised items of ‘adult head,’ ‘adult tagma’ and ‘first instar larva,’ there is ‘ovary.’ Of additional endocrinological significance is the likely staining reported here of the corpus cardiacum, a neurohaemal structure replete with hormones. That Q9W2X8 can potentially be understood by reference to mammalian SgII bolsters both fly and rat candidatures. It was particularly unexpected that homologues were noted in Q9W2X8 and rSgII on the basis of the MS identification peptide for each candidate; in other words, each had a version of the other’s identifier, in similar positions in the molecule. Q9W2X8 and SgII seem covertly related: the last common ancestor of insects and mammals >500 m years ago evidently sported a palaeogranin. A suggested name for the twice-SgII-size fly protein is macrogranin I (MgI).

## Conclusion

The hypothesis that the EPL001 amino acid sequence is meaningful and relevant in the context of an inhibitory hormonal influence is upheld. In the mammalian context what was sought was a cytoplasmic factor that is secreted to anti-organotrophic endocrinological effect. Providing this to a first approximation is ‘likely SgII relatedness,’ a conclusion directly supported by the results of the IP/LC–MS campaign and indirectly by IHC, acidic factor characteristics disclosed during purification, a multi-way MW concordance, a discontinuous epitope analysis and fruit fly homology, which last-mentioned deduction potentially discloses the fly protein Q9W2X8 as a granin. Particularly worthy of further investigation in connection with tissue-mass inhibition is a possible new 70-residue peptide product of SgII, SgII-70.

## Supplemental Information

10.7717/peerj.3833/supp-1 Supplemental Information 1 Supplementary IHC Images. Mammalian and *Drosophila* IHC images indicating the tissue distribution of the endogenous antigen or antigens to the anti-EPL001 antibodies.

10.7717/peerj.3833/supp-2 Supplemental Information 2 Mass spectrometry data sample key. Provided here is a key to eighteen Raw Data files, RD 1-18. The raw data depository also includes three files (RD 19-21) relating to the mammalian and fruit fly lead candidates.

10.7717/peerj.3833/supp-3 Supplemental Information 3 Drosophila unfixed control. Total hits 700.

10.7717/peerj.3833/supp-4 Supplemental Information 4 Drosophila test unfixed. Total hits 1129.

10.7717/peerj.3833/supp-5 Supplemental Information 5 Drosophila unfixed test and control samples compared. Test specifics 526.

10.7717/peerj.3833/supp-6 Supplemental Information 6 Drosophila fixed control. Total hit 634.

10.7717/peerj.3833/supp-7 Supplemental Information 7 Drosophila fixed test. Total hits 71.

10.7717/peerj.3833/supp-8 Supplemental Information 8 Drosophila fixed test versus controls. Fixed test specifics 30.

10.7717/peerj.3833/supp-9 Supplemental Information 9 Pheochromocytoma unfixed control. Total control hits 1095.

10.7717/peerj.3833/supp-10 Supplemental Information 10 Pheochromocytoma test unfixed. Total test hits 1516.

10.7717/peerj.3833/supp-11 Supplemental Information 11 Pheochromocytoma unfixed test versus control. Test specifics 603.

10.7717/peerj.3833/supp-12 Supplemental Information 12 Hypothalamus unfixed (no antigen retrieval) control. Total control hits 1634.

10.7717/peerj.3833/supp-13 Supplemental Information 13 Hypothalamus unfixed (no antigen retrieval) test. Test specifics 1681.

10.7717/peerj.3833/supp-14 Supplemental Information 14 Hypothalamus unfixed (no antigen retrieval) test versus control. Test specifics 424.

10.7717/peerj.3833/supp-15 Supplemental Information 15 Hypothalamus fixed (RIPA antigen retrieval) control. Control total 226.

10.7717/peerj.3833/supp-16 Supplemental Information 16 Hypothalamus fixed (RIPA antigen retrieval) test. Test total 406.

10.7717/peerj.3833/supp-17 Supplemental Information 17 Hypothalamus fixed (RIPA antigen retrieval) test versus control. Test specifics 213.

10.7717/peerj.3833/supp-18 Supplemental Information 18 Hypothalamus fixed (Citrate antigen retrieval) control. Control total hits 438.

10.7717/peerj.3833/supp-19 Supplemental Information 19 Hypothalamus fixed (Citrate antigen retrieved) test. Test total hits 454.

10.7717/peerj.3833/supp-20 Supplemental Information 20 Hypothalamus fixed (Citrate antigen retrieved) test versus control. Test specifics 114.

## Additional Information and Declarations

### Data Availability

The following information was supplied regarding data availability: The mass spectrometry data has been uploaded as Supplemental Dataset Files.
