# Supplementary material for: Mysterious inhibitory cell regulator investigated and found likely to be secretogranin II related
Source: PeerJ. 2017 Oct 13;5:e3833. doi: 10.7717/peerj.3833 (PMC5642266; doi:10.7717/peerj.3833)
Supplement: Supplemental Information 1 — Mammalian and Drosophila IHC images indicating the tissue distribution of the endogenous antigen or antigens to the anti-EPL001 antibodies. [file peerj-05-3833-s001.pdf]

Hart JE, Clarke, IJ, Risbridger GP, Ferneyhough, B,Vega-Hernández, M.
Mysterious inhibitory cell regulator investigated and found likely
to be secretogranin II related.

Supplementary IHC Images

Mammalian and *Drosophila* IHC images indicating the tissue distribution of the endogenous antigen or antigens of the anti-EPL001 antibodies.

See article text for methodology and interpretation.

Figs. 1-30 are based on the use of the rabbit polyclonal anti-EPL001 antiserum ER88.
Figs. 31-38 involved the use of the goat polyclonal anti-EPL001 antiserum G530.

******

Key

'micrin' = The unknown mammalian antigen to the anti-EPL001 antisera in IHC is herein denoted thus by way of shorthand, implying no preconceptions as to chemical identity. (Ref: Hart, JE. The body has a brake: micrin is a postulated new gonadal hormone curbing tissue overgrowth and restricting reproduction. Med Hypotheses. 2014; 83: 775-786.)

NE = Neuroendocrine

CgA = Chromagranin A

******

The thesis of the paper is that the observed anti-organotrophic hormonal activity is likely secretogranin II related. Fig. 1 is not evidential in this regard but is provided to apprise colleagues of the form in which the array tissues, human normal and tumour, were supplied. Other tissues were sourced non-commercially.

Figure 1: Slides of human tissue array

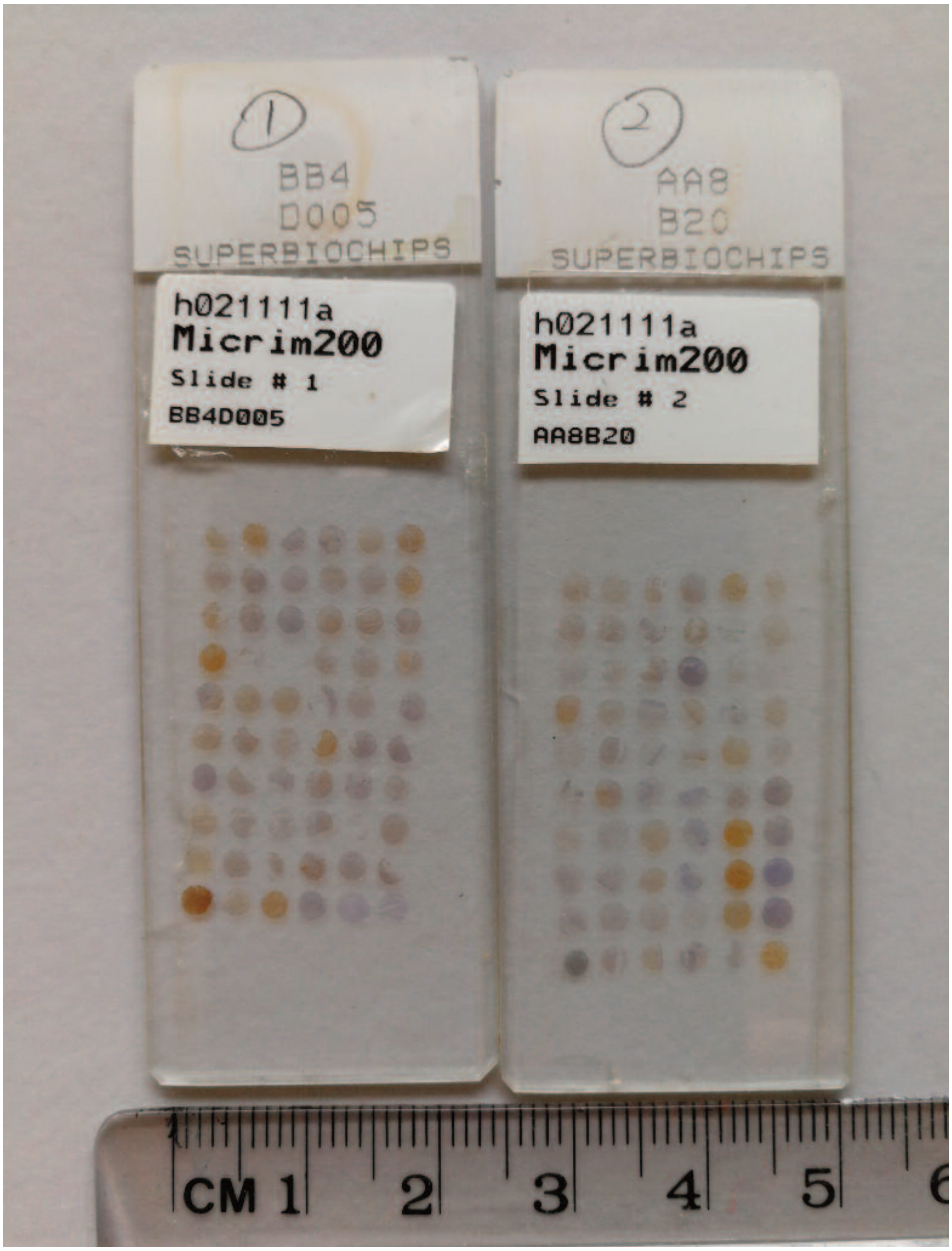

Figure 2: Tissues - ve stained with micrin on the normal tissue array

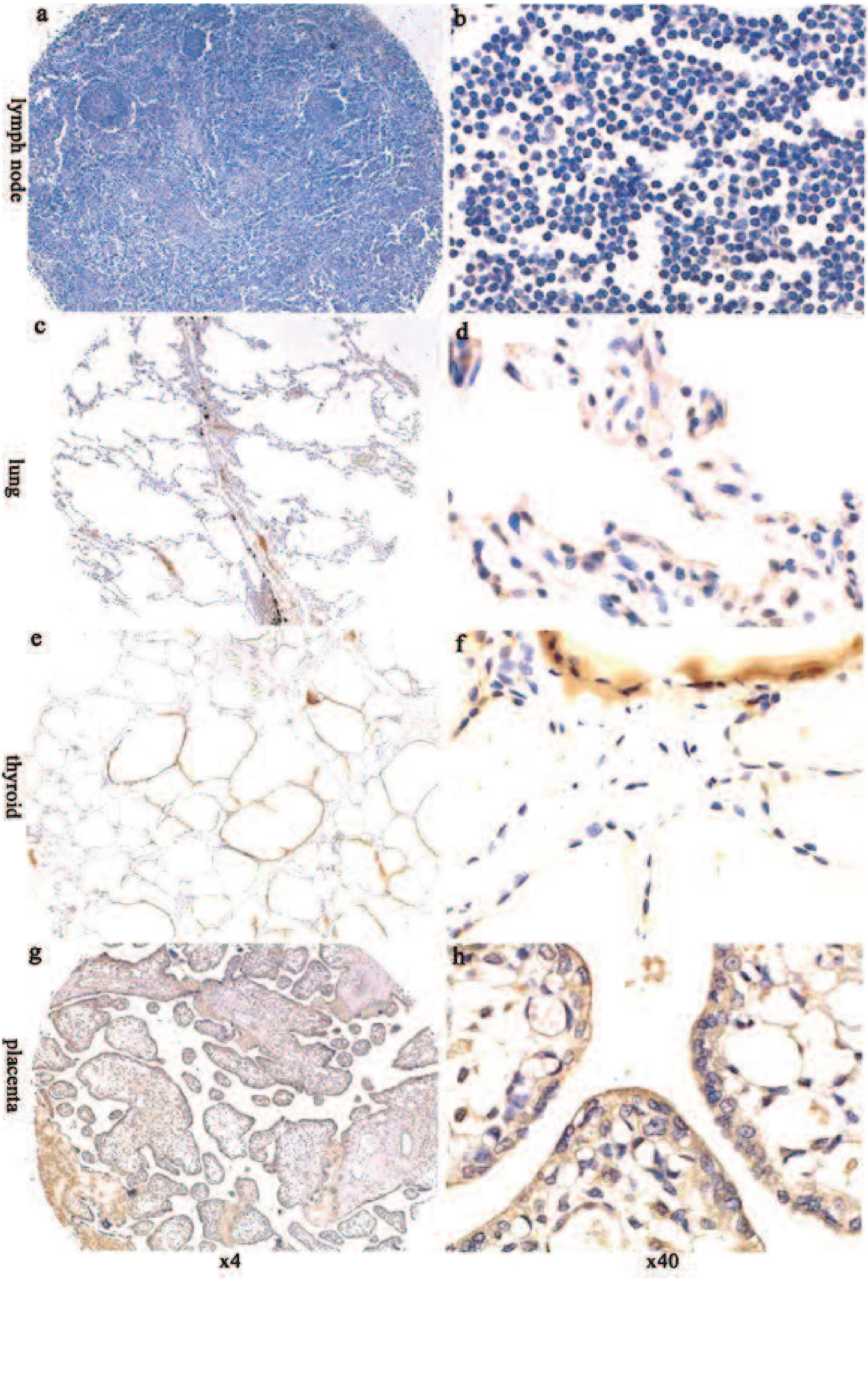

Figure 3: Weakly stained with micrin on the normal tissue array

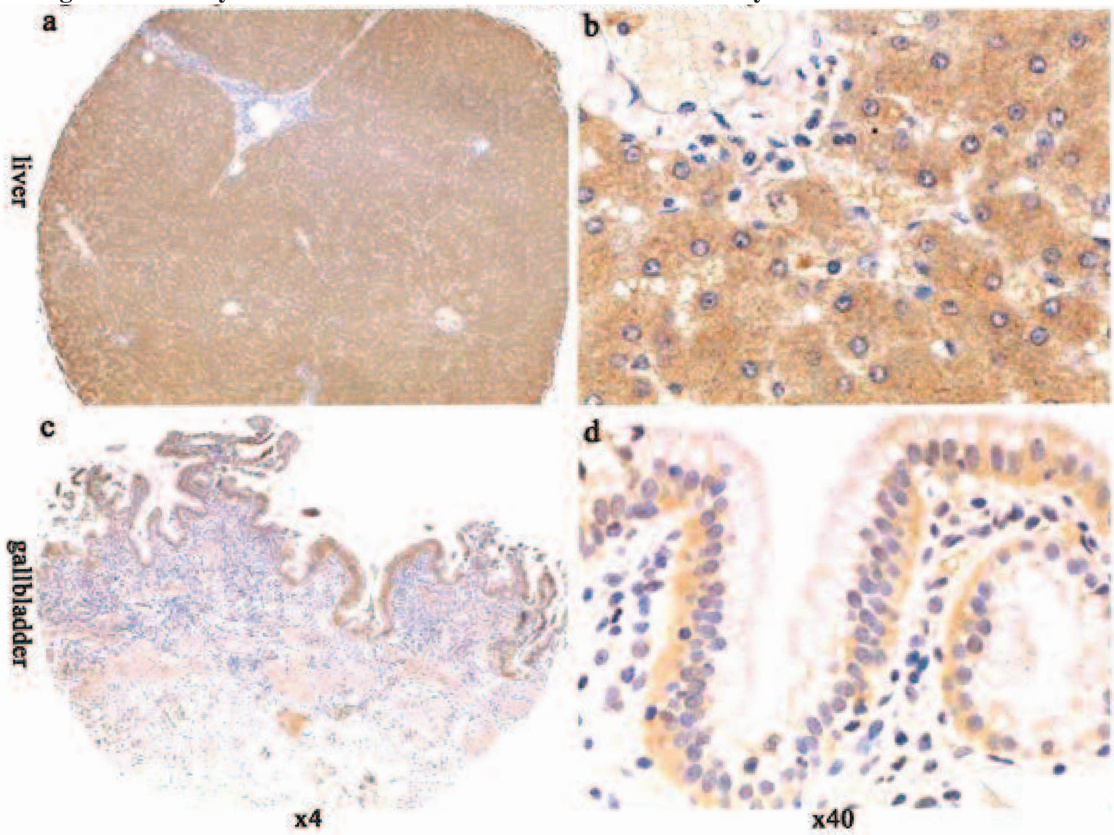

Figure 4: NE cells stained with micrin in prostate and gastrointestinal tract

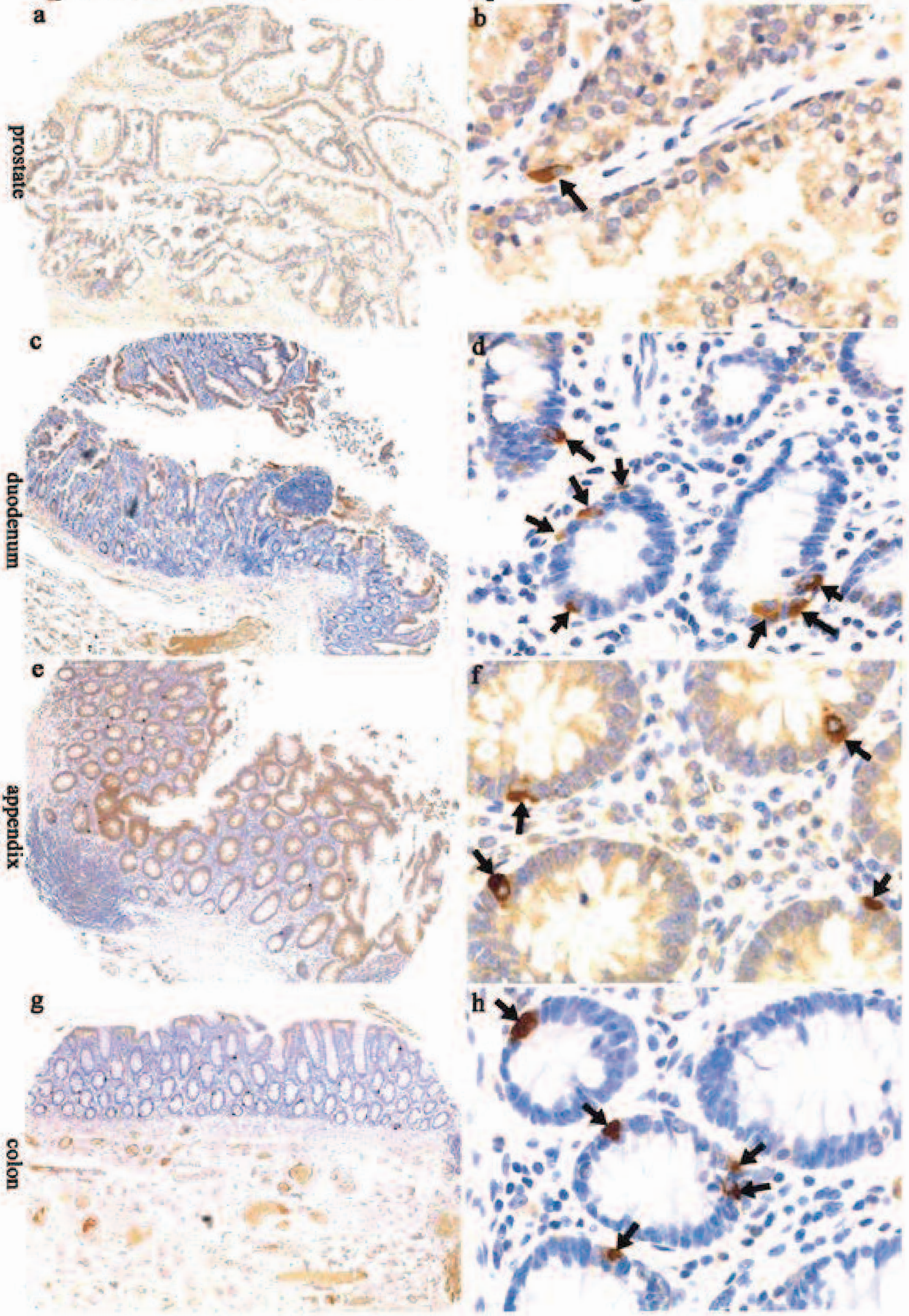

Figure 5: Serial sections stained with micrin and CgA

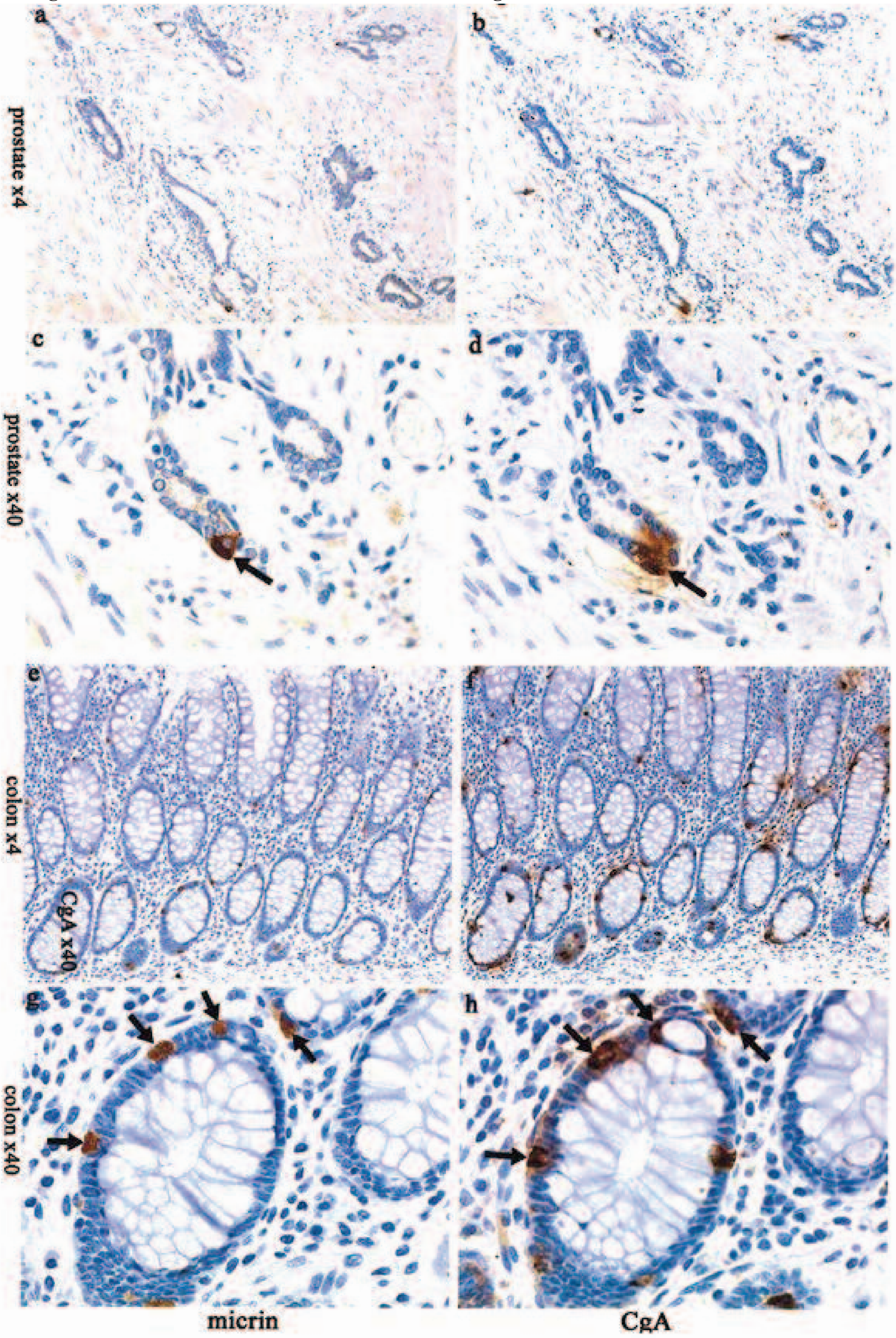

Figure 6: Tissues +ve stained with micrin on the normal tissue array

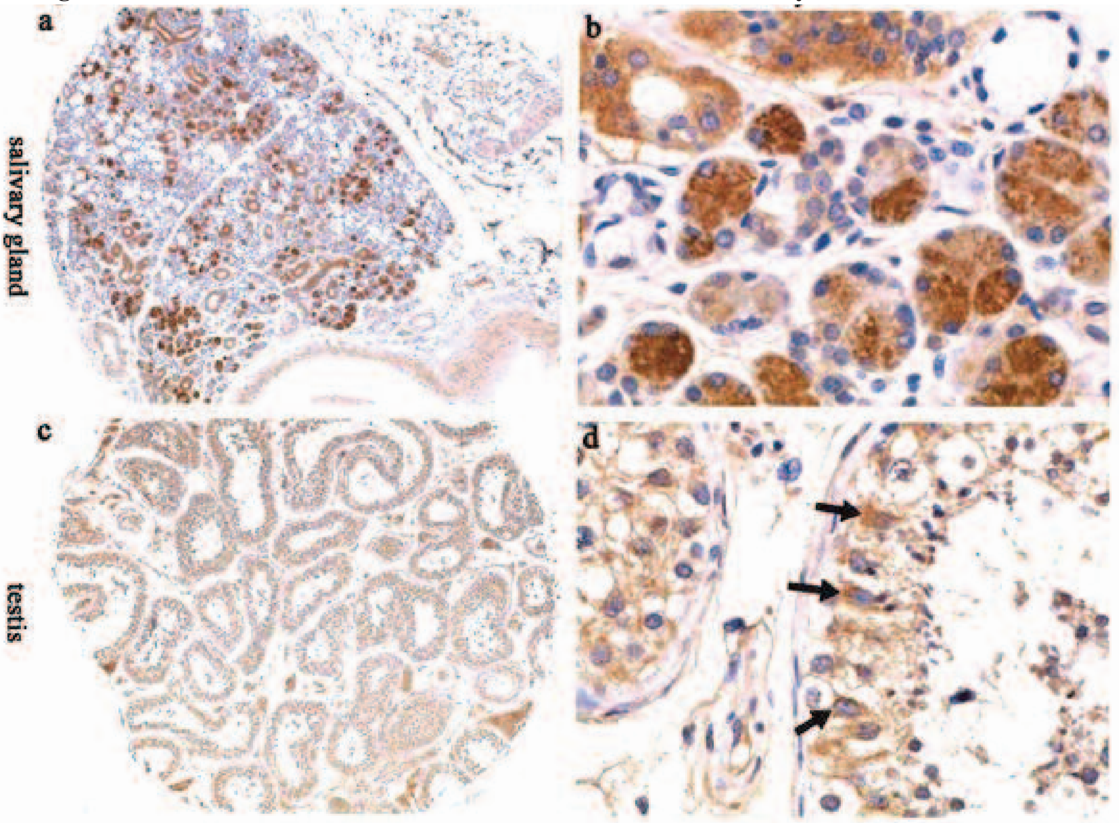

Figure 7: Micrin staining with preabsorption on the human prostate samples

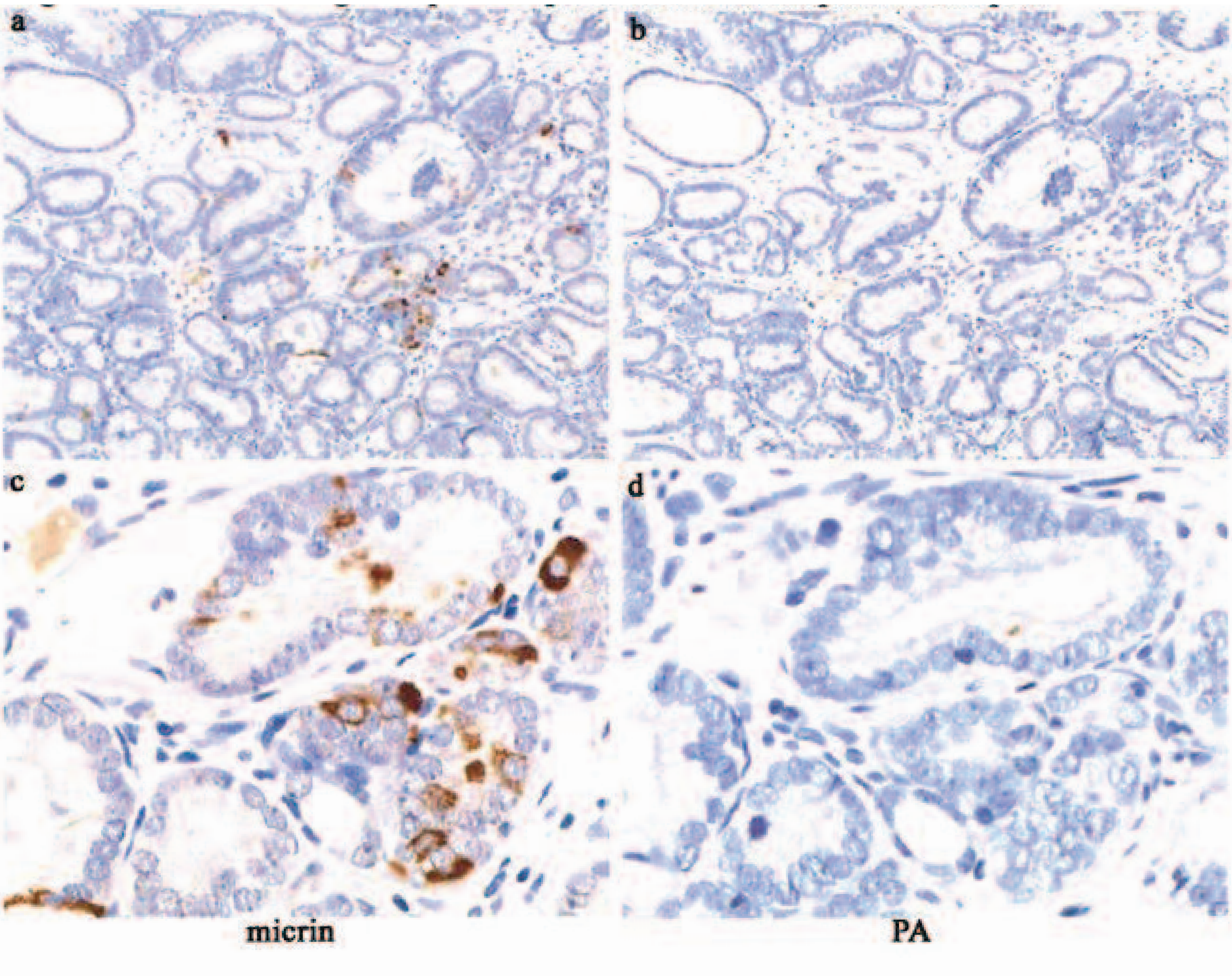

a & b x10

c x d x40

Antibody at 1/200

PA = preabsorption with EPL001 peptide at 0.5 mg/ml

Figure 8: Tumor tissues +ve stainied for micrin

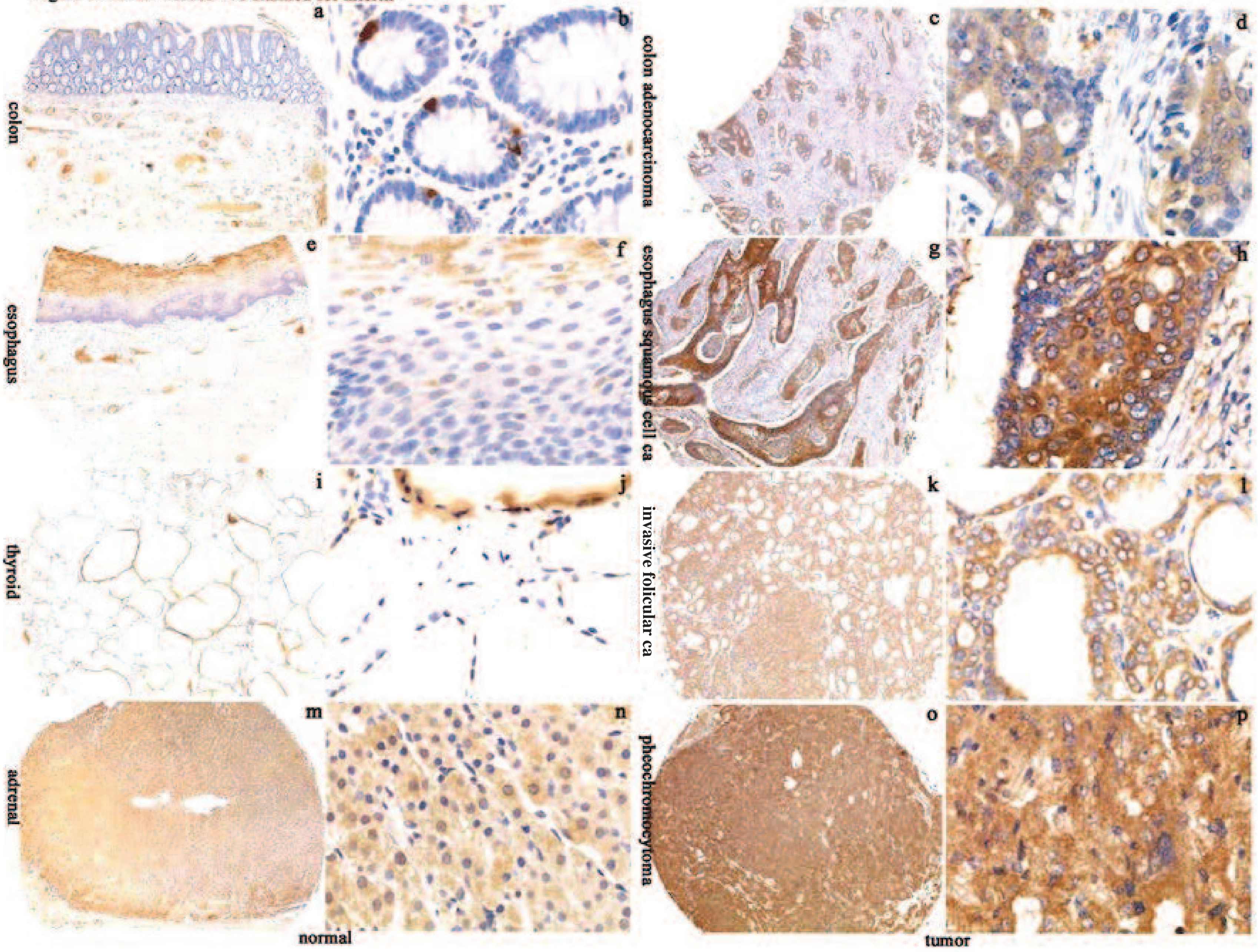

Figure 9: Immunostaining micrin and CgA on human radical serial sections prostate

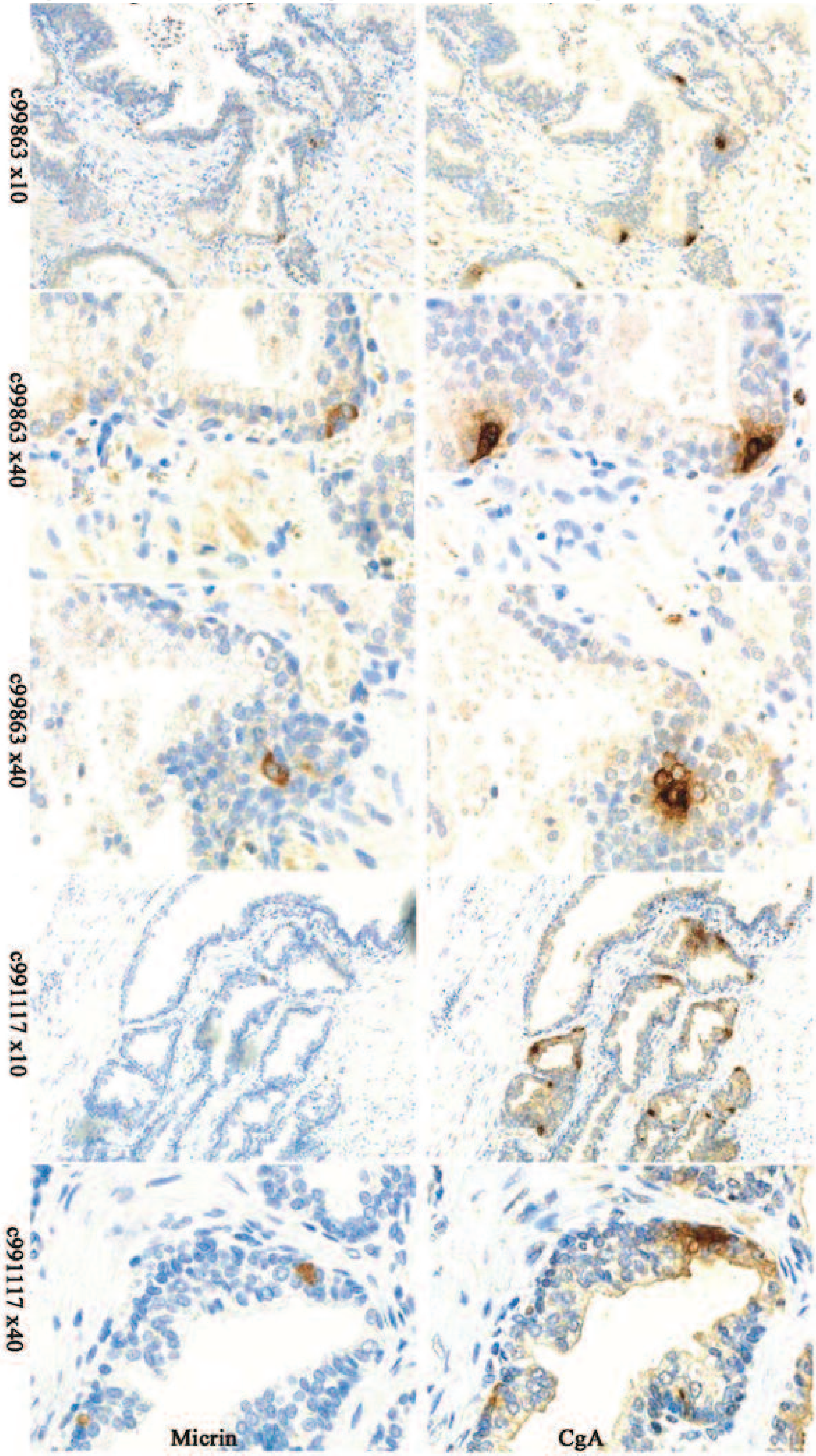

Figure 10

Normal | Tumor

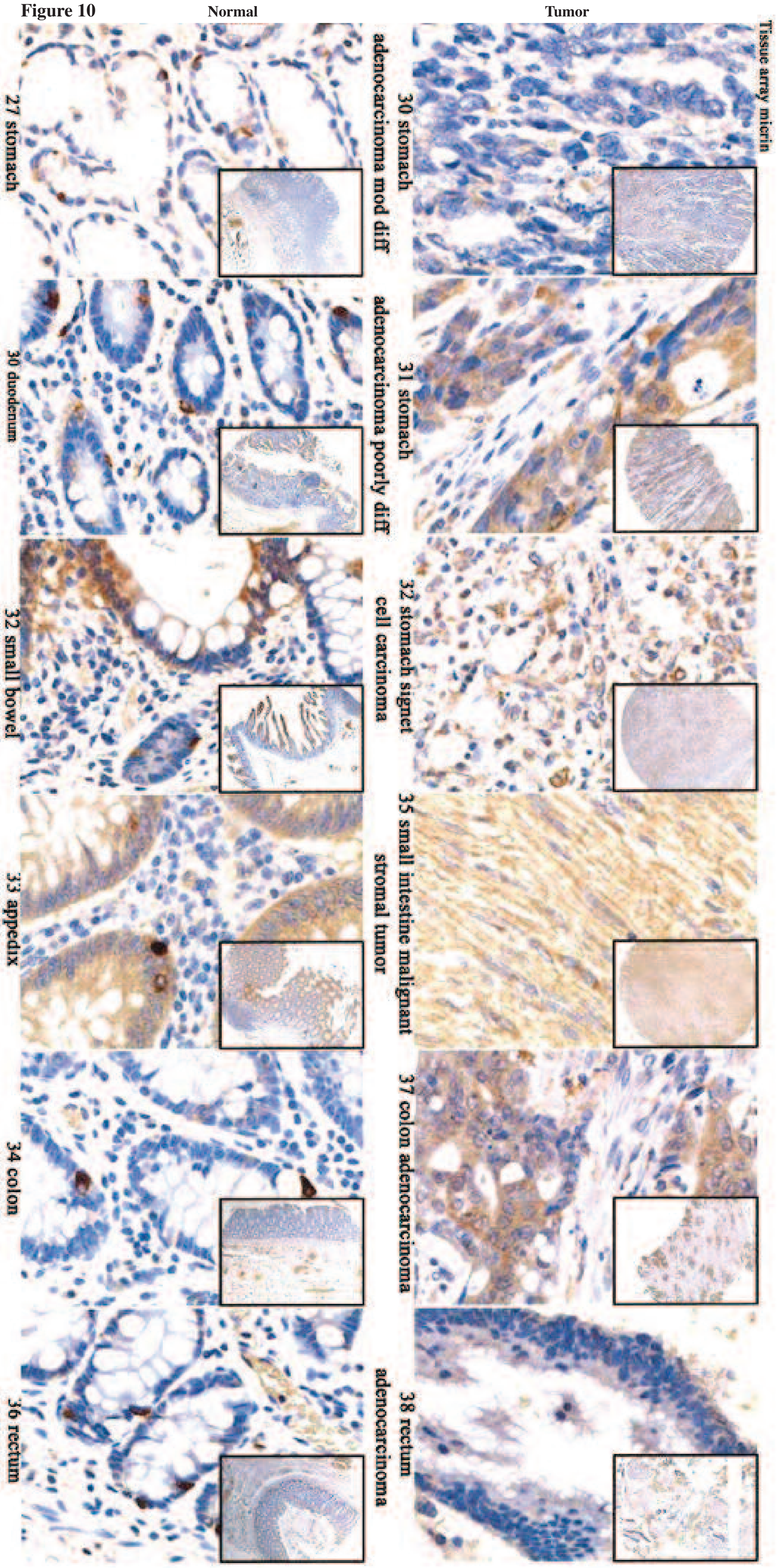

Figure 11 Normal Tumor

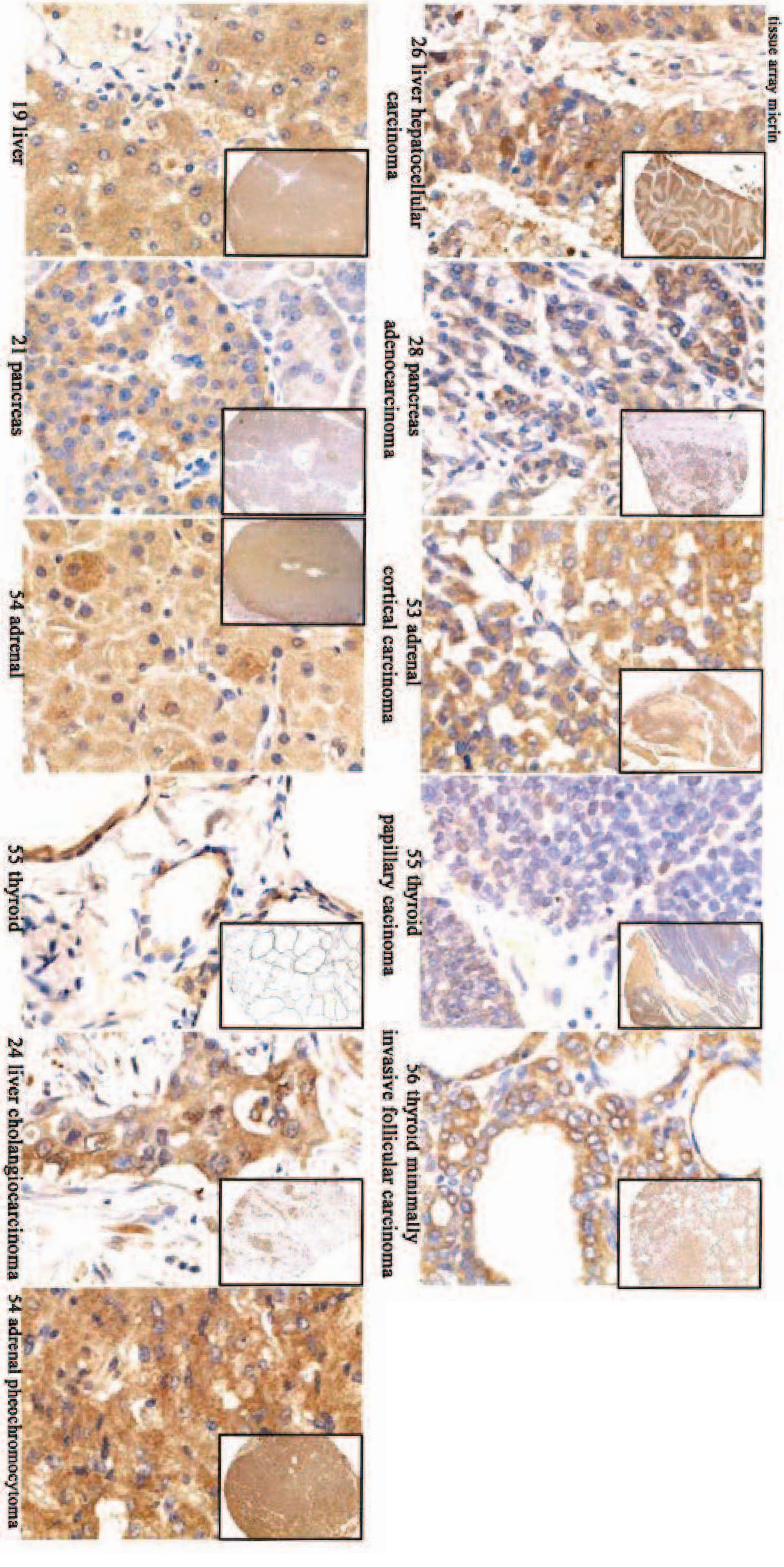

Figure 12

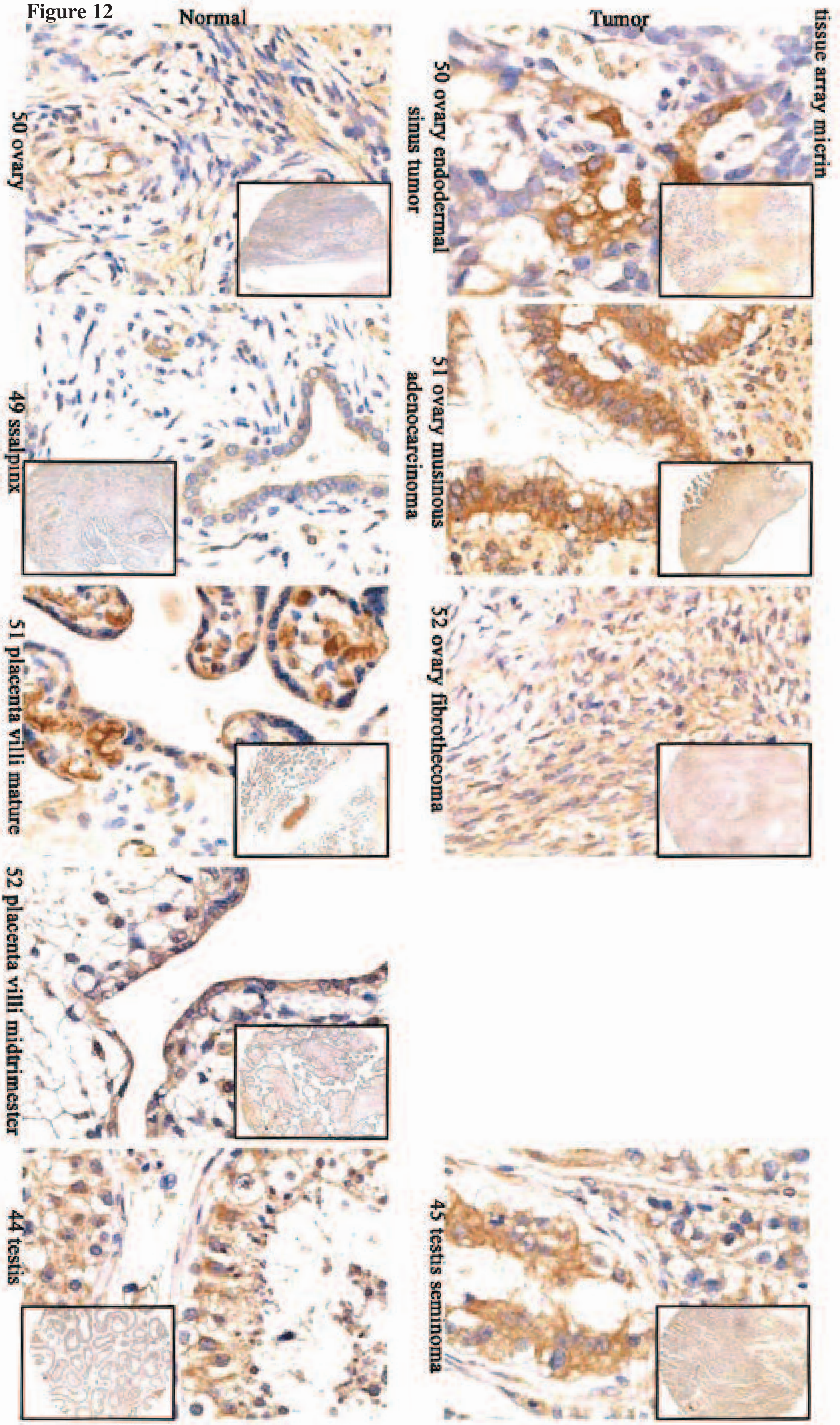

Normal
Tumor
tissue array micrin
50 ovary
50 ovary endodermal sinus tumor
49 ssalpinx
51 ovary musinous adenocarcinoma
51 placenta villi mature
52 ovary fibrothecoma
52 placenta villi midtrimester
44 testis
45 testis seminoma

Figure 13

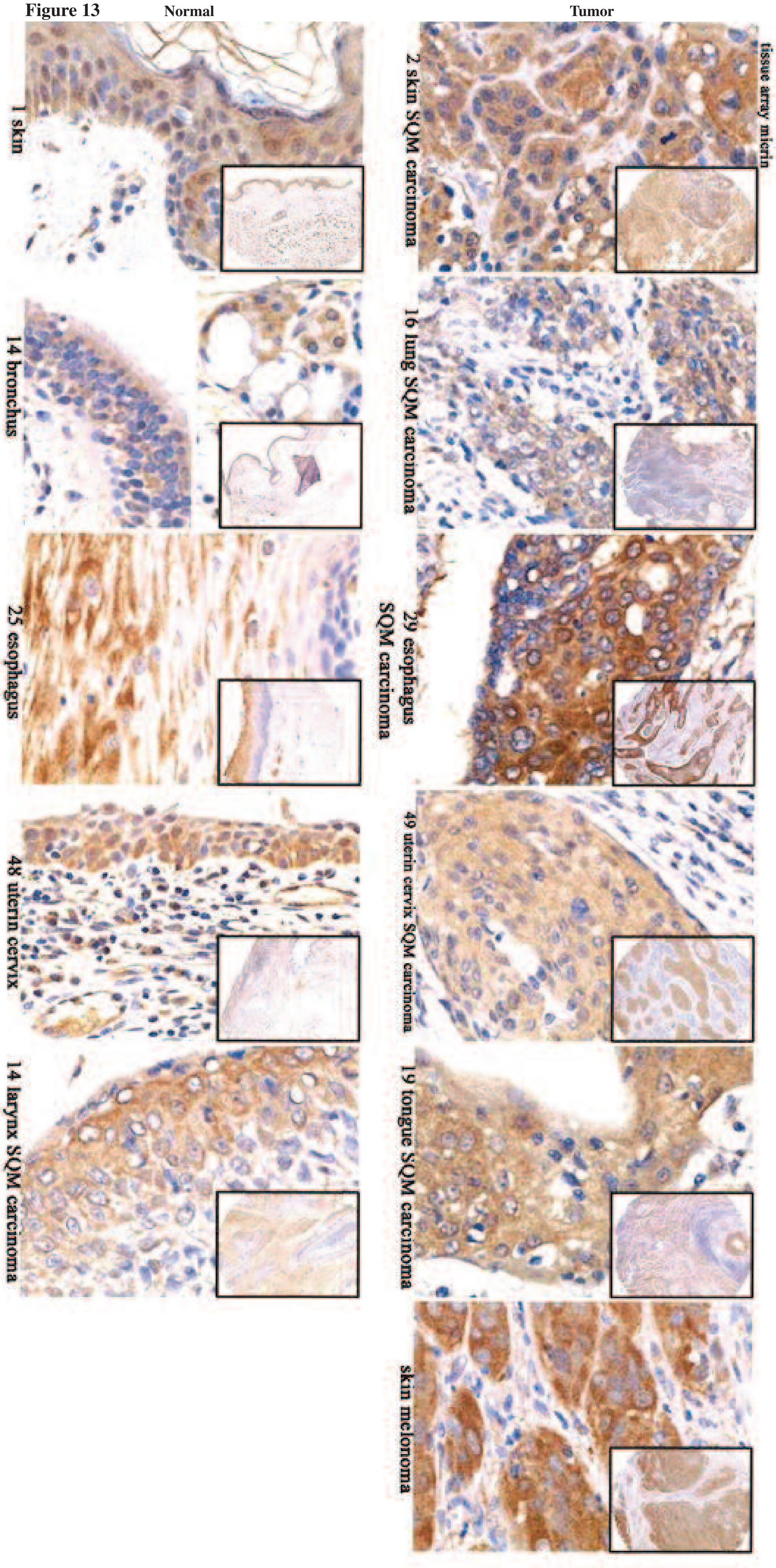

Normal
Tumor
tissue array micrin
1 skin
14 bronchus
25 esophagus
48 uterin cervix
14 larynx SQM carcinoma
2 skin SQM carcinoma
16 lung SQM carcinoma
29 esophagus SQM carcinoma
49 uterin cervix SQM carcinoma
19 tongue SQM carcinoma
skin melonoma

Figure 14

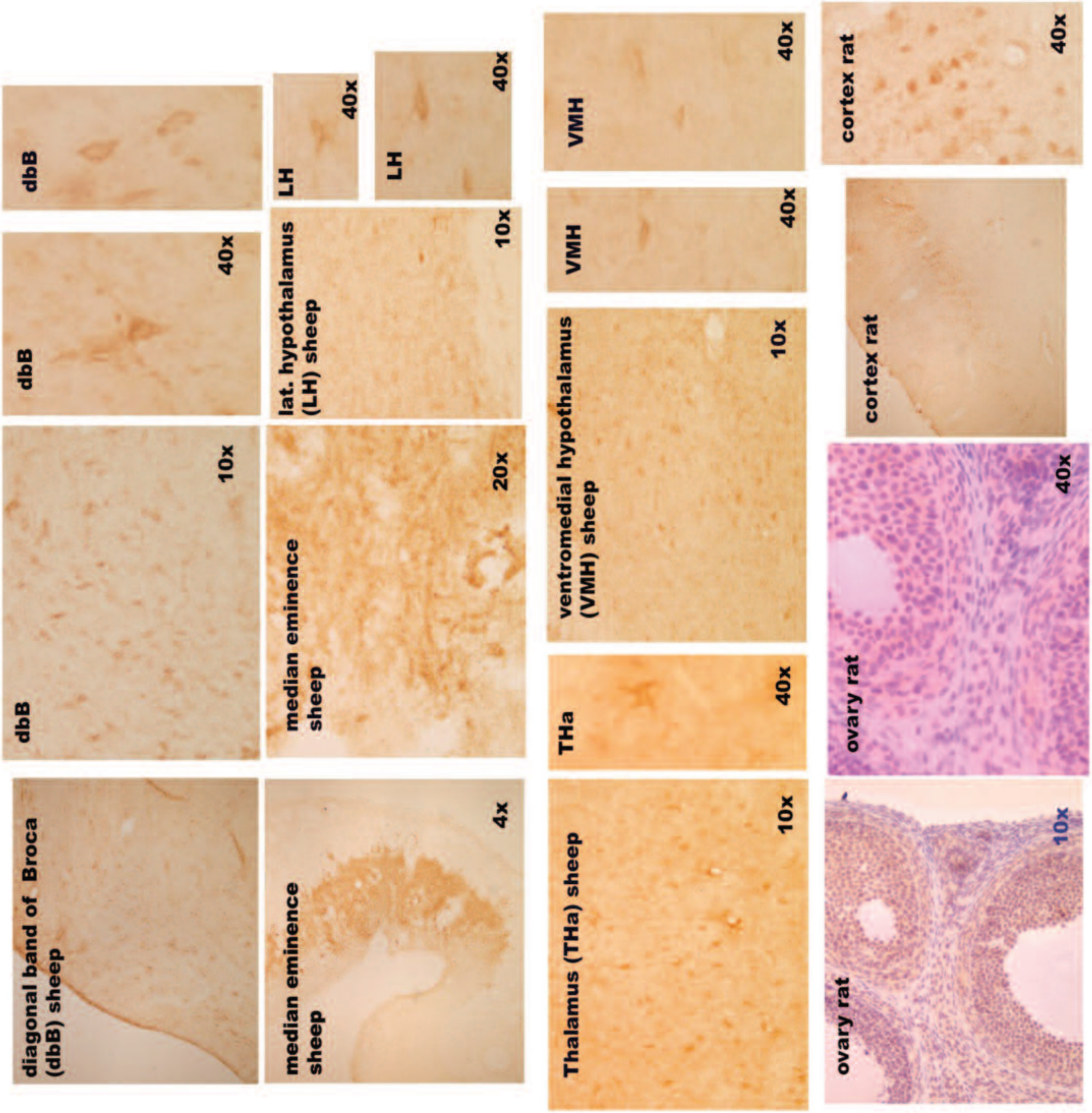
diagonal band of Broca (dbB) sheep
dbB
10x
dbB
40x
dbB
median eminence sheep
4x
median eminence sheep
20x
lat. hypothalamus (LH) sheep
10x
LH
40x
LH
40x
Thalamus (THa) sheep
10x
THa
40x
ventromedial hypothalamus (VMH) sheep
10x
VMH
40x
VMH
40x
ovary rat
10x
ovary rat
40x
cortex rat
cortex rat
40x

Figure 15

Normal Human Kidney: IHC

(ER88 rabbit polyclonal antibody)

. Haylor JL, Parker E, Risbridger GP, Beale D, Brown BL, Dobson PRM, Clarke IJ, Hart JE. Inhibition of compensatory renal growth by the N-terminus of a sheep-derived peptide. Regul Peptides. 2009; 152: 48-53.

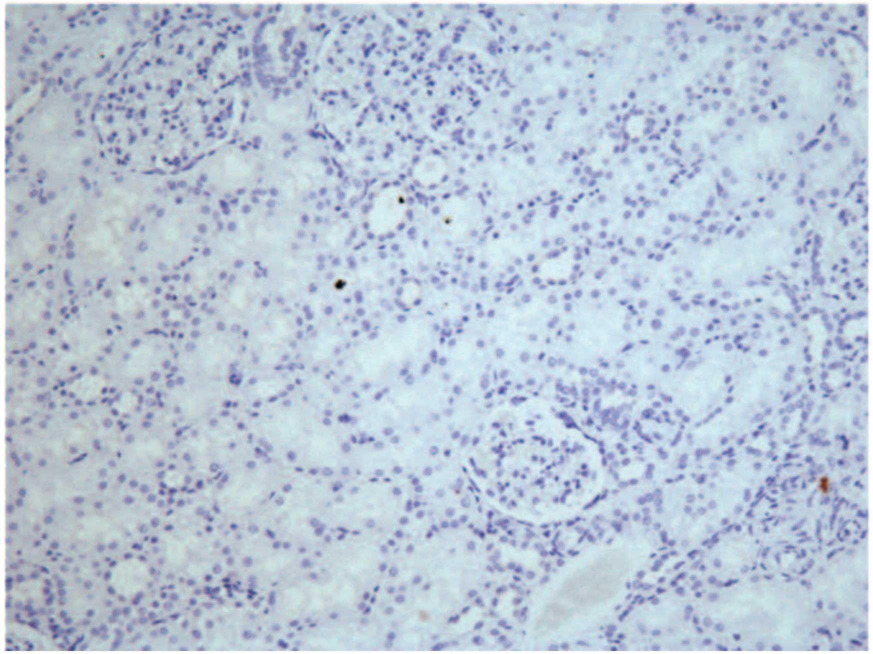

Preabsorbed Antibody

100μg/ml

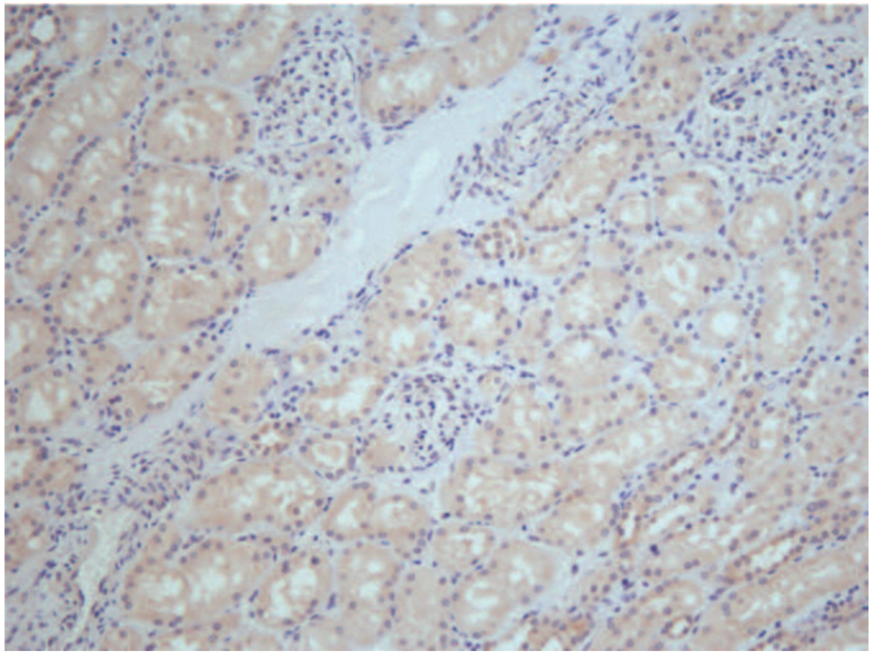

Magnification x100

Figure 16

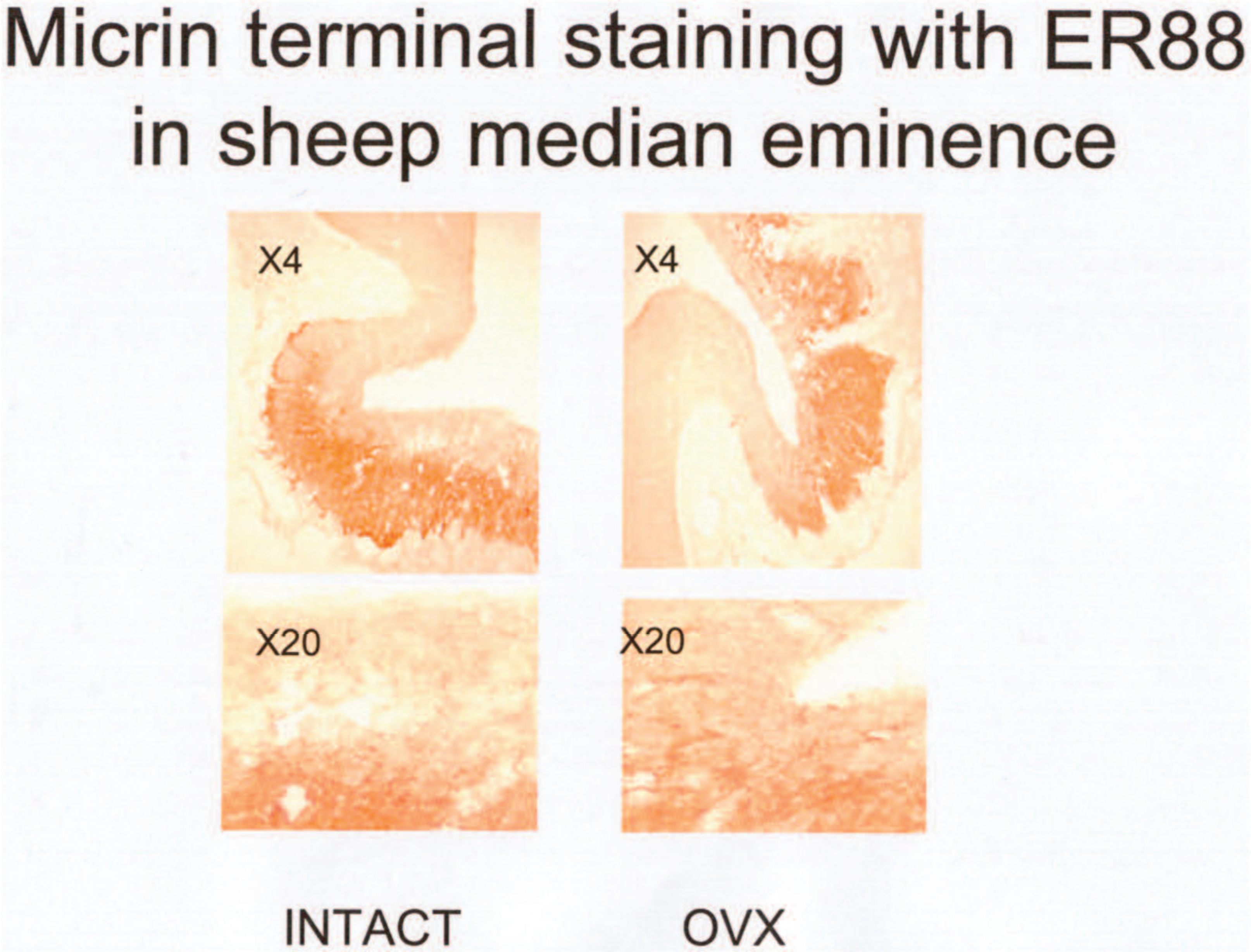

No difference seen between ovary intact and ovariectomised ewes in IHC staining of hypothalamic median eminence.
Note: ‘Terminal’ refers to the antibody being raised to the N-terminus of a purified ovine protein

Figure 17

Immunohistochemistry for Micrin

- Rabbit antibody used at 1/100 to 1/1000
- DAB visualisation
- No staining with pre-immune serum
- Immunochemically stained cells in sheep median eminence (ME), sheep ovary (corpus luteum), rat ovary (theca and granulosa) and human prostate (basal luminal cells)

Figure 18

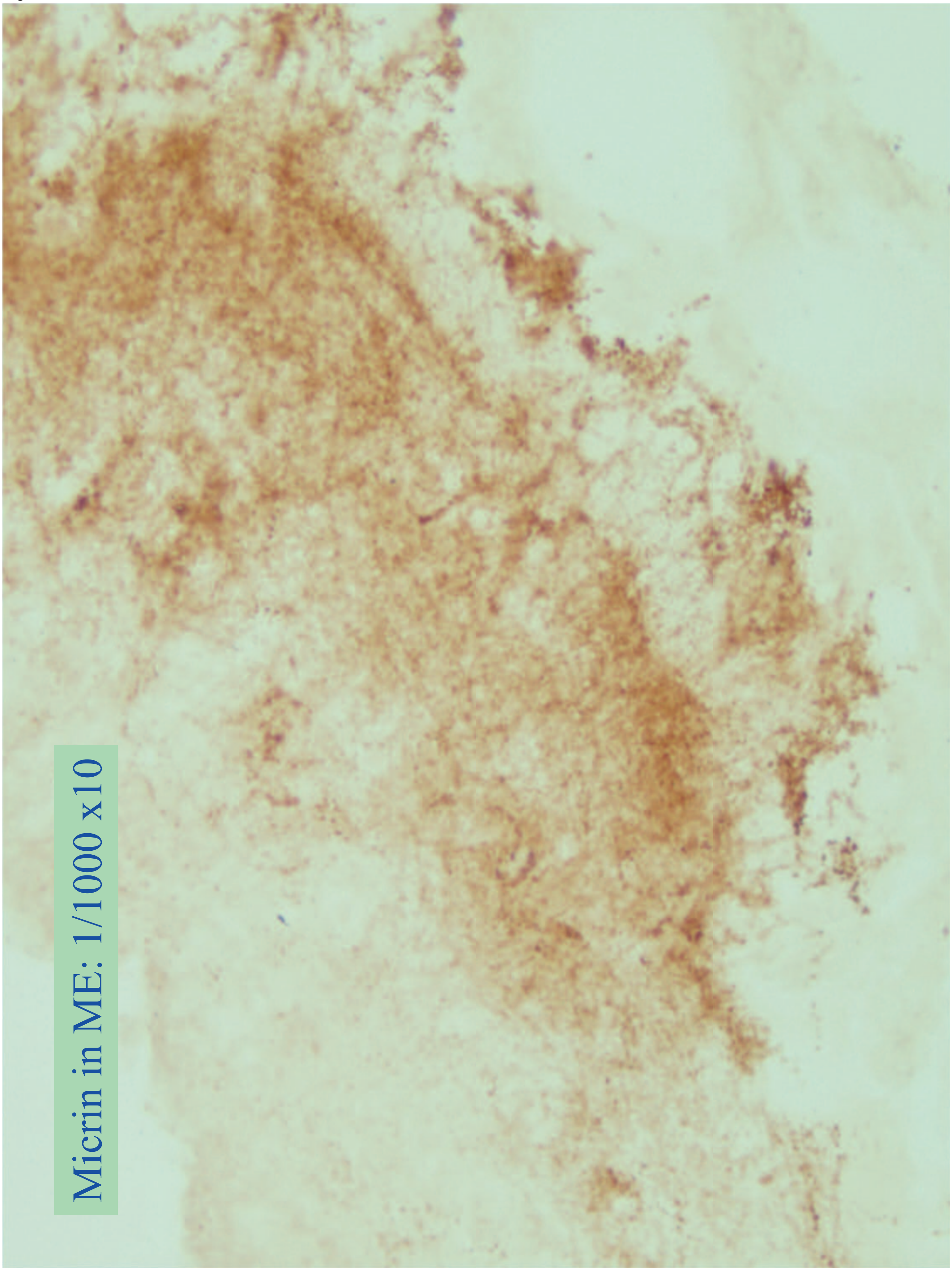

Figure 19

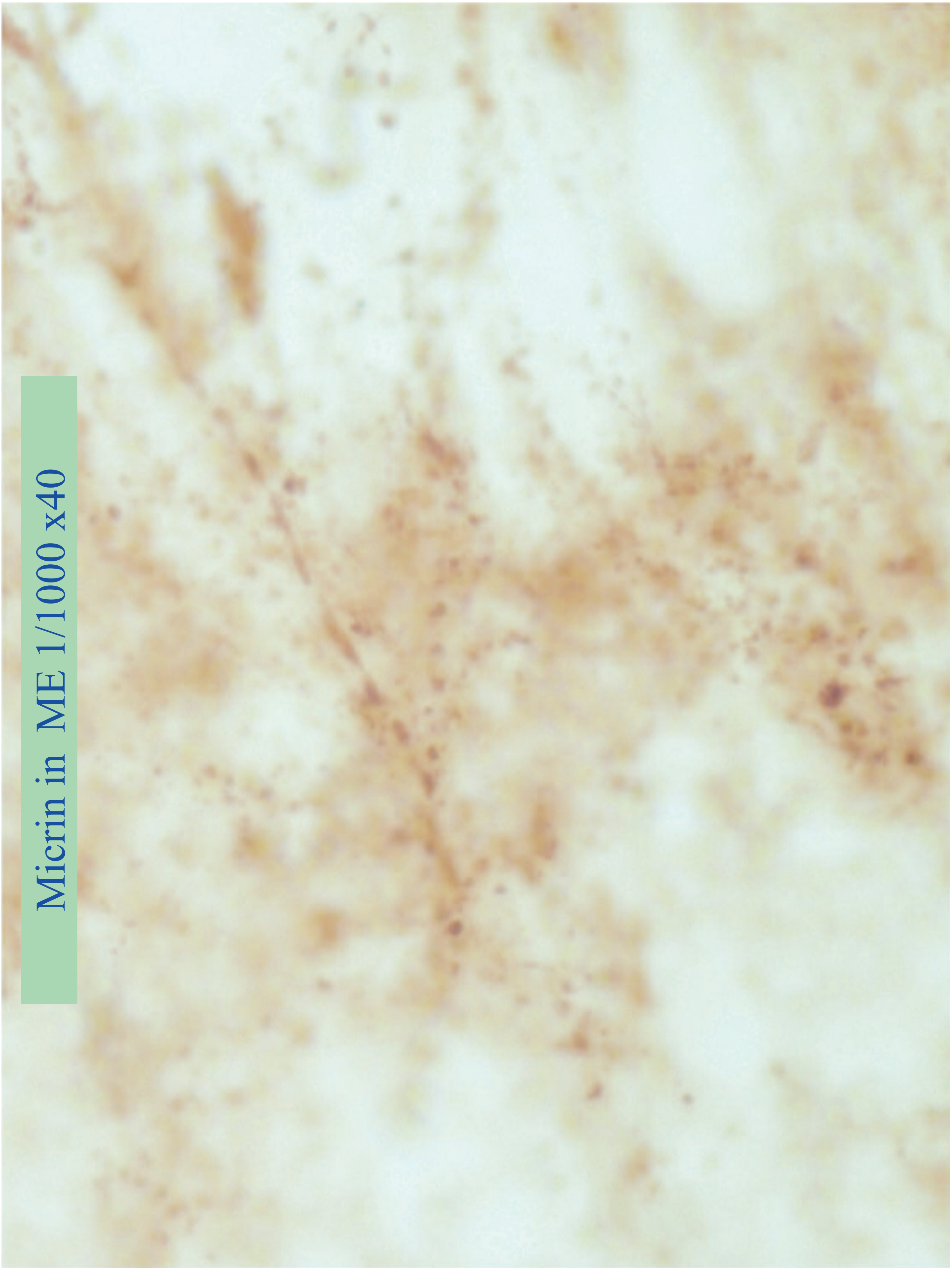

Figure 20

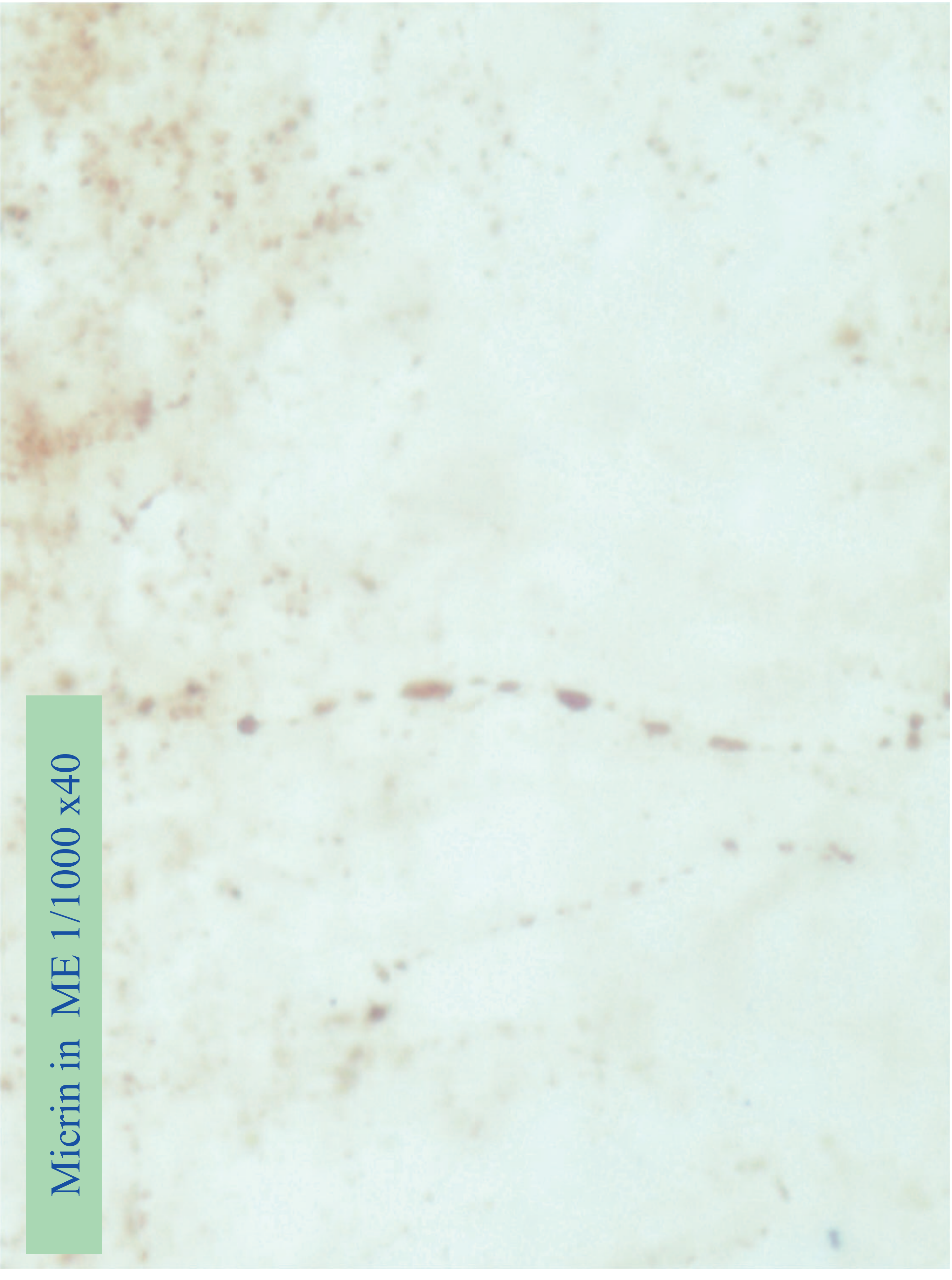

Micrin in ME x40

Figure 21

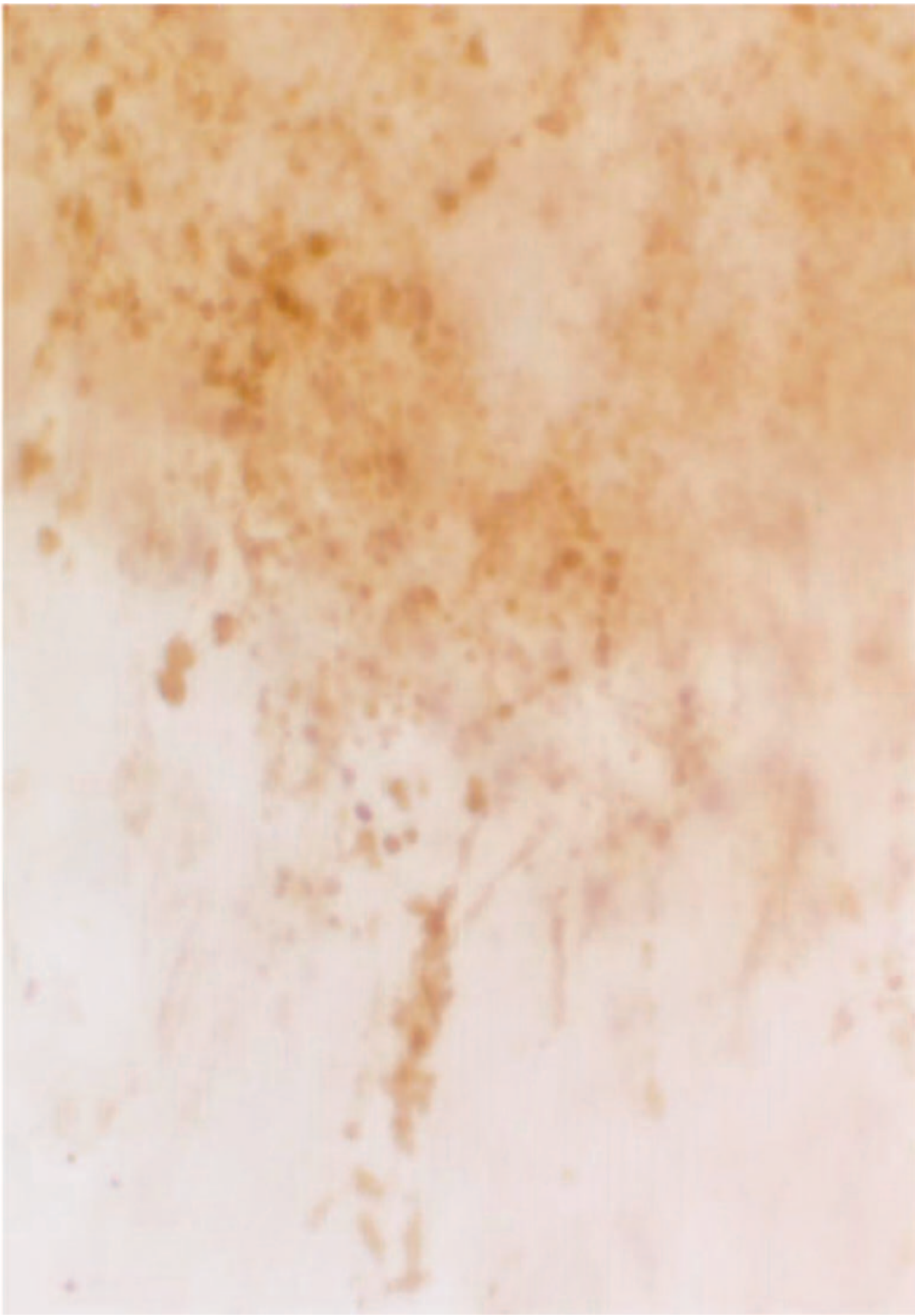

Figure 22

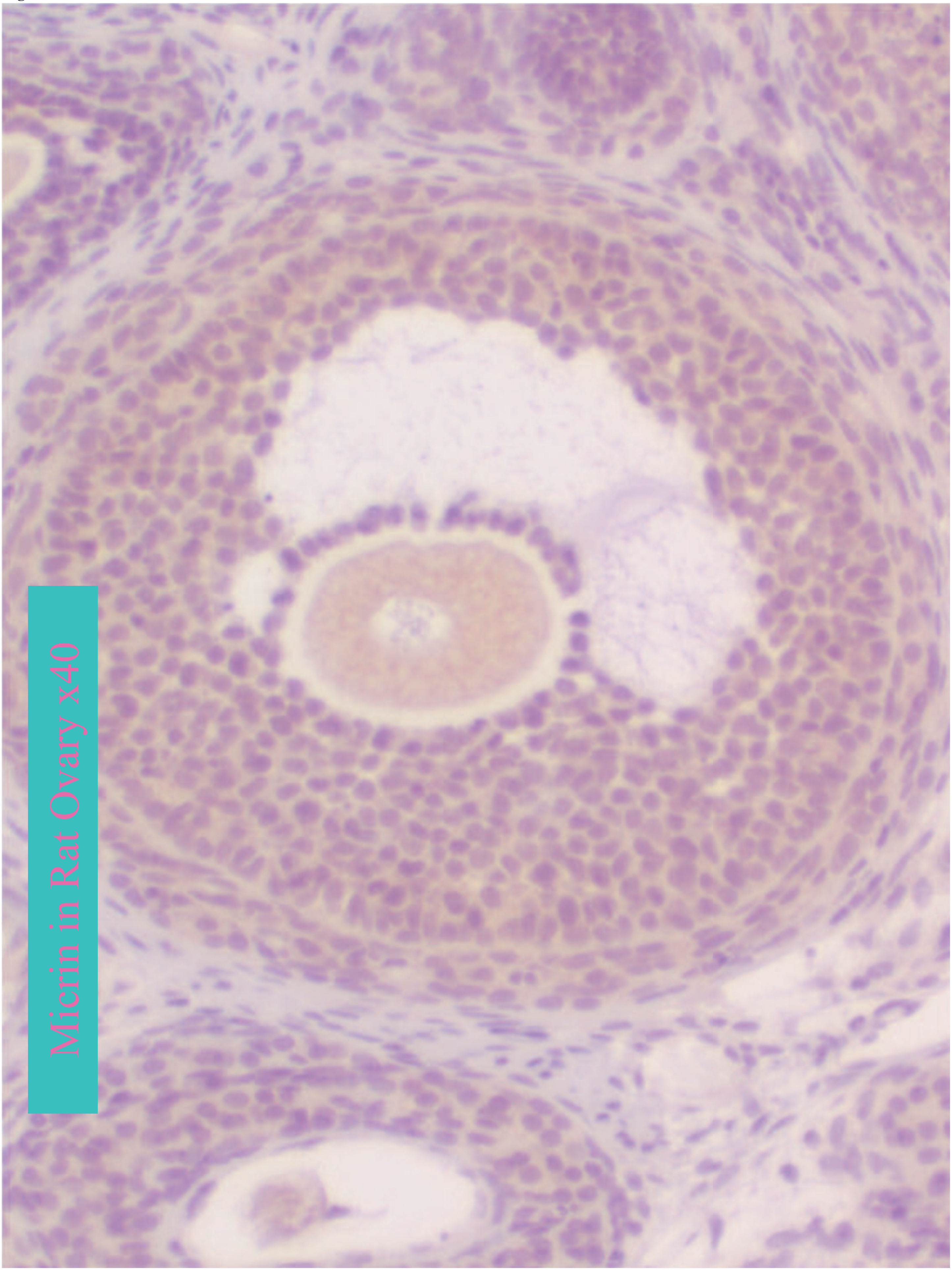

Figure 23

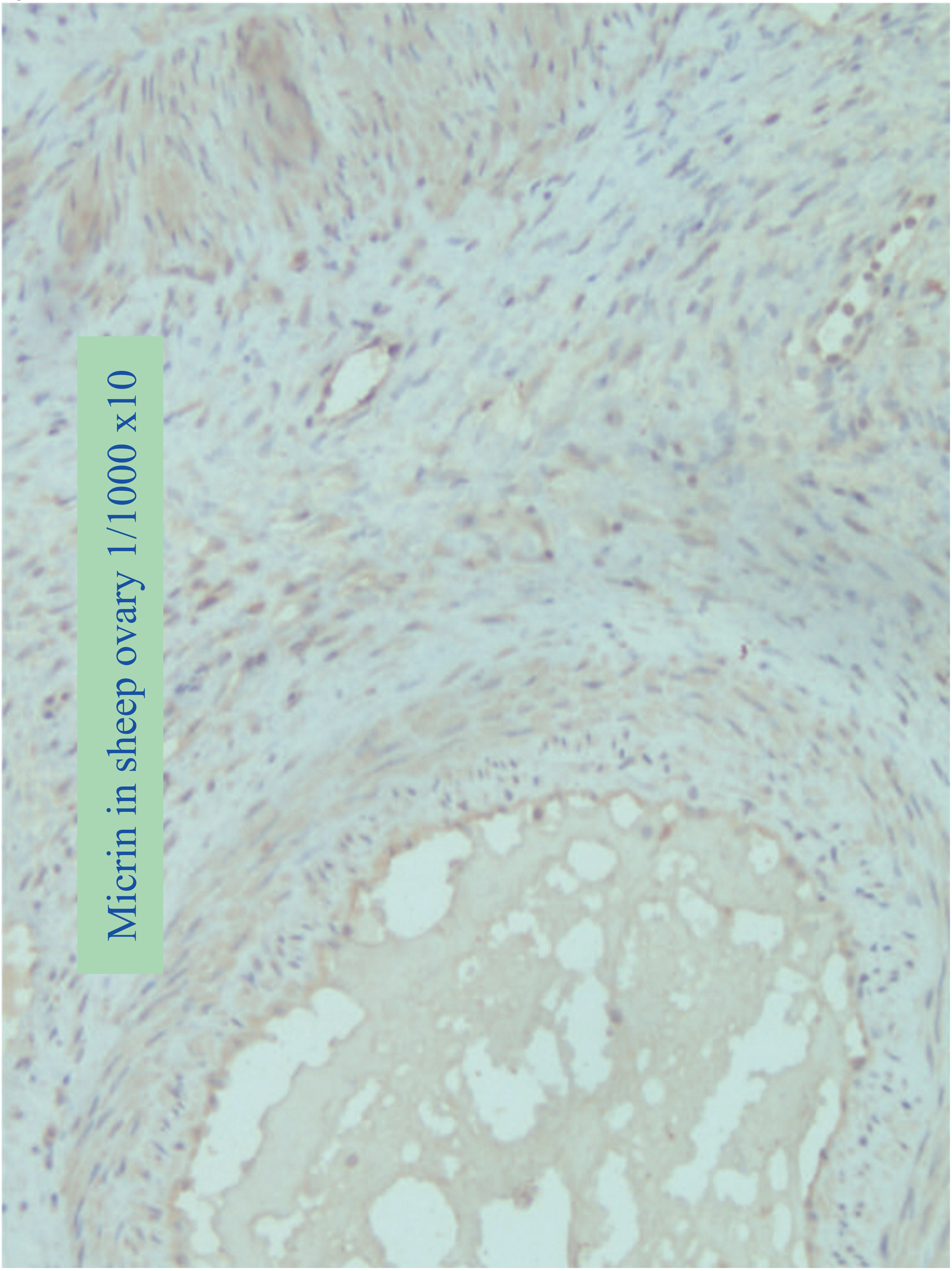

Figure 24

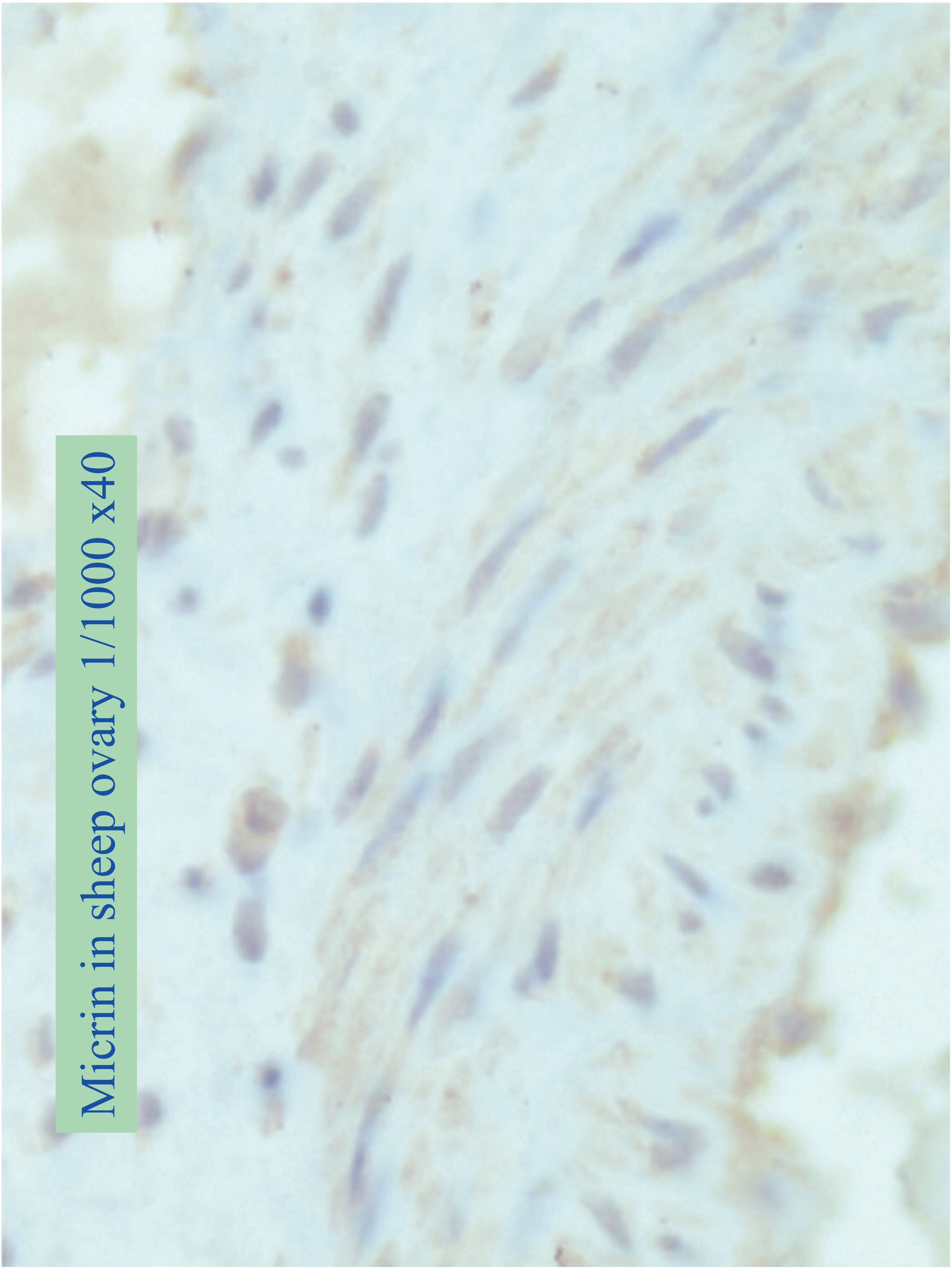

Figure 25

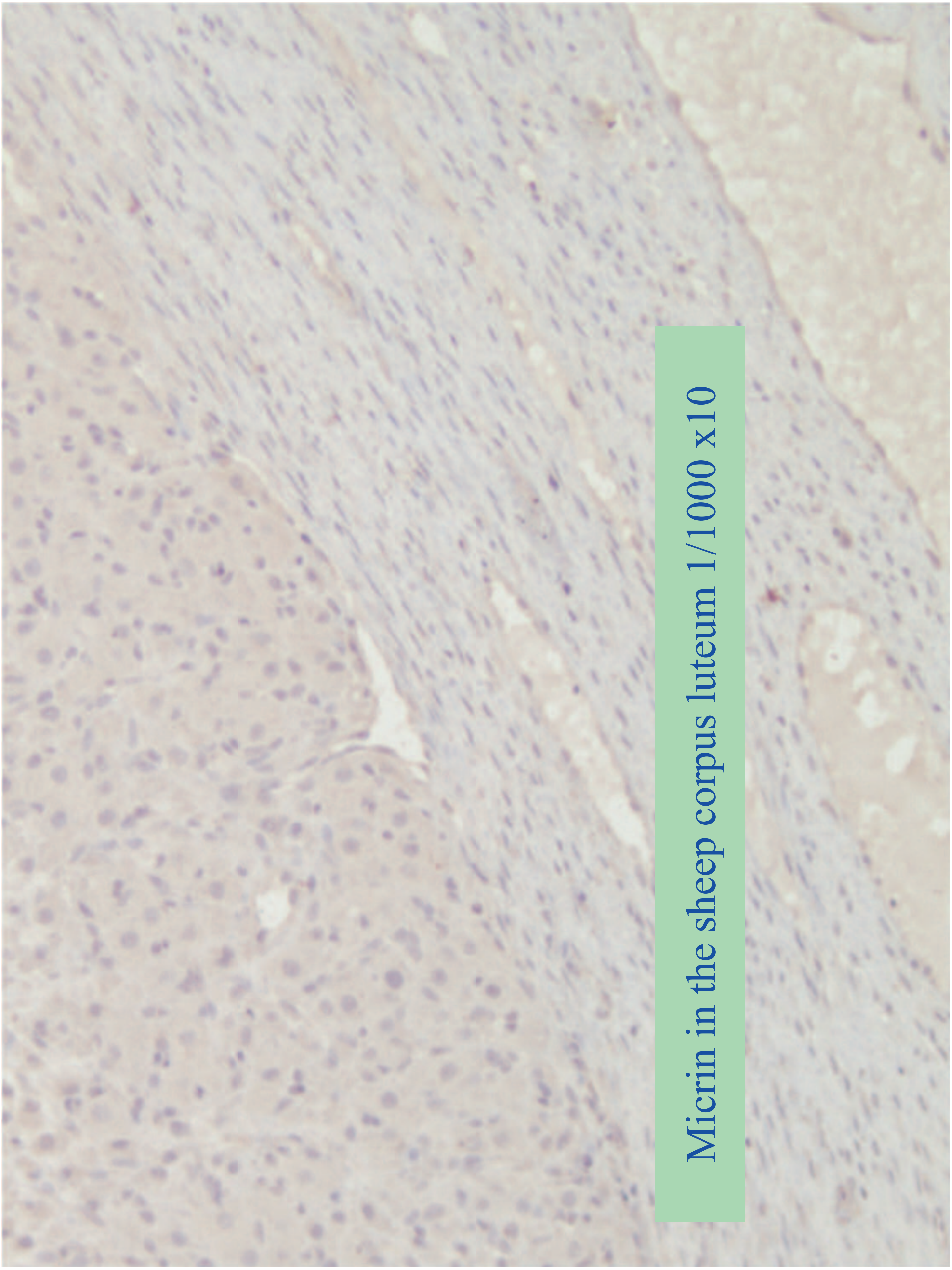

Figure 26

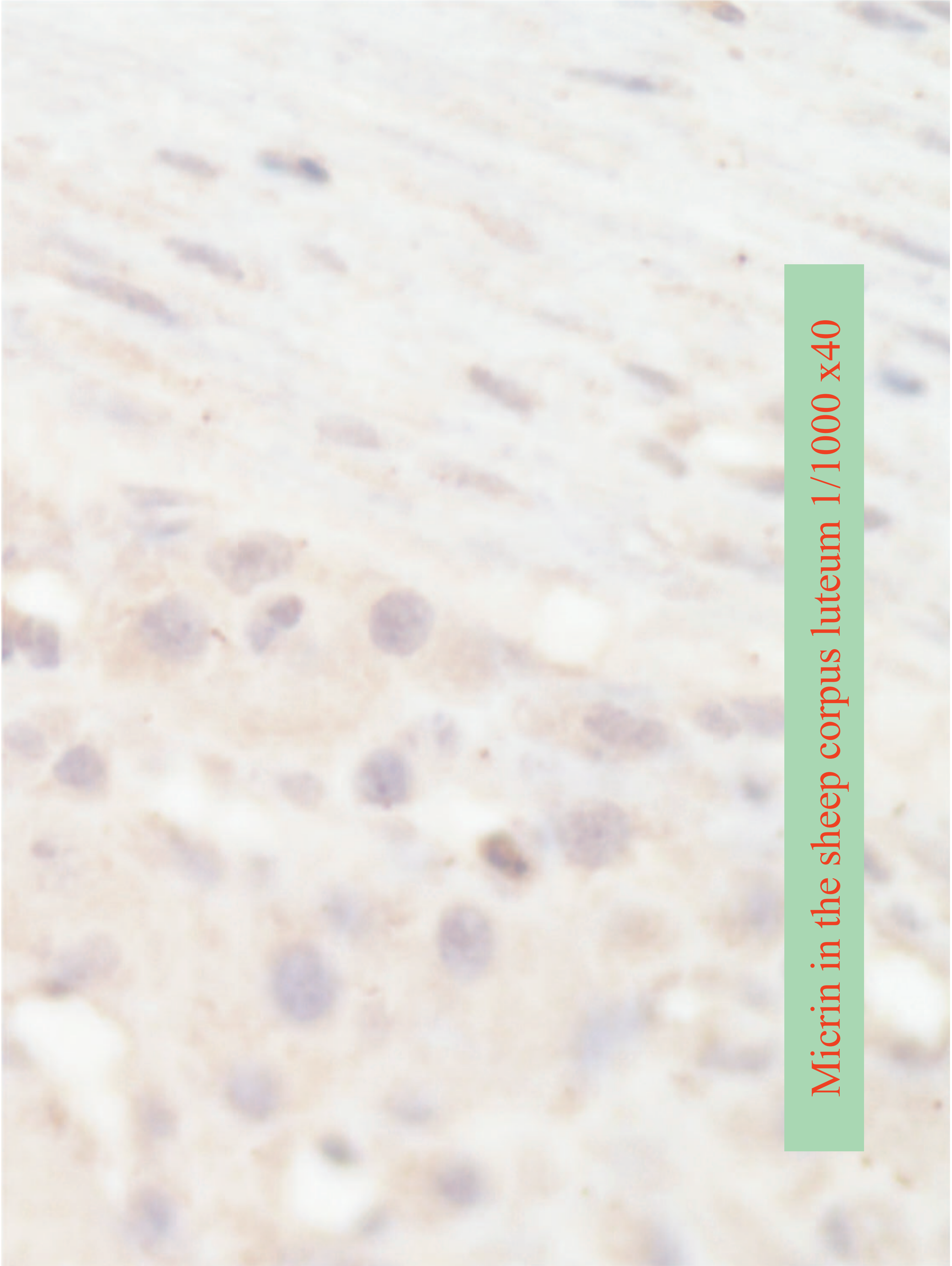

Figure 27

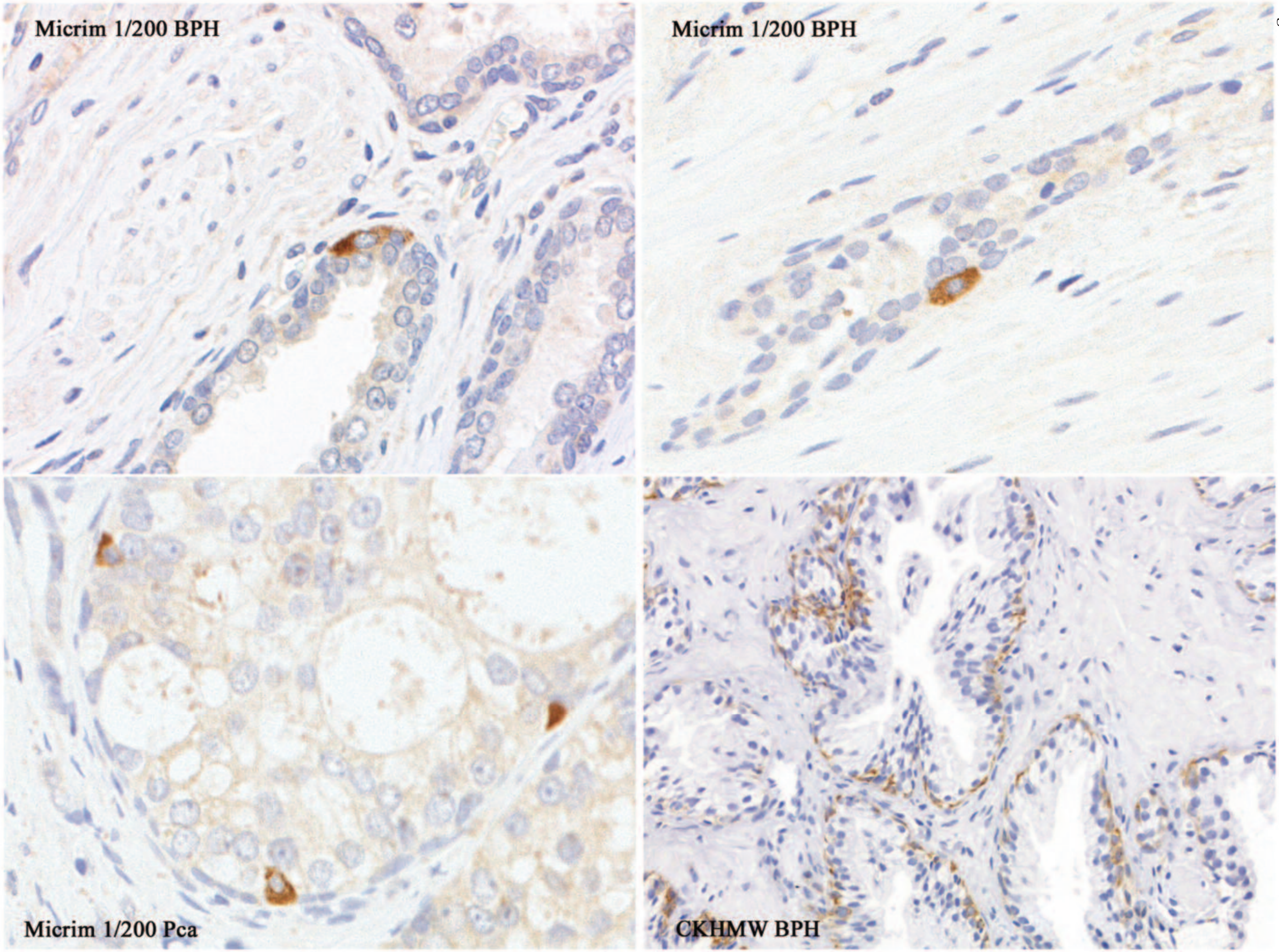

Human prostate   BPH = Benign prostatic hyperplasia Pca = Prostatic carcinoma
CKHMW = pan cyto-keratin (high molecular weight), a marker of epithelial cells in prostate

Micrin cells stained with ER88 in sheep PVN

Figure 28

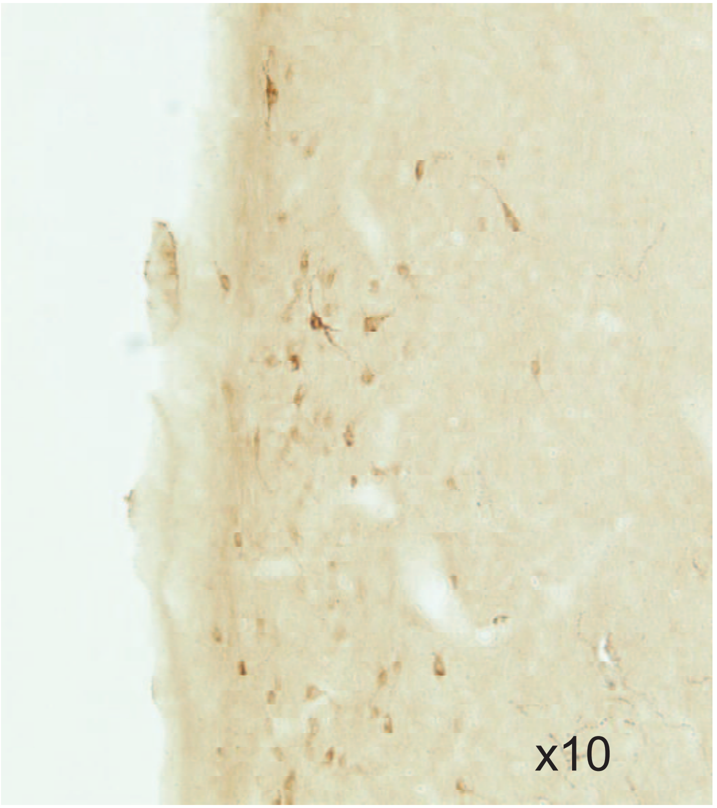

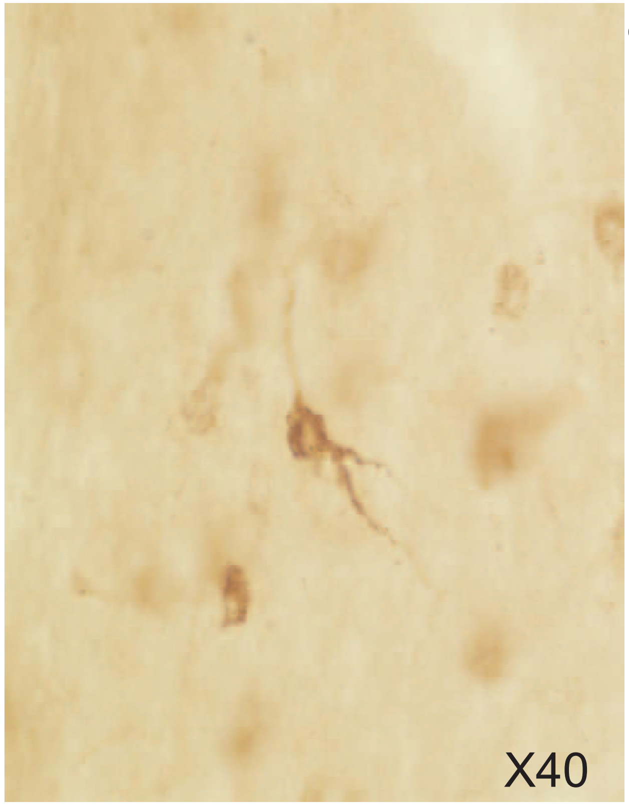

Figure 29

Micrin positive theca cells in normal human ovary

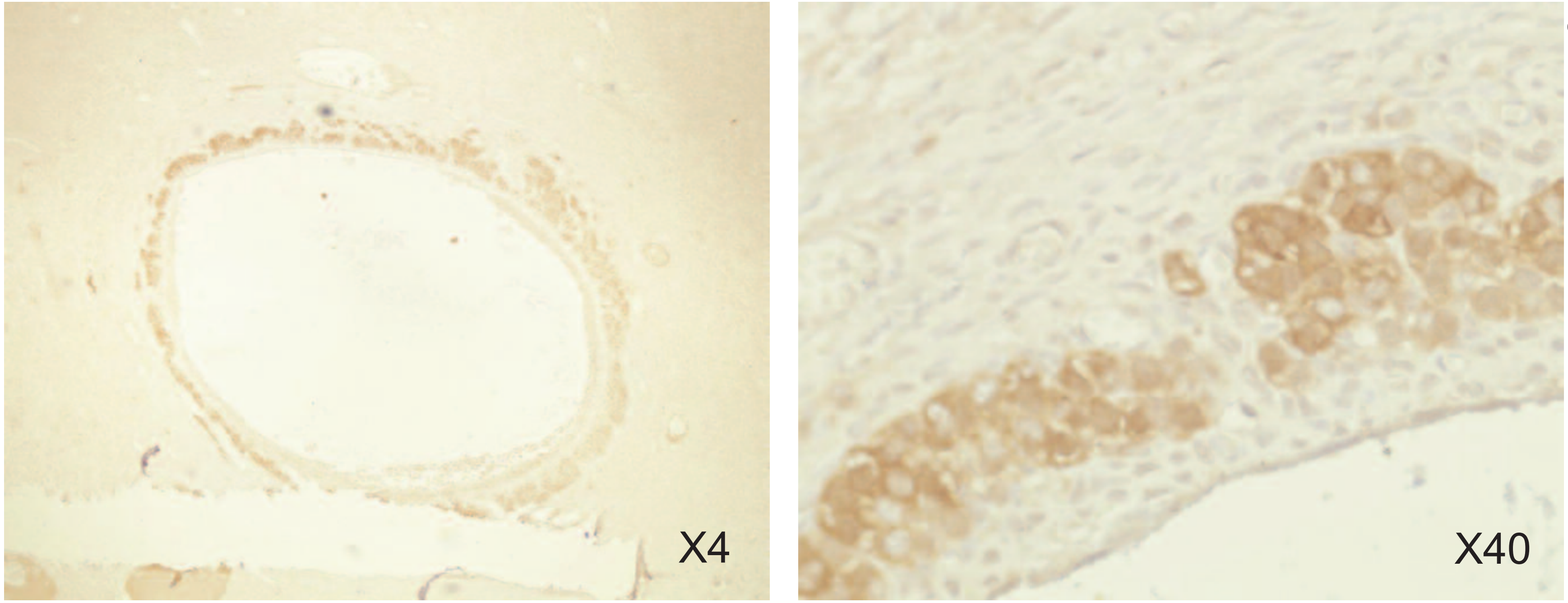

Figure 30

Micrin positive cells in mouse placenta

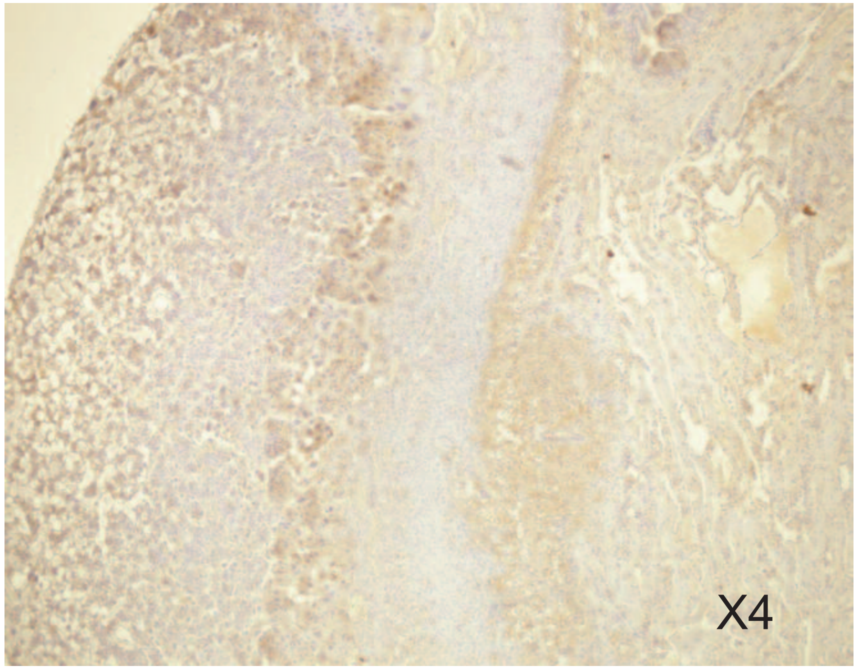

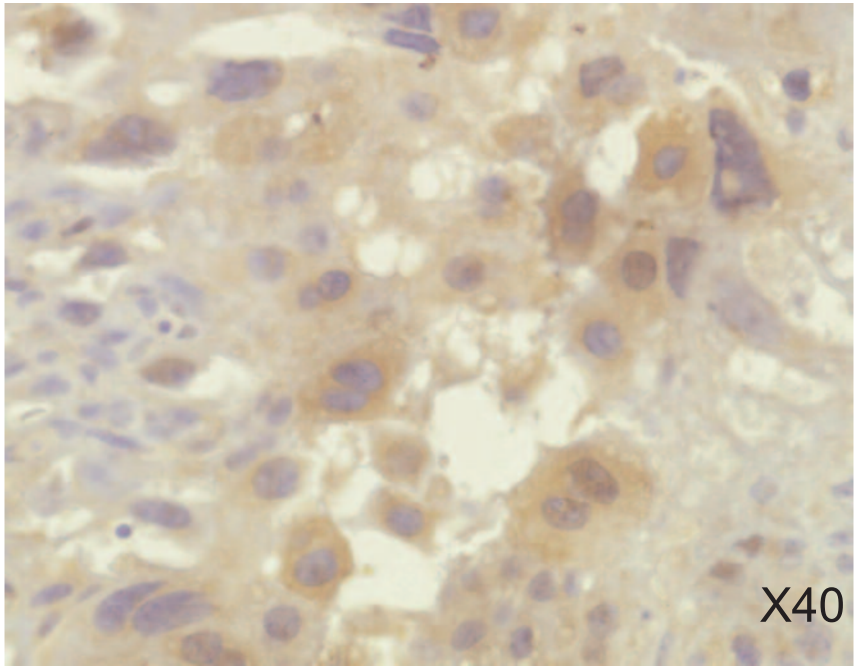

Figure 31

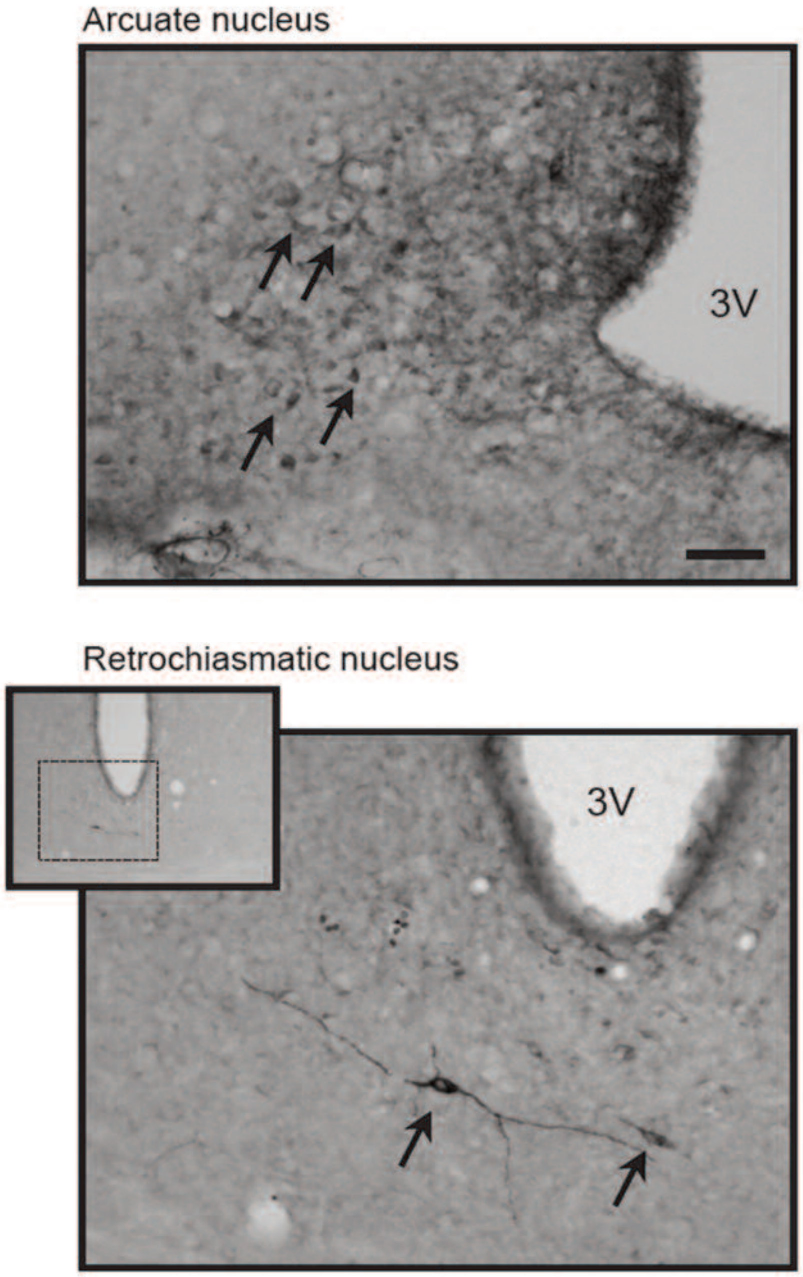

Photomicrograph of rat brain perfused with 4% paraformaldehyde and sectioned coronally at 40 μm. Sections blocked with normal rabbit serum; primary goat antibody diluted at 1:1000; incubation at 4 degrees C for 48 hours. Biotinylated rabbit anti-goat secondary (1:500 for 1 hour) and strep-HRP (1:500 for 1 hour). Colour developed using DAB (15min; Roche). Arrow points to immunopositive neurons located in regions of the hypothalamus. The white bay is the 3rd ventricle (‘3V’). Scale bar 50 μm

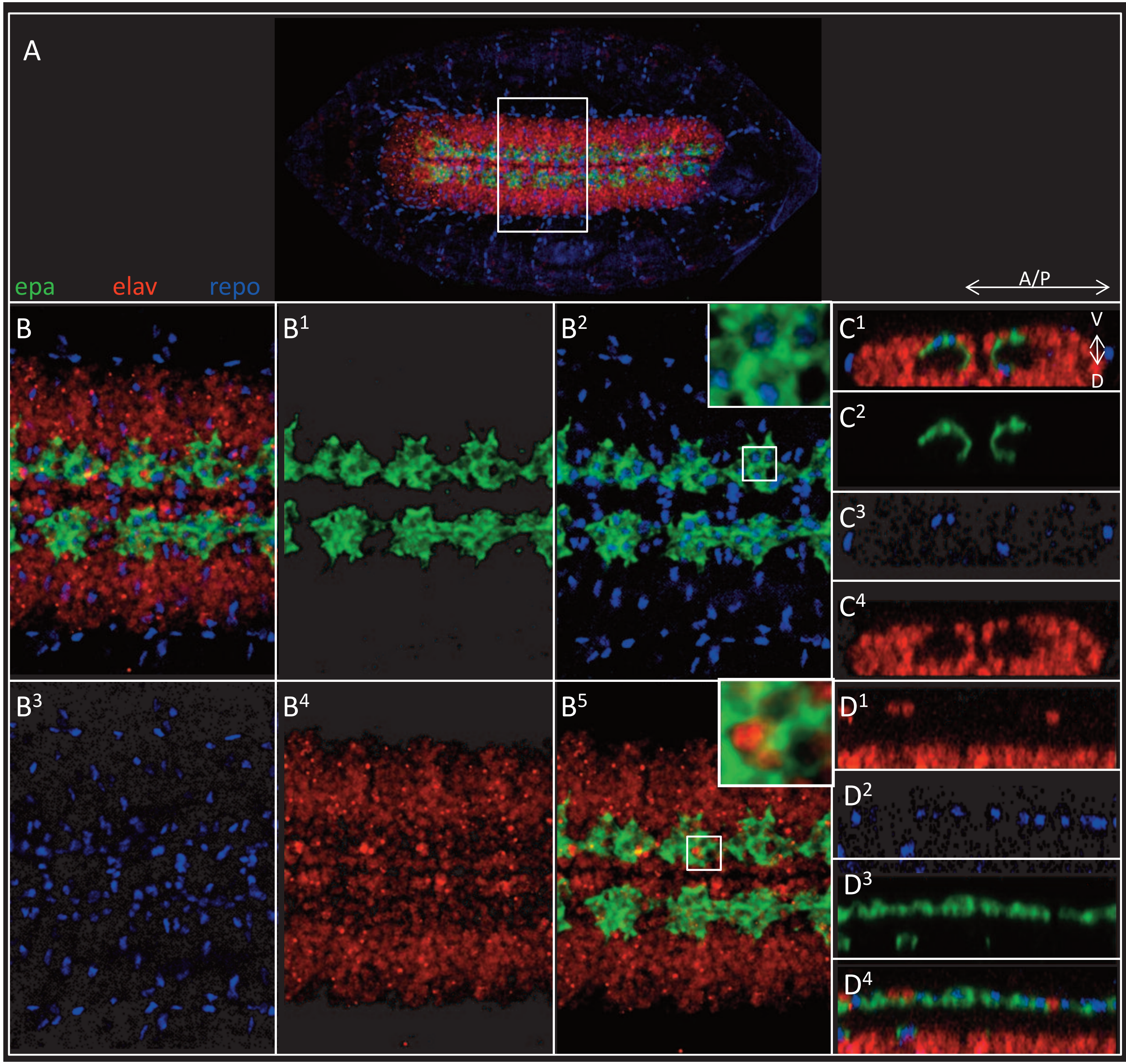

Figure 32. Epa co-occurs with a subset of glia cells in the *Drosophila* ventral cord

Panel ***(A)*** shows a view of the ventral cord at stage 16 on a wild type *Drosophila* embryo stained with antibodies against endocrine pharmaceutical's goat antibody (Epa) (in green), Elav (in red) , and Repo (in blue). Elav is a neural marker expressed in the nucleus of all neural cells . Repo is expressed in all glia. Epa marks the membrane of a sub set of cells expressing the Repo nuclear marker. Panels (***B, B^1, B^3 ,B^4***)contain zoomed inserts from ***(A)*** showing all channels merge and all individual channels. (***B^2***) shows co-occurring Epa and Repo positive cells (shown in insert). Although to a much less extent (***B^5***) shows Elav and Epa co-occurrance (shown in insert). ***(C^1 - C^4)*** is a digitaly re-arranged z-stack to show the coronal view of ***(A)*** . ***(D^1 - D^4)*** shows a lateral view of ***(A)*** after digital re-arrangement. Abbreviations V (ventral), D (dorsal), A/P (anterior/posterior)

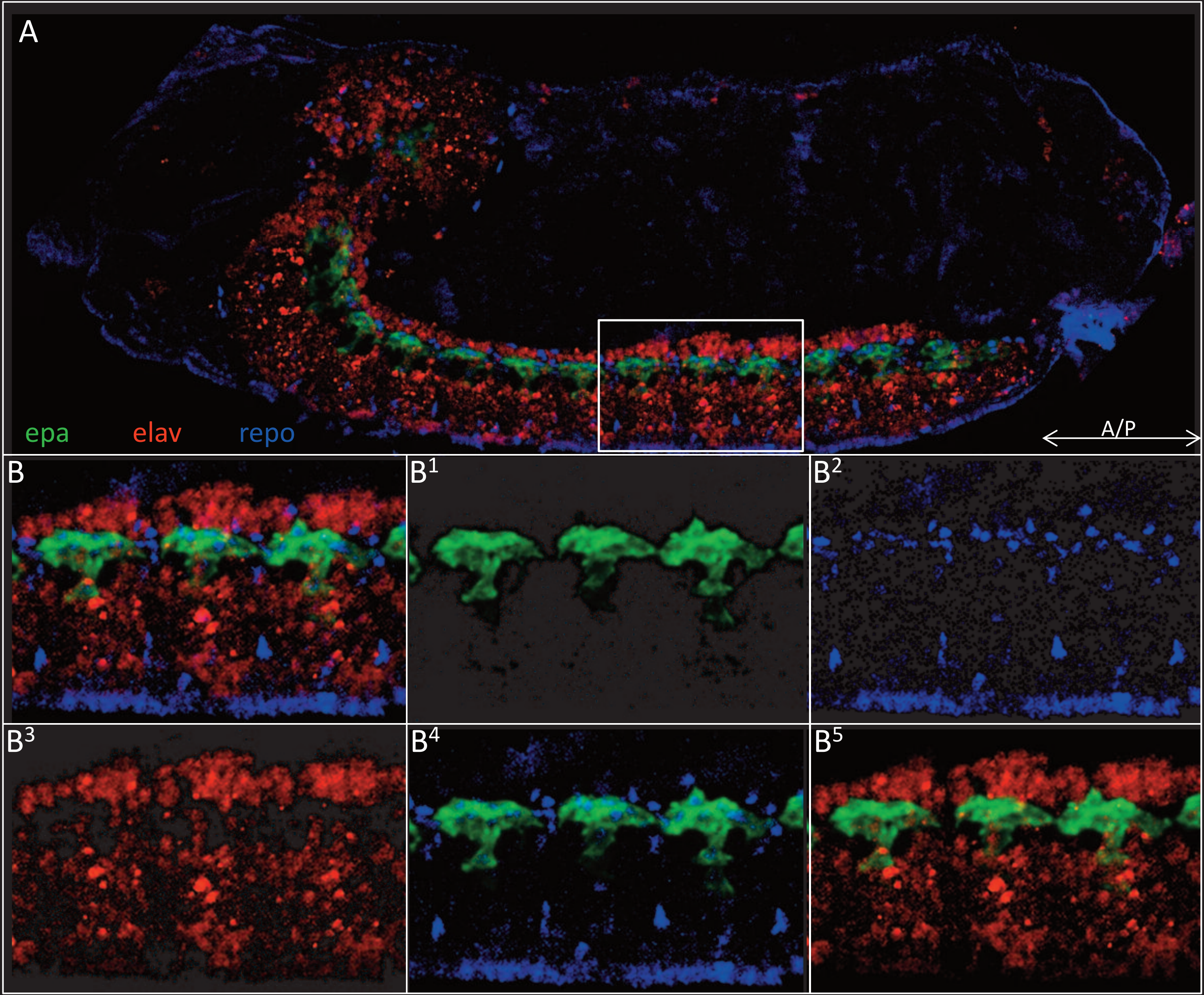

Figure 33. Lateral View

(A)) shows a lateral view of the ventral cord at stage 16 on a wild type *Drosophila* embryo stained with antibodies against endocrine pharmaceutical's goat antibody (Epa) (in green), Elav (in red) , and Repo (in blue). (***B-B***^{5}) shows zoomed inserts from ***(A)*** with all channels merge and all individual channels. Abbreviations V (ventral), D (dorsal), A/P (anterior/posterior

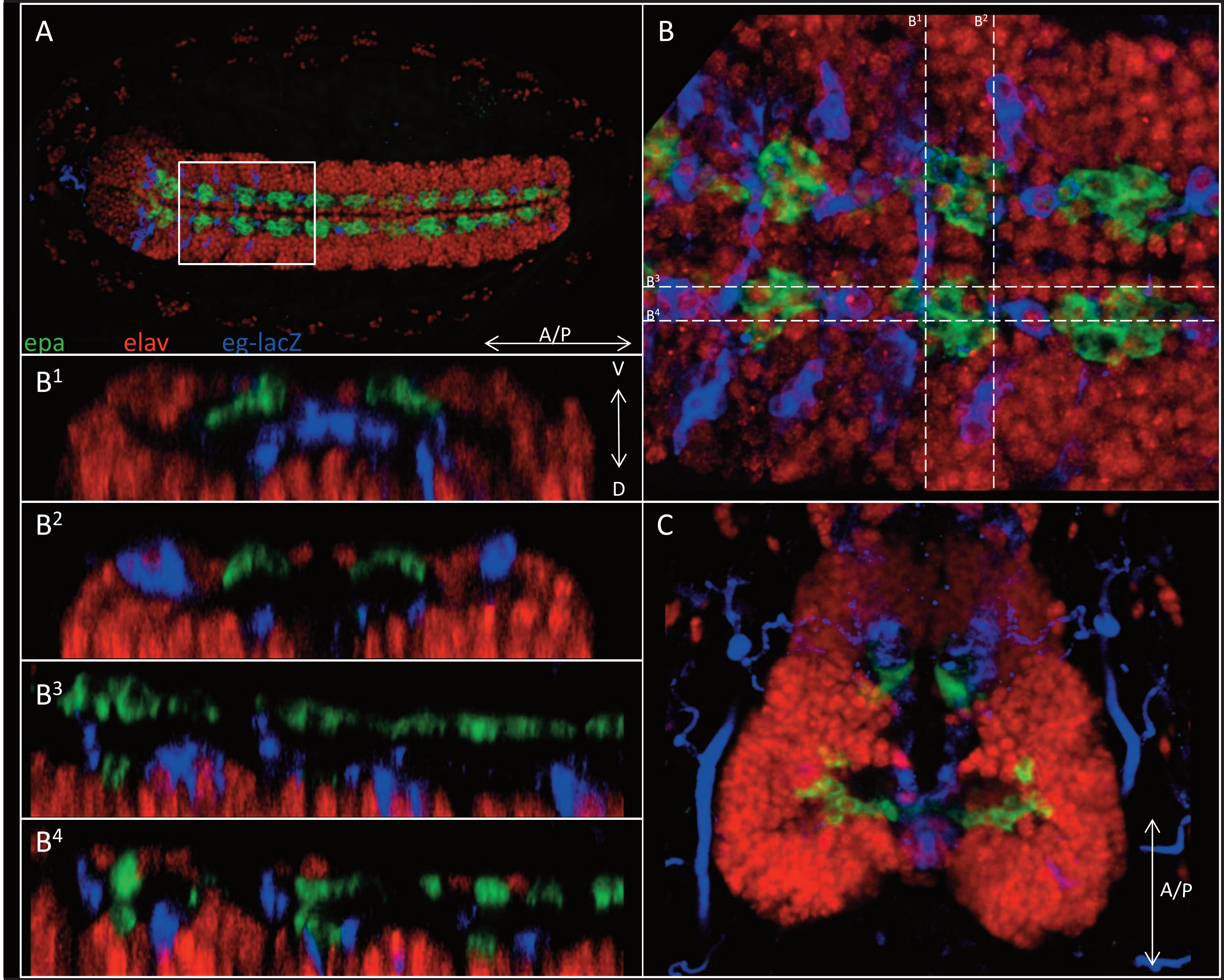

Figure 34. Epa antibody with respect to Eagle expression.

(A) shows a view of the ventral cord at stage 16 on an *eg-kinesin lacZ Drosophila* embryo stained with antibodies against endocrine pharmaceutical's goat antibody (Epa) (in green), Elav (in red), and lacZ (in blue). Eg (eagle) is a gene involved in determination of serotonergic neurons. (***B)*** shows a higher resolution picture from an area in ***(A)***. Panels ***(B-B⁴)*** contain digital re-slicing of ***(B)*** where white dotted lines are placed. ***(C)*** shows expression of Epa within the Drosophila brain (marked in red by Elav antibody). Abbreviations V (ventral), D (dorsal), A/P (anterior/posterior)

Figures 35

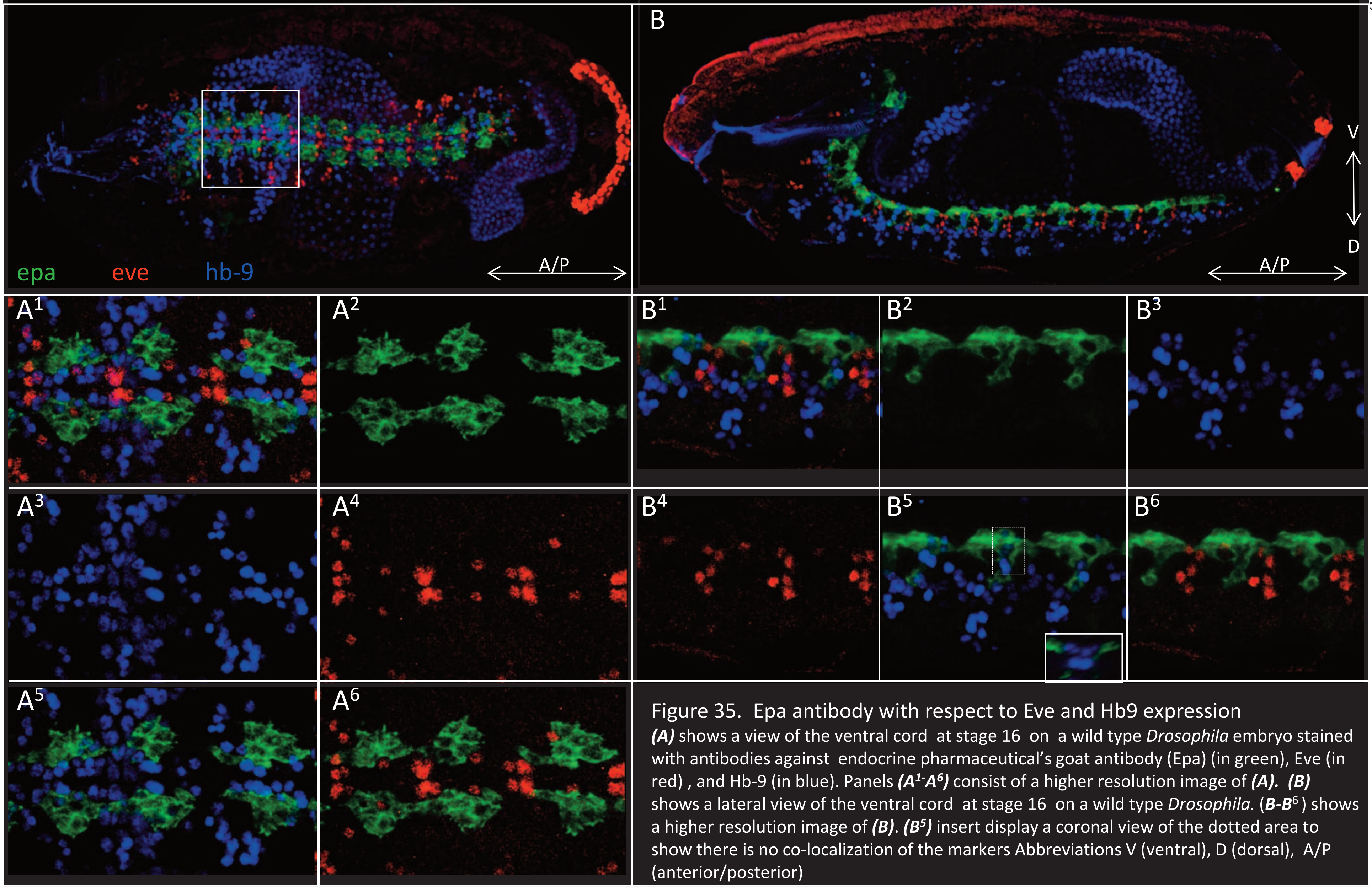

Figure 35. Epa antibody with respect to Eve and Hb9 expression
(A) shows a view of the ventral cord at stage 16 on a wild type *Drosophila* embryo stained with antibodies against endocrine pharmaceutical's goat antibody (Epa) (in green), Eve (in red), and Hb-9 (in blue). Panels ***(A^1-A^6)*** consist of a higher resolution image of ***(A)***. ***(B)*** shows a lateral view of the ventral cord at stage 16 on a wild type *Drosophila*. (***B-B^6***) shows a higher resolution image of ***(B)***. ***(B^5)*** insert display a coronal view of the dotted area to show there is no co-localization of the markers Abbreviations V (ventral), D (dorsal), A/P (anterior/posterior)

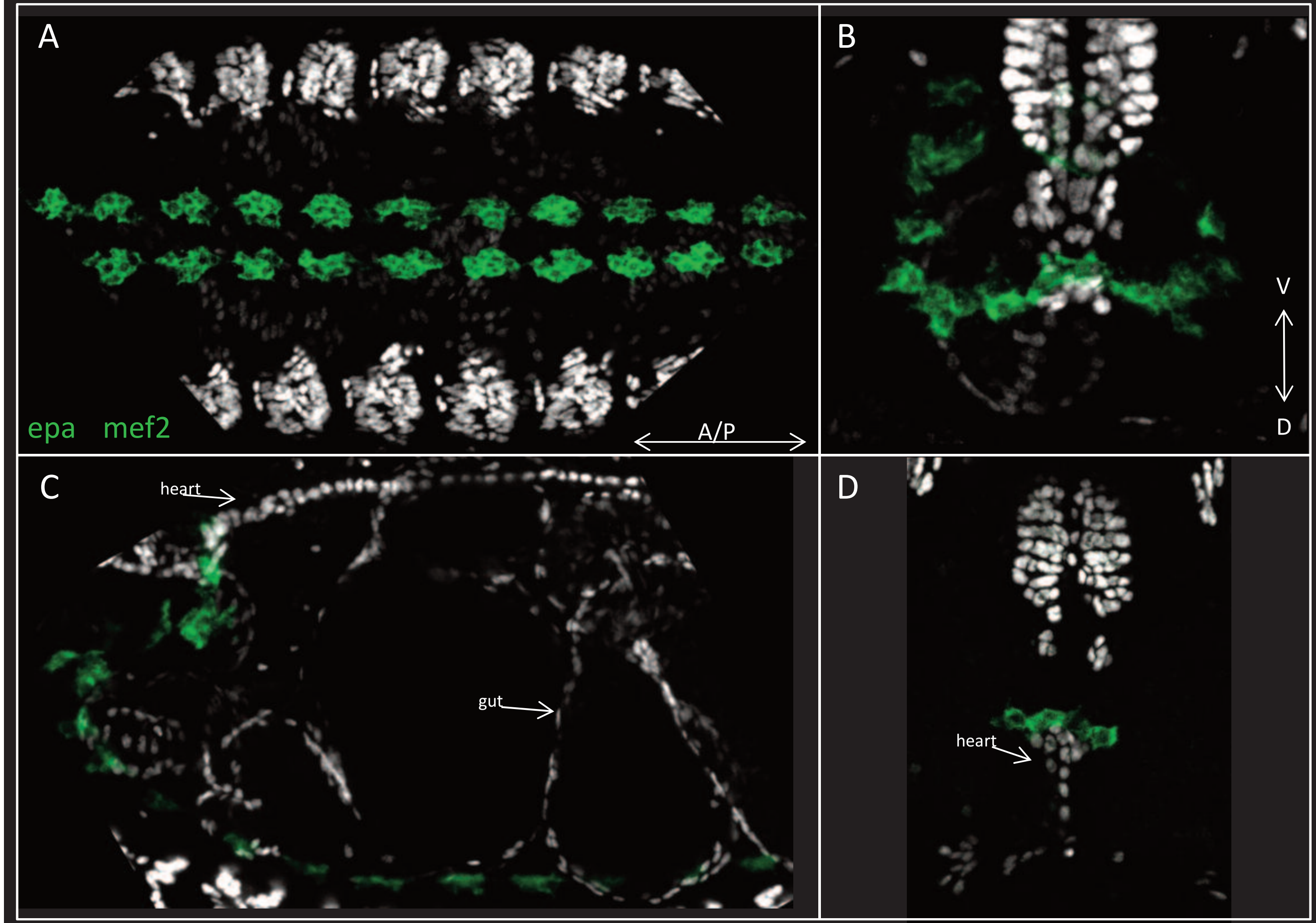

Figure 36. Epa antibody with respect to mef2 expression
(A) shows a view of the ventral cord at stage 16 on a wild type *Drosophila* embryo stained with antibodies against endocrine pharmaceutical's goat antibody (Epa) (in green) and Mef2 (in white). Mef2 is a transcription factor important during mesoderm and muscle development. Panels (***B)*** and ***(D)*** consist of two different z planes of the brain area (not marked). Abbreviations V (ventral), D (dorsal), A/P (anterior/posterior)

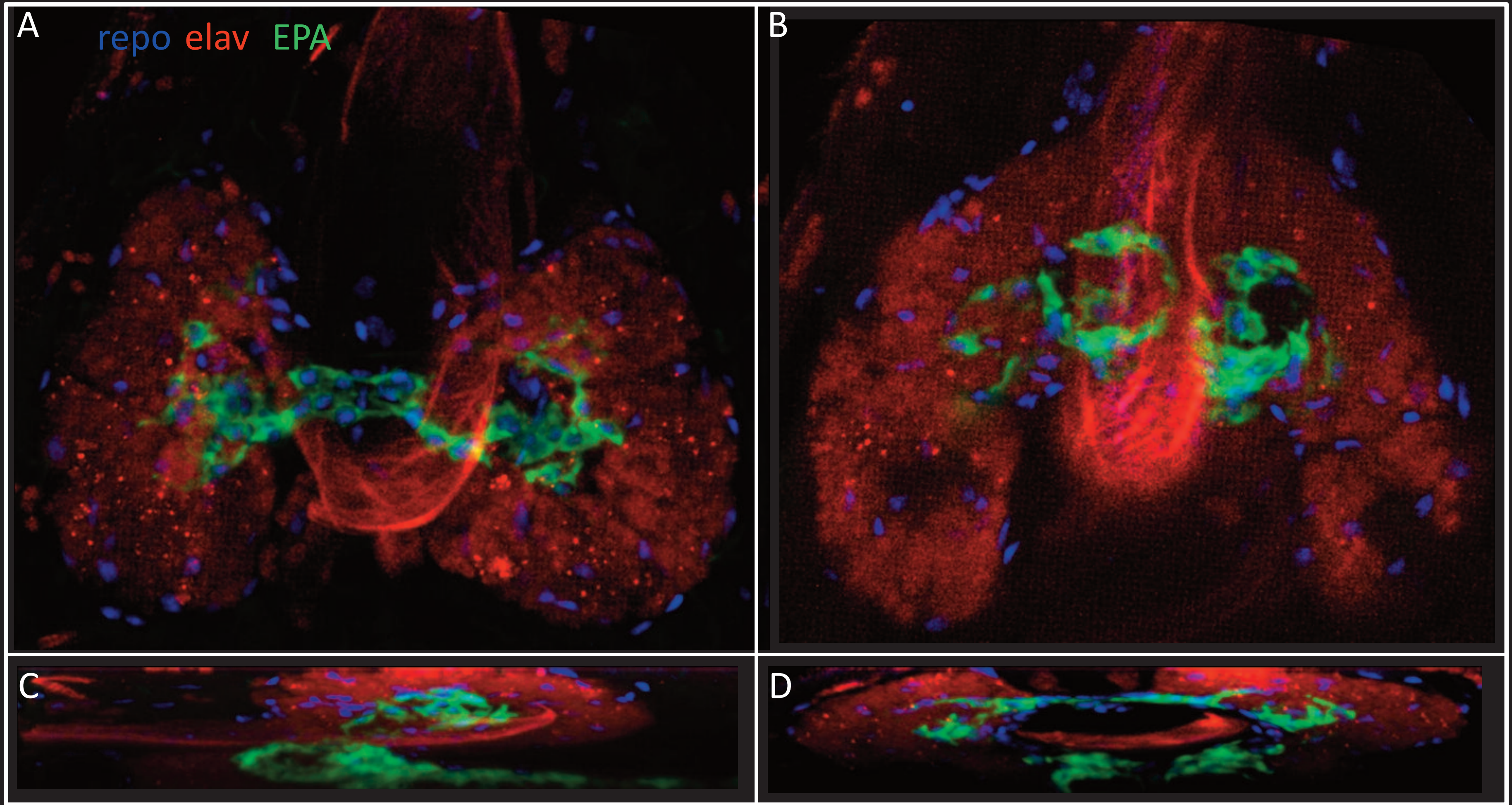

Fig. 37 Different views of the same drosophila embryo brain. EPA in green, elav antibody which marks all neural cells in red and repo antibody marking the glial cells in blue Again EPA co-localizes with repo. A) is a dorsal view of the brain. B) is a more ventral view. C) Is a digital re-slicing of the same image seen in A and B from the lateral view and D) from the transverse view

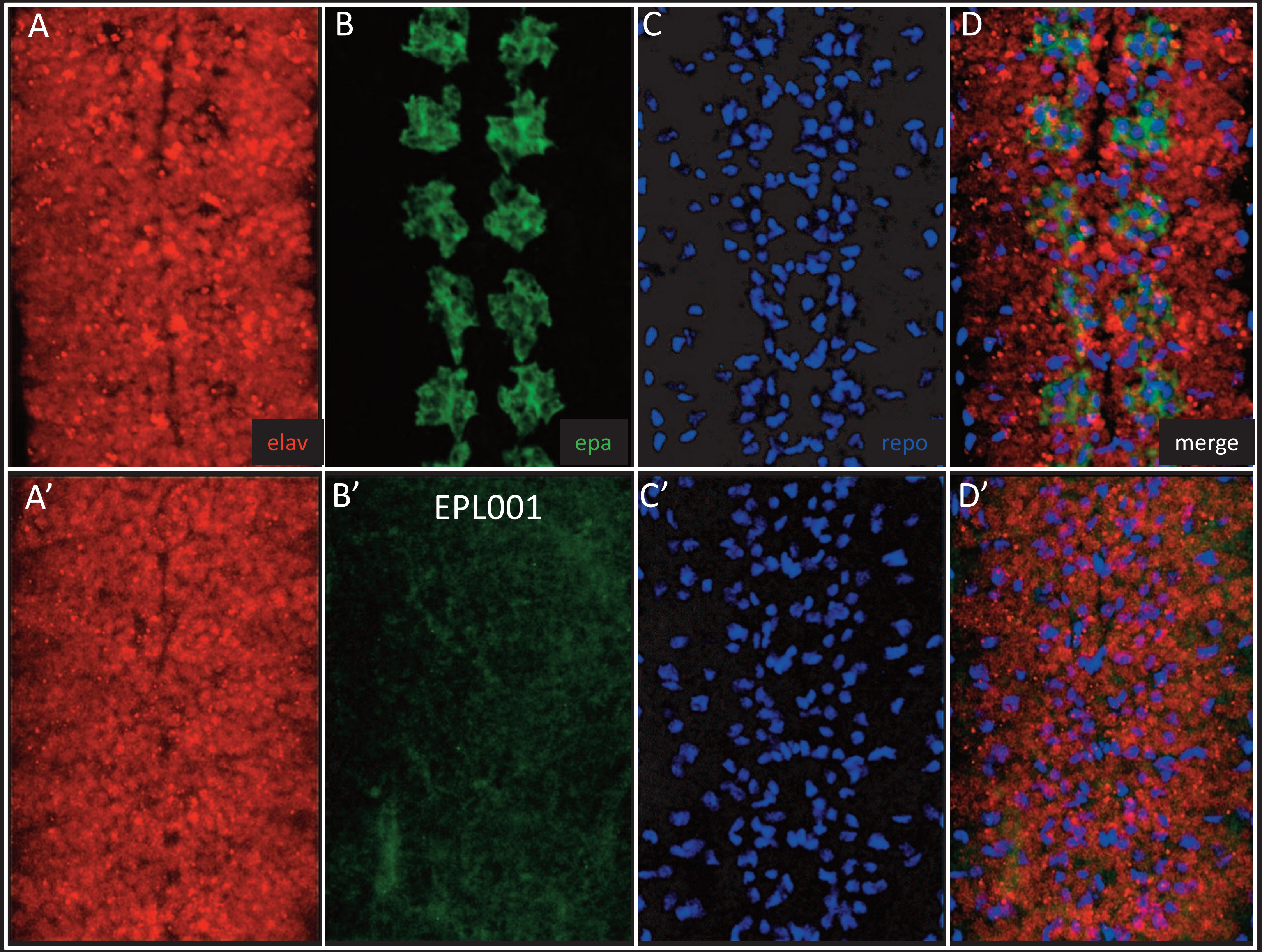

Fig. 38 Control experiment using EPL001
